# Clinical and Demographic Factors Associated With COVID-19, Severe COVID-19, and SARS-CoV-2 Infection in Adults: A Secondary Cross-Protocol Analysis of 4 Randomized Clinical Trials

**DOI:** 10.1001/jamanetworkopen.2023.23349

**Published:** 2023-07-13

**Authors:** Deborah A. Theodore, Angela R. Branche, Lily Zhang, Daniel S. Graciaa, Madhu Choudhary, Timothy J. Hatlen, Raadhiya Osman, Tara M. Babu, Samuel T. Robinson, Peter B. Gilbert, Dean Follmann, Holly Janes, James G. Kublin, Lindsey R. Baden, Paul Goepfert, Glenda E. Gray, Beatriz Grinsztejn, Karen L. Kotloff, Cynthia L. Gay, Brett Leav, Jacqueline Miller, Ian Hirsch, Jerald Sadoff, Lisa M. Dunkle, Kathleen M. Neuzil, Lawrence Corey, Ann R. Falsey, Hana M. El Sahly, Magdalena E. Sobieszczyk, Yunda Huang

**Affiliations:** 1 Division of Infectious Diseases, Department of Medicine, Columbia University Irving Medical Center, New York, New York; 2 Department of Medicine, Infectious Disease Division, University of Rochester, Rochester, New York; 3 Vaccine and Infectious Disease Division, Fred Hutchinson Cancer Center, Seattle, Washington; 4 Division of Infectious Diseases, Department of Medicine, Emory University School of Medicine, Atlanta, Georgia; 5 Division of Infectious Diseases, University of Pittsburgh School of Medicine, Pittsburgh, Pennsylvania; 6 Division of HIV, Harbor-UCLA Medical Center, Torrance, California; 7 Perinatal HIV Research Unit, Chris Hani Baragwanath Academic Hospital, Soweto, South Africa; 8 Department of Medicine, Division of Allergy & Infectious Diseases, University of Washington, Seattle; 9 Department of Biostatistics, University of Washington, Seattle; 10 Biostatistics Research Branch, National Institute of Allergy and Infectious Disease, National Institutes of Health, Bethesda, Maryland; 11 Brigham and Women’s Hospital, Boston, Massachusetts; 12 Division of Infectious Diseases, Department of Medicine, University of Alabama at Birmingham, Birmingham; 13 Perinatal HIV Research Unit, Faculty of Health Sciences, University of the Witwatersrand, Johannesburg, South Africa; 14 South African Medical Research Council, Cape Town, South Africa; 15 Evandro Chagas National Institute of Infectious Diseases-Fundação Oswaldo Cruz, Rio de Janeiro, Brazil; 16 Division of Infectious Disease and Tropical Pediatrics, Department of Pediatrics, University of Maryland School of Medicine, Baltimore; 17 Department of Medicine, Center for Vaccine Development and Global Health, University of Maryland School of Medicine, Baltimore; 18 Department of Medicine, Division of Infectious Diseases, UNC HIV Cure Center, University of North Carolina at Chapel Hill School of Medicine, Chapel Hill; 19 Moderna, Cambridge, Massachusetts; 20 AstraZeneca BioPharmaceuticals, Cambridge, United Kingdom; 21 Janssen Vaccines and Prevention, Leiden, the Netherlands; 22 Novavax, Gaithersburg, Maryland; 23 Department of Laboratory Medicine and Pathology, University of Washington, Seattle; 24 Infectious Diseases Section, Department of Medicine, Baylor College of Medicine, Houston, Texas; 25 Department of Molecular Virology and Microbiology, Baylor College of Medicine, Houston, Texas; 26 Department of Global Health, University of Washington, Seattle

## Abstract

**Importance:**

Current data identifying COVID-19 risk factors lack standardized outcomes and insufficiently control for confounders.

**Objective:**

To identify risk factors associated with COVID-19, severe COVID-19, and SARS-CoV-2 infection.

**Design, Setting, and Participants:**

This secondary cross-protocol analysis included 4 multicenter, international, randomized, blinded, placebo-controlled, COVID-19 vaccine efficacy trials with harmonized protocols established by the COVID-19 Prevention Network. Individual-level data from participants randomized to receive placebo within each trial were combined and analyzed. Enrollment began July 2020 and the last data cutoff was in July 2021. Participants included adults in stable health, at risk for SARS-CoV-2, and assigned to the placebo group within each vaccine trial. Data were analyzed from April 2022 to February 2023.

**Exposures:**

Comorbid conditions, demographic factors, and SARS-CoV-2 exposure risk at the time of enrollment.

**Main Outcomes and Measures:**

Coprimary outcomes were COVID-19 and severe COVID-19. Multivariate Cox proportional regression models estimated adjusted hazard ratios (aHRs) and 95% CIs for baseline covariates, accounting for trial, region, and calendar time. Secondary outcomes included severe COVID-19 among people with COVID-19, subclinical SARS-CoV-2 infection, and SARS-CoV-2 infection.

**Results:**

A total of 57 692 participants (median [range] age, 51 [18-95] years; 11 720 participants [20.3%] aged ≥65 years; 31 058 participants [53.8%] assigned male at birth) were included. The analysis population included 3270 American Indian or Alaska Native participants (5.7%), 7849 Black or African American participants (13.6%), 17 678 Hispanic or Latino participants (30.6%), and 40 745 White participants (70.6%). Annualized incidence was 13.9% (95% CI, 13.3%-14.4%) for COVID-19 and 2.0% (95% CI, 1.8%-2.2%) for severe COVID-19. Factors associated with increased rates of COVID-19 included workplace exposure (high vs low: aHR, 1.35 [95% CI, 1.16-1.58]; medium vs low: aHR, 1.41 [95% CI, 1.21-1.65]; *P* < .001) and living condition risk (very high vs low risk: aHR, 1.41 [95% CI, 1.21-1.66]; medium vs low risk: aHR, 1.19 [95% CI, 1.08-1.32]; *P* < .001). Factors associated with decreased rates of COVID-19 included previous SARS-CoV-2 infection (aHR, 0.13 [95% CI, 0.09-0.19]; *P* < .001), age 65 years or older (aHR vs age <65 years, 0.57 [95% CI, 0.50-0.64]; *P* < .001) and Black or African American race (aHR vs White race, 0.78 [95% CI, 0.67-0.91]; *P* = .002). Factors associated with increased rates of severe COVID-19 included race (American Indian or Alaska Native vs White: aHR, 2.61 [95% CI, 1.85-3.69]; multiracial vs White: aHR, 2.19 [95% CI, 1.50-3.20]; *P* < .001), diabetes (aHR, 1.54 [95% CI, 1.14-2.08]; *P* = .005) and at least 2 comorbidities (aHR vs none, 1.39 [95% CI, 1.09-1.76]; *P* = .008). In analyses restricted to participants who contracted COVID-19, increased severe COVID-19 rates were associated with age 65 years or older (aHR vs <65 years, 1.75 [95% CI, 1.32-2.31]; *P* < .001), race (American Indian or Alaska Native vs White: aHR, 1.98 [95% CI, 1.38-2.83]; Black or African American vs White: aHR, 1.49 [95% CI, 1.03-2.14]; multiracial: aHR, 1.81 [95% CI, 1.21-2.69]; overall *P* = .001), body mass index (aHR per 1-unit increase, 1.03 [95% CI, 1.01-1.04]; *P* = .001), and diabetes (aHR, 1.85 [95% CI, 1.37-2.49]; *P* < .001). Previous SARS-CoV-2 infection was associated with decreased severe COVID-19 rates (aHR, 0.04 [95% CI, 0.01-0.14]; *P* < .001).

**Conclusions and Relevance:**

In this secondary cross-protocol analysis of 4 randomized clinical trials, exposure and demographic factors had the strongest associations with outcomes; results could inform mitigation strategies for SARS-CoV-2 and viruses with comparable epidemiological characteristics.

## Key Points

**Question:**

What clinical and demographic factors are associated with rates of COVID-19, severe COVID-19, and SARS-CoV-2 infection?

**Findings:**

In this secondary analysis of 57 692 participants randomized to the placebo groups of 4 COVID-19 vaccine phase 3 efficacy trials, exposure risks, demographics (age and race), and evidence of previous infection had the strongest associations with study outcomes.

**Meaning:**

These findings could inform public health policy pertaining to prioritization for vaccination and risk mitigation efforts.

## Teaser

This secondary analysis of 4 randomized clinical trials evaluates risk factors associated with COVID-19, severe COVID-19, and SARS-CoV-2 infection in adults participating in the placebo groups of 4 phase 3 COVID-19 vaccine efficacy trials.

## Introduction

SARS-CoV-2 infection and COVID-19 remain a significant global health challenge.^1,2^ Despite the development of safe and effective vaccines, globally, billions of people remain unvaccinated. Greater understanding of risk factors for infection and severe disease can guide future vaccine uptake prioritization strategies and therapeutic allocation policies.

Prospective studies have demonstrated that higher risk of SARS-CoV-2 infection is associated with demographic and behavioral cofactors.^3,4,5,6,7,8,9,10,11,12,13,14,15,16,17,18^ In contrast, the associations of underlying medical conditions with infection acquisition are less well-defined, although retrospective reports have reported diabetes and obesity were associated with increased risk.^7,19,20,21,22,23,24^ Prospective studies have identified both demographic characteristics and comorbidities as risk factors associated with severe COVID-19.^25,26,27,28,29,30,31^

Much of the data defining risk for either acquisition of SARS-CoV-2 or severe COVID-19 come from studies that relied on surveys, registries, or electronic medical records. These data often lack a harmonized definition of outcomes, and none used active surveillance. Moreover, high-quality data regarding risk factors for broadly symptomatic COVID-19 are sparse, despite clear, long-term impacts of disease.^32,33^

The COVID-19 Prevention Network (CoVPN) was formed by the US National Institutes of Health to conduct phase 3 vaccine clinical trials.^34^ Four randomized, controlled, efficacy trials were conducted with harmonized protocols beginning in 2020.^35,36,37,38^ The placebo recipients from these trials afford a large, diverse, multinational cohort. Using these detailed prospective data, and controlling for trial, region, and time in the pandemic, we sought to identify independent risk factors associated with COVID-19, severe COVID-19, any SARS-CoV-2 infection, and subclinical SARS-CoV-2 infection.

## Methods

### Study Design and Setting

We performed a secondary cross-protocol analysis of participant-level data from the blinded phase of 4 randomized, placebo-controlled, phase 3 COVID-19 vaccine efficacy trials. Local or central institutional review board and ethics committee approvals were obtained by each site participating in the 4 trials.^35,36,37,38^ All participants provided written informed consent to participate in the trials.

**Figure 1.**
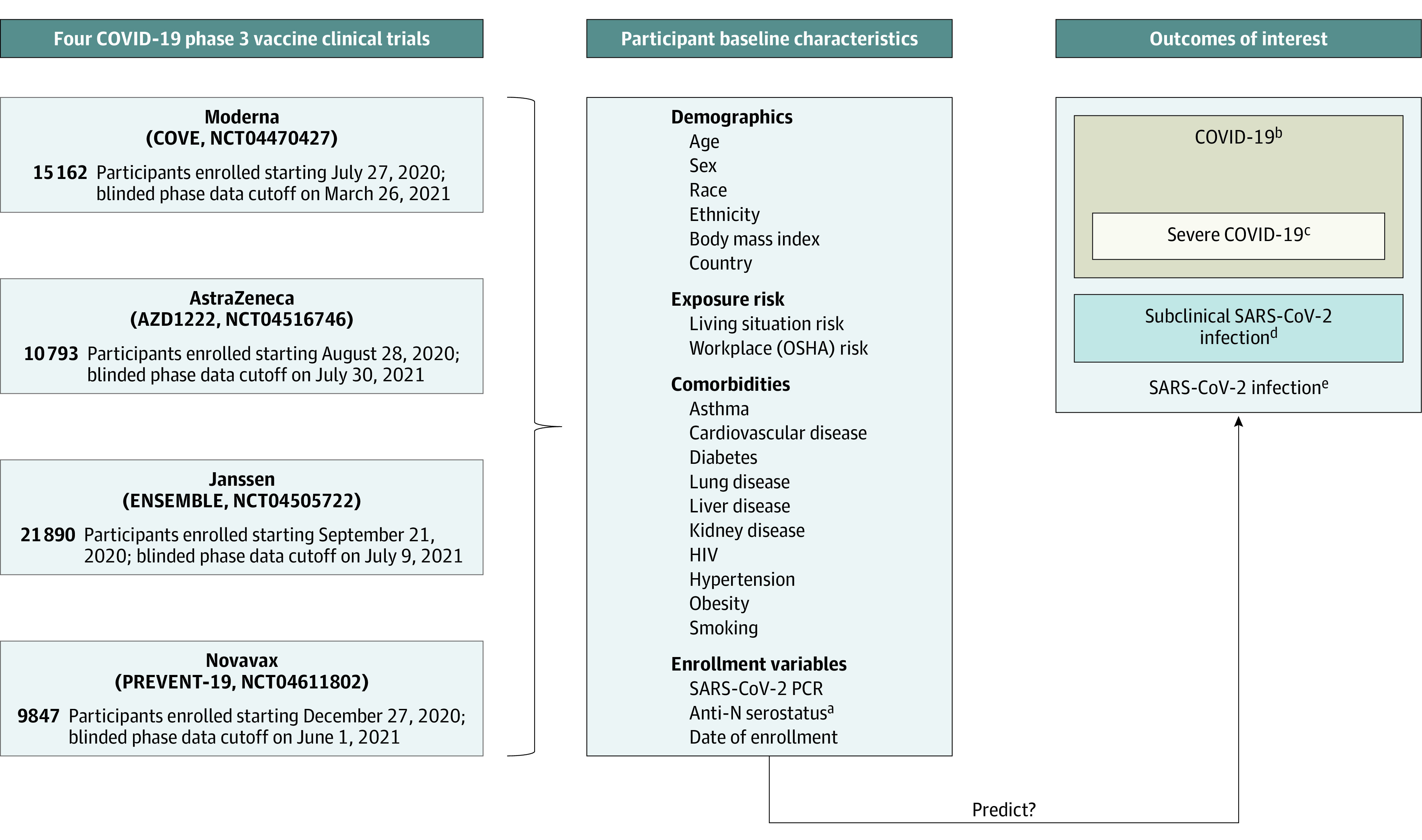
Study Cohort Composition ^a^Anti-N: Binding antibodies against SARS-CoV-2 nucleocapsid protein. ^b^Determined by a harmonized definition across studies with minor differences (eTable 1 in Supplement 1). ^c^Defined per Centers for Disease Control and Prevention criteria (see eTable 1 in Supplement 1). ^d^Detection of SARS-CoV-2 on reverse transcription–polymerase chain reaction testing and/or anti-N seroconversion (baseline seronegative to seropositive) detected at designated study visits among participants who never met the definition for COVID-19. Limited to participants in per-protocol analysis. ^e^Meeting the end point of COVID-19 or meeting the subclinical SARS-CoV-2 infection end point. Limited to participants in per-protocol analysis.

We assessed data from participants in the placebo groups of the COVE/CoVPN3001 (ClinicalTrials.gov identifier: NCT04470427; mRNA1273, Moderna), AZD1222 (ChAdOx1 nCoV-19/CoVPN3002; ClinicalTrials.gov identifier: NCT04516746; AZD1222, AstraZeneca), ENSEMBLE /CoVPN3003 (ClinicalTrials.gov identifier: NCT04505722; Ad26.COV2.S, Janssen), and PREVENT-19/CoVPN3004 (ClinicalTrials.gov identifier: NCT04611802; NVX-CoV2373, Novavax) trials (Figure 1), hereafter, referred to as the Moderna, AstraZeneca, Janssen, and Novavax cohorts. Individual study protocols describing study design have been previously published.^35,36,37,38^

Major eligibility criteria were age at least 18 years, in stable health, and at risk for SARS-CoV-2 infection.^39^ This analysis constitutes data accrued through the blinded, precrossover phases of the trials from placebo recipients who received at least the first study injection.

Trial start dates and blinded precrossover phase cutoff dates are presented in Figure 1. Participants were enrolled from 8 countries, with enrollment beginning in July 2020 and the last blinded phase data cutoff in July 2021.

### Study End Points

Study end points diagnosed at least 1 day after the first placebo administration were included and censored at the earliest date of drop out, unblinding, crossover, receipt of outside vaccination, or blinded phase data cutoff. The primary end point was time to COVID-19, defined with minor differences across studies as positive SARS-CoV-2 reverse transcriptase–polymerase chain reaction (RT-PCR) test result and systemic and/or respiratory symptoms (eTable 1 in Supplement 1).^39^ The severe COVID-19 end point was defined per Centers for Disease Control and Prevention criteria, including shortness of breath at rest or respiratory distress, respiratory rate of at least 30 breaths per minute, heart rate of at least 125 beats per minute, oxygen saturation 93% or less on room air, respiratory or multiorgan failure, intensive care unit admission, or death (eTable 1 in Supplement 1).

The subclinical SARS-CoV-2 infection end point was defined as having positive SARS-CoV-2 RT-PCR test result or antinucleocapsid protein (anti-N) test result, but not meeting the definition for the primary (symptomatic) COVID-19 end point among participants whose test results were negative for SARS-CoV-2 at baseline. The testing schedule is shown in eTable 2 in Supplement 1.

The any SARS-CoV-2 infection end point was defined as meeting the primary COVID-19 end point or meeting the subclinical SARS-CoV-2 infection end point among participants whose test results were negative for SARS-CoV-2 at baseline. Each of the 4 trials included anti-N testing at designated visits; the Moderna and Novavax cohorts also included SARS-CoV-2 RT-PCR testing regardless of symptoms at designated visits (eTable 2 in Supplement 1). Additional analyses were performed to identify variables associated with COVID-19 rates among participants meeting the any SARS-CoV-2 infection end point and to identify variables associated with severe COVID-19 rates among participants meeting the COVID-19 end point.

### Baseline Characteristics

We evaluated 23 pre-enrollment variables as potential risk factors for study outcomes. These variables included demographic characteristics: age (≥65 vs 18-64 years), sex assigned at birth, race, ethnicity, body mass index (BMI; calculated as weight in kilograms divided by height in meters squared), and region (SARS-CoV-2 epidemiological characteristics in South America, North America, or South Africa, where trials were conducted); comorbid conditions: asthma, cardiovascular disease, hypertension, diabetes, smoking, obesity (defined as BMI ≥30), lung disease, liver disease, kidney disease, HIV, and number of comorbidities; and SARS-CoV-2 exposure risk or history: Occupational Safety and Health Administration risk category (high, medium, and low), living situation risk score (very high, high, medium, and low), evidence of previous SARS-CoV-2 infection (defined as positive SARS-CoV-2 PCR test result or anti-N serostatus) at screening or enrollment, and calendar date of enrollment. Race and ethnicity were collected by self report and categorized as American Indian or Alaska Native, Asian, Black or African American, White, other, and multiple. Indigenous people from South America were classified together with the American Indian or Alaska Native US and Mexico demographic according to the US Food and Drug definition (ie, a person having origins in any of the original peoples of North and South America [including Central America] and who maintains tribal affiliation or community attachment). Participants also had the option to select other race without specifying. Ethnicity was categorized as Hispanic or Latino or not Hispanic or Latino. Race and ethnicity were included in analysis to describe participant demographic characteristics. Definitions and derivations of these variables are provided in the eMethods and eTable 3 in Supplement 1.

### Statistical Analysis

The COVID-19 and severe COVID-19 end points were evaluated with the full cohort of participants who received the first placebo administration. The any SARS-CoV-2 infection and subclinical SARS-CoV-2 infection end points analyses were restricted to the per-protocol population of participants with negative SARS-CoV-2 test results at baseline who received all planned placebo injections. Instantaneous hazard estimates over calendar time were obtained by smoothing the increments of the Nelson-Aalen estimator for the cumulative hazard function and plotted with global epidemiologic trends using information from the World Health Organization (WHO)^40^ and GISAID.^41^ Missing data were imputed by the median for continuous variables and by the most frequent category for categorical variables. Univariate and multivariate analyses generated adjusted hazard ratios (aHRs) adjusted for study and region as stratification variables (reflecting SARS-CoV-2 epidemiological characteristics in North America, South America, and South Africa) to account for potentially different baseline hazard functions across trials and regions. Survival random forest, as implemented in the randomForestSRC package in R statistical software version 4.1.0 (R Project for Statistical Computing), was used to rank the relative importance of covariates, without imposing any parametric assumptions on the association between the risk factors and the study outcomes, while accounting for potential correlations among the risk factors.^42^ Univariate and multivariate Cox proportional hazard regression models, with and without 2-way interaction terms for age (18-64 vs ≥65 years) or ethnicity and each of the other covariates, were used to estimate the associations of baseline covariates using both calendar and study time scales. Given the similarity of the results, only calendar time models are presented. The Cox proportional hazard assumption was evaluated for each risk factor considered in the models.^43^ The model assumption results for the primary COVID-19 end point are shown in eFigure 1 in Supplement 1. False-discovery rate–based *P* value adjustment was applied within each end point for all univariate analyses. The final multivariate Cox models with and without interactions were chosen by the Akaike information criterion in a stepwise algorithm. The final multivariate Cox model with 2-way interactions kept interaction terms with *P* < .01 for reliability of interpretation. Consequently, not all variables that were significant in the univariate analysis were included in the multivariate model. *P* < .01 was considered statistically significant. All analyses were performed using R software version 4.1.0. Data were analyzed from April 2022 to February 2023.

## Results

### Demographics

Across the 4 studies, 57 692 participants were randomized and received placebo injections. Median (range) age was 51 (18-95) years; 31 058 (53.8%) were male, and 11 720 participants (20.3%) were aged 65 years or older (Table). The Novavax study enrolled the youngest population, with 12.6% of placebo participants aged 65 years or older.

**Table.** Demographics, Comorbid Conditions, and Exposure Risk for Placebo Participants by Trial and Pooled

Characteristic	Study participants, No. (%)				
	Moderna (n = 15 162)	AstraZeneca (n = 10 793)	Janssen (n = 21 890)	Novavax (n = 9847)	Total (n = 57 692)
Age, y					
Median (range)	52 (18-95)	51 (18-93)	52 (18-94)	47 (18-90)	51 (18-95)
≥65	3751 (24.7)	2436 (22.6)	4298 (19.6)	1235 (12.5)	11 720 (20.3)
18-64	11 411 (75.3)	8357 (77.4)	17 592 (80.4)	8612 (87.5)	45 972 (79.7)
BMI					
Median (range)	28.1 (10.3-72.7)	27.7 (14.9-71.7)	27 (13.3-82.6)	28 (10.5-71.7)	27.5 (10.3-82.6)
Missing	85 (0.6)	113 (1.0)	18 (0.1)	39 (0.4)	255 (0.4)
Sex at birth					
Female	7106 (46.9)	4789 (44.4)	9907 (45.3)	4828 (49.0)	26 630 (46.2)
Male	8056 (53.1)	6004 (55.6)	11 979 (54.7)	5019 (51.0)	31 058 (53.8)
Intersex^a^	NA	NA	4 (<0.1)	NA	4 (<0.1)
Country					
Argentina	NA	NA	1498 (6.8)	NA	1498 (2.6)
Brazil	NA	NA	3635 (16.6)	NA	3635 (6.3)
Chile	NA	729 (6.8)	570 (2.6)	NA	1299 (2.3)
Colombia	NA	NA	2123 (9.7)	NA	2123 (3.7)
Mexico	NA	NA	241 (1.1)	588 (6.0)	829 (1.4)
Peru	NA	491 (4.5)	885 (4.0)	NA	1376 (2.4)
South Africa	NA	NA	3289 (15.0)	NA	3289 (5.7)
United States	15 162 (100.0)	9573 (88.7)	9649 (44.1)	9259 (94.0)	43 643 (75.6)
Race^b^					
American Indian or Alaska Native	121 (0.8)	428 (4.0)	2060 (9.4)	661 (6.7)	3270 (5.7)
Asian	739 (4.9)	482 (4.5)	686 (3.1)	416 (4.2)	2323 (4.0)
Black or African American	1531 (10.1)	892 (8.3)	4262 (19.5)	1164 (11.8)	7849 (13.6)
White	11 998 (79.1)	8523 (79.0)	12 843 (58.7)	7381 (75.0)	40 745 (70.6)
Other	326 (2.2)	21 (0.2)	47 (0.2)	12 (0.1)	406 (0.7)
Multiple	318 (2.1)	257 (2.4)	1248 (5.7)	159 (1.6)	1982 (3.4)
Missing	129 (0.9)	190 (1.8)	744 (3.4)	54 (0.5)	1117 (1.9)
Ethnicity					
Hispanic or Latino	3108 (20.5)	2451 (22.7)	9964 (45.5)	2155 (21.9)	17 678 (30.6)
Not Hispanic or Latino	11 918 (78.6)	8202 (76.0)	11 367 (51.9)	7669 (77.9)	39 156 (67.9)
Missing	136 (0.9)	140 (1.3)	559 (2.6)	23 (0.2)	858 (1.5)
Comorbid conditions					
Asthma	1429 (9.4)	1149 (10.6)	1073 (4.9)	942 (9.6)	4593 (8.0)
Cardiovascular disease	4728 (31.2)	3079 (28.5)	5797 (26.5)	2392 (24.3)	15 996 (27.7)
Diabetes	1567 (10.3)	1096 (10.2)	1968 (9.0)	998 (10.1)	5629 (9.8)
History of smoking	274 (1.8)	2059 (19.1)	404 (1.8)	3103 (31.5)	5840 (10.1)
HIV	96 (0.6)	171 (1.6)	621 (2.8)	65 (0.7)	953 (1.7)
Hypertension	4517 (29.8)	2924 (27.1)	5544 (25.3)	2325 (23.6)	15 310 (26.5)
Kidney disease	82 (0.5)	62 (0.6)	123 (0.6)	64 (0.6)	331 (0.6)
Liver disease	107 (0.7)	209 (1.9)	180 (0.8)	74 (0.8)	570 (1.0)
Lung disease	870 (5.7)	1347 (12.5)	1330 (6.1)	1446 (14.7)	4993 (8.7)
Obesity	5871 (38.7)	3883 (36.0)	6326 (28.9)	3799 (38.6)	19 879 (34.5)
≥2 Comorbid conditions	5613 (37.0)	4559 (42.2)	6903 (31.5)	4089 (41.5)	21 164 (36.7)
≥3 Comorbid conditions	3284 (21.7)	2671 (24.7)	3813 (17.4)	2463 (25.0)	12 231 (21.2)
≥4 Comorbid conditions	1198 (7.9)	1198 (7.9)	1297 (5.9)	1267 (12.9)	4960 (8.6)
Living condition^c^					
Very high risk	441 (2.9)	1560 (14.5)	1470 (6.7)	155 (1.6)	3626 (6.3)
High risk	1302 (8.6)	1665 (15.4)	4083 (18.7)	398 (4.0)	7448 (12.9)
Medium risk	10 942 (72.2)	1976 (18.3)	6367 (29.1)	1297 (13.2)	20 582 (35.7)
Low risk	2477 (16.3)	5592 (51.8)	9970 (45.5)	7997 (81.2)	26 036 (45.1)
Workplace risk of exposure to SARS-CoV-2^d^					
High	5314 (35.1)	2975 (27.6)	660 (3.0)	886 (9.0)	9835 (17.0)
Medium	3219 (21.2)	4485 (41.6)	276 (1.3)	3209 (32.6)	11 189 (19.4)
Low	0	3208 (29.7)	20 927 (95.6)	5752 (58.4)	29 887 (51.8)
Missing or other	6629 (43.7)^e^	125 (1.2)	27 (0.1)	0	6781 (11.8)
Baseline SARS-CoV-2 status					
Positive RT-PCR test result	95 (0.6)	7 (0.1)	127 (0.6)	107 (1.1)	336 (0.6)
Positive serostatus	303 (2.0)	293 (2.7)	2103 (9.6)	623 (6.3)	3322 (5.8)
Overall positive status^f^	337 (2.2)	300 (2.8)	2208 (10.1)	691 (7.0)	3536 (6.1)

Abbreviations: BMI, body mass index (calculated as weight in kilograms divided by height in meters squared); RT-PCR, reverse transcriptase–polymerase chain reaction.

^a^ Intersex individuals excluded in subsequent analyses.

^b^ The American Indian or Alaska Native category is defined across all clinical sites. Indigenous people from South America were classified together with the American Indian or Alaska Native US and Mexico demographic according to the US Food and Drug Administration definition (ie, American Indian or Alaska Native: a person having origins in any of the original peoples of North and South America [including Central America] and who maintains tribal affiliation or community attachment). In this analysis, the Moderna, AstraZeneca, Janssen and Novavax trials included 121, 100, 114, and 661 participants, respectively, who identified as American Indian or Alaska Native from North America. Respondents who checked multiple options were categorized as multiple races. The other category includes respondents who checked that their race was not 1 or more of the categories listed above.

^c^ Living condition encompasses housing type and household size, detailed derivation provided in the eMethods in Supplement 1.

^d^ Detailed derivation of exposure risk based on Occupational Safety and Health Administration categories is provided in the eMethods in Supplement 1. A total of 704 participants from the AstraZeneca trial identified as very high risk were combined with the high-risk category.

^e^ In the Moderna trial, 26.5% of participants reported other in the workspace questionnaire; 17.2% responses were missing.

^f^ Overall positive SARS-CoV-2 status was defined as having a positive RT-PCR test result or a positive serostatus.

Overall, 3270 participants (5.7%) were American Indian or Alaska Native, 7849 participants (13.6%) were Black or African American, 17 678 participants (30.6%) were Hispanic or Latino, and 40 745 participants (70.6%) were White. Most participants were enrolled in the US (43 643 participants [75.6%]), followed by Brazil (3635 participants [6.3%]), South Africa (3289 participants [5.7%]), Colombia (2123 participants [3.7%]), Argentina (1498 participants [2.6%]), Peru (1376 participants [2.4%]), Chile (1299 participants [2.3%]), and Mexico (829 participants [1.4%]). Demographic characteristics were largely consistent across the 4 trials; the Janssen study contributed the most diverse population racially, ethnically, and geographically (Table).

### Clinical Characteristics at Enrollment

Comorbid conditions among placebo recipients were distributed similarly across trials and included cardiovascular disease (15 996 participants [27.7%]), diabetes (5629 participants [9.8%]), kidney disease (331 participants [0.6%]), liver disease (570 participants [1.0%]), lung disease (4993 participants [8.7%]), obesity (19 879 participants [34.5%]), HIV (953 participants [1.7%]), history of smoking (5840 participants [10.1%]), and hypertension (15 310 participants [26.5%]). Across trials, 21 164 participants (36.7%) had 2 or more comorbidities, 12 231 participants (21.2%) had 3 or more comorbidities, and 4960 participants (8.6%) had 4 or more comorbidities. Most participants had negative results on baseline anti-N antibody and SARS-CoV-2 RT-PCR testing (54 156 participants [93.9%]).

### Exposure Risk

A low-risk living condition was most common (26 036 participants [45.1%]), followed by medium (20 582 participants [35.7%]), high (7448 participants [12.9%]), and very high (3626 participants [6.3%]). Living condition risk varied by study, reflecting regional differences of the individual trial populations. A lower workplace exposure risk was most common (29 887 participants [51.8%]), followed by medium (11 189 participants [19.4%]) and high (9131 participants [15.8%]) workplace exposure risk. Participants in the Janssen trial had the lowest workplace exposure risks.

### Incidence Rates by Study Outcome

**Figure 2.**
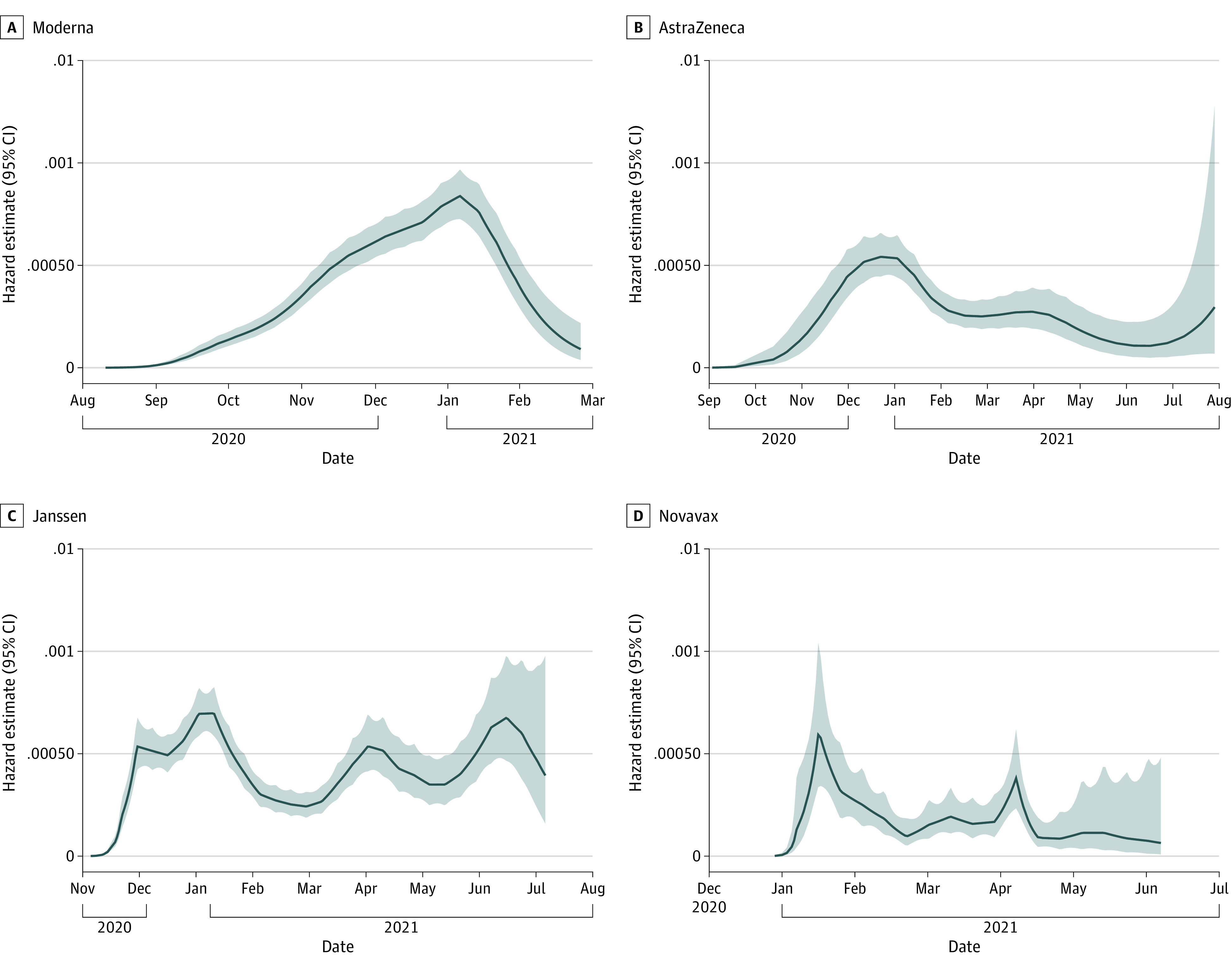
Smoothed Hazard Estimates of COVID-19 in Trial Placebo Participants Plotted by Calendar Time Smoothed hazard estimates are plotted over calendar time for the COVID-19 endpoint in each trial. Corresponding epidemiological trends within the countries contributing data to the trials, including number of cases and emergence of viral variants, are presented in eFigure 2 in Supplement 1.

Median (range) blinded precrossover follow-up time was 3.8 (0-11.1) months. Across the 4 trials, 2559 placebo recipients developed COVID-19, with an incidence rate of 13.9% (95% CI, 13.3%-14.4%); 367 placebo recipients developed severe COVID-19, with an incidence rate of 2.0% (95% CI, 1.8%-2.2%) (eTable 4 in Supplement 1). In the per-protocol cohort, 3774 placebo recipients developed any SARS-CoV-2 infection, with an incidence rate of 24.3% (95% CI, 23.5%-25.1%); 1612 placebo recipients developed subclinical infection, with an incidence rate of 10.3% (95% CI, 9.8%-10.9%). We plotted the smoothed hazard estimates over calendar time for COVID-19 in each trial (Figure 2; eFigure 2 in Supplement 1). To put our data into context, epidemiological trends for the regions of the trials are presented using information from the WHO^40^ and GISAID.^41^ Smoothed hazard peaks largely aligned with case trends in the countries contributing data to each study. The trials enrolled primarily during pandemic waves with the ancestral and Alpha variants. However, trials also included participants with infections with Beta, Delta, Epsilon, Gamma, Mu, and Lambda variants (Figure 2; eFigure 2 and eTable 5 in Supplement 1).

### Variables Associated with Study Outcomes

**Figure 3.**
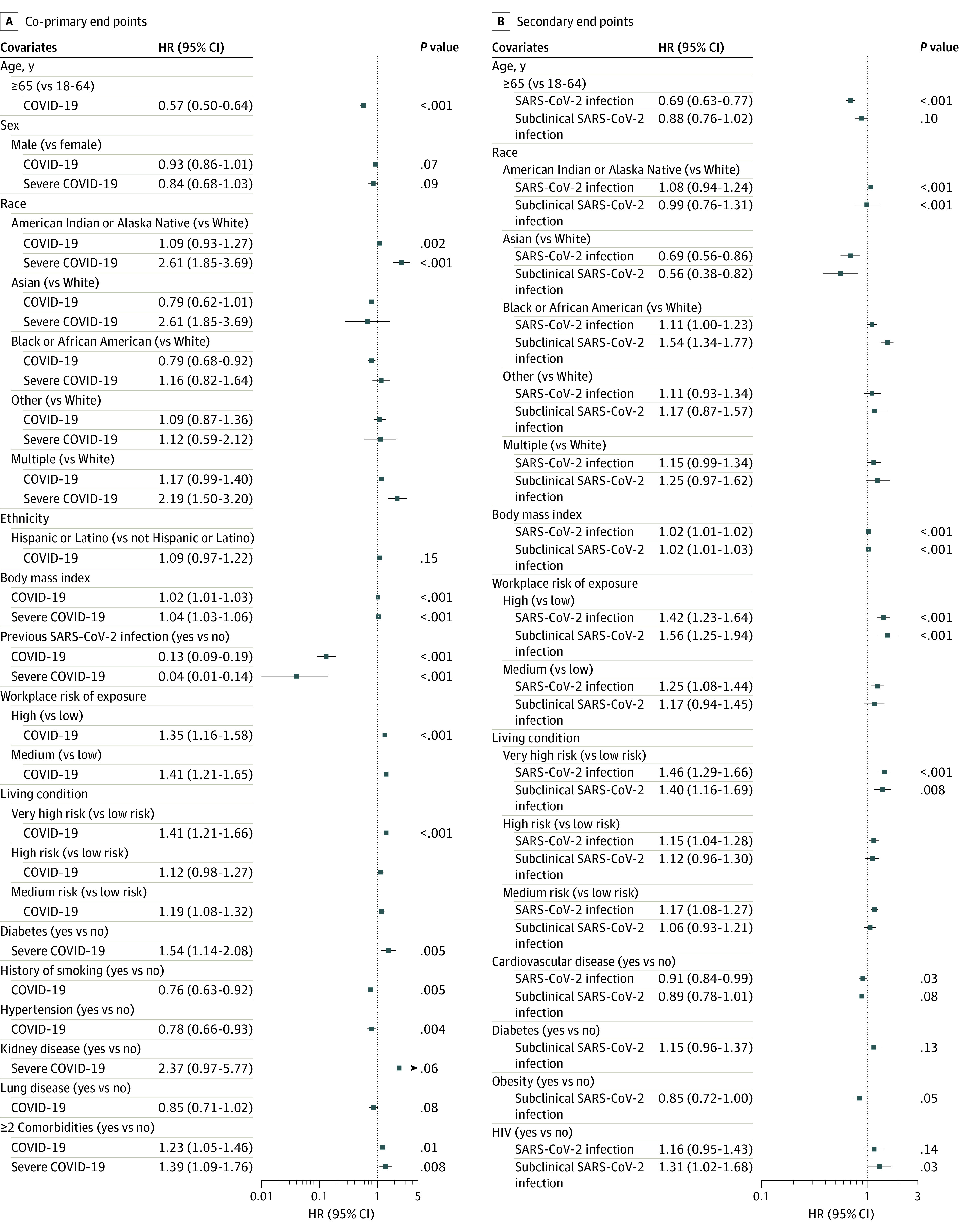
Multivariate Cox Proportional Hazard Regression Models for Each Study End Point All models adjusted for study (Moderna, AstraZeneca, Janssen, Novavax), Region (South America, North America, South Africa) and calendar time to account for potentially different baseline hazard functions across studies, regions, and time. *P* = .01 was considered statistically significant.

Univariate Cox regression model results are included in eFigure 3 and eFigure 4 in Supplement 1. In the multivariate Cox regression models, covariates associated with an increased rate of COVID-19 included workplace exposure risk (high vs low risk: aHR, 1.35 [95% CI, 1.16-1.58]; medium vs low risk: aHR, 1.41 [95% CI, 1.21-1.65]; overall *P* < .001), living condition risk (very high vs low risk: aHR, 1.41 [95% CI, 1.21-1.66]; medium vs low risk: aHR, 1.19 [95% CI, 1.08-1.32]; overall *P* < .001), and BMI (aHR per 1-unit increase, 1.02 [95% CI, 1.01-1.03]; *P* < .001) (Figure 3). Covariates associated with a decreased rate of COVID-19 included age 65 years or older (aHR vs <65 years, 0.57 [95% CI, 0.50-0.64]; *P* < .001), Black or African American race (aHR vs White race, 0.79 [95% CI, 0.68-0.92]; overall *P* = .002), evidence of previous SARS-CoV-2 infection at enrollment (aHR, 0.13 [95% CI, 0.09-0.19]; *P* < .001), hypertension (aHR, 0.78 [95% CI, 0.66-0.93]; *P* = .004), and history of smoking (aHR, 0.76 [95% CI, 0.63-0.92]; *P* = .005).

We then considered these covariates for the severe COVID-19 end point. Covariates associated with an increased rate of severe COVID-19 included American Indian or Alaska Native race (aHR vs White race, 2.61 [95% CI, 1.85-3.69]) and multiple races (aHR vs White race, 2.19 [95% CI, 1.50-3.20]; overall *P* < .001), higher BMI (aHR per 1-unit increase, 1.04 [95% CI, 1.03-1.06]; *P* < .001), diabetes (aHR, 1.54 [95% CI, 1.14-2.08]; *P* = .005), and having at least 2 comorbidities (aHR vs none, 1.39 [95% CI, 1.09-1.76]; *P* = .008). Previous SARS-CoV-2 infection at enrollment was associated with a decreased rate of severe COVID-19 (aHR, 0.04 [95% CI, 0.01-0.14]; *P* < .001).

In an analysis limited to participants meeting the COVID-19 end point, an increased rate of severe COVID-19 was associated with age at least 65 years (aHR vs <65 years, 1.75 [95% CI, 1.32-2.31]; *P* < .001), American Indian or Alaska Native race (aHR vs White race, 1.98 [95% CI, 1.38-2.83]), Black or African American race (aHR vs White race, 1.49 [95% CI, 1.03-2.14]), multiple races (aHR vs White race, 1.81 [95% CI, 1.21-2.69]; overall *P* for race = .001), BMI (aHR per 1-unit increase, 1.03 [95% CI, 1.01-1.04]; *P* = .001), and diabetes (aHR, 1.85 [95% CI, 1.37-2.49]; *P* < .001) (eFigure 5 and eFigure 6 in Supplement 1).

We next assessed covariates associated with rates of subclinical SARS-CoV-2 infection among participants in the per-protocol cohort. Covariates associated with an increased rate of subclinical SARS-CoV-2 infection included Black or African American race (aHR vs White race, 1.54 [95% CI, 1.34-1.77]; overall *P* < .001); higher BMI (HR per 1-unit increase, 1.02 [95% CI, 1.01-1.03]; *P* < .001), high workplace exposure risk (aHR vs low risk, 1.56 [95% CI, 1.25-1.94]; overall *P* < .001), and very high–risk living situation (aHR vs low risk, 1.40 [95% CI, 1.16-1.69]; *P* = .008). Asian race was associated with a lower rate of subclinical SARS-CoV-2 infection compared with White race (aHR, 0.56 [95% CI, 0.38-0.82]; *P* < .001).

We next analyzed the data across all participants in the per-protocol cohort meeting the any SARS-CoV-2 infection end point (COVID-19 or subclinical SARS-CoV-2 infection). Covariates associated with an increased rate of any SARS-CoV-2 infection included higher BMI (HR per 1-unit increase, 1.02 [95% CI, 1.01-1.02]; *P* < .001); workplace exposure risk (high vs low risk: aHR, 1.42 [95% CI, 1.23-1.64]; medium vs low risk: aHR, 1.25 [95% CI, 1.08-1.44]; overall *P* < .001); living situation risk (very high vs low risk: aHR, 1.46 [95% CI, 1.29-1.66]; high vs low risk: aHR, 1.15 [95% CI, 1.04-1.28]; medium vs low risk: aHR, 1.17 [95% CI, 1.08-1.27]; overall *P* < .001), and Black or African American race (aHR vs White race, 1.11 [95% CI, 1.00-1.23]; *P* < .001).

However, among participants meeting the any SARS-CoV-2 infection definition, race was significantly associated with a decreased rate of developing COVID-19 (American Indian or Alaska Native vs White race: aHR, 0.63 [95% CI, 0.54-0.75]; Black or African American vs White race: aHR, 0.56 [95% CI, 0.47-0.67]; multiracial vs White race: aHR, 0.71 [95% CI, 0.59-0.88]; overall *P* < .001) (eFigure 7 and eFigure 8 in Supplement 1).

**Figure 4.**
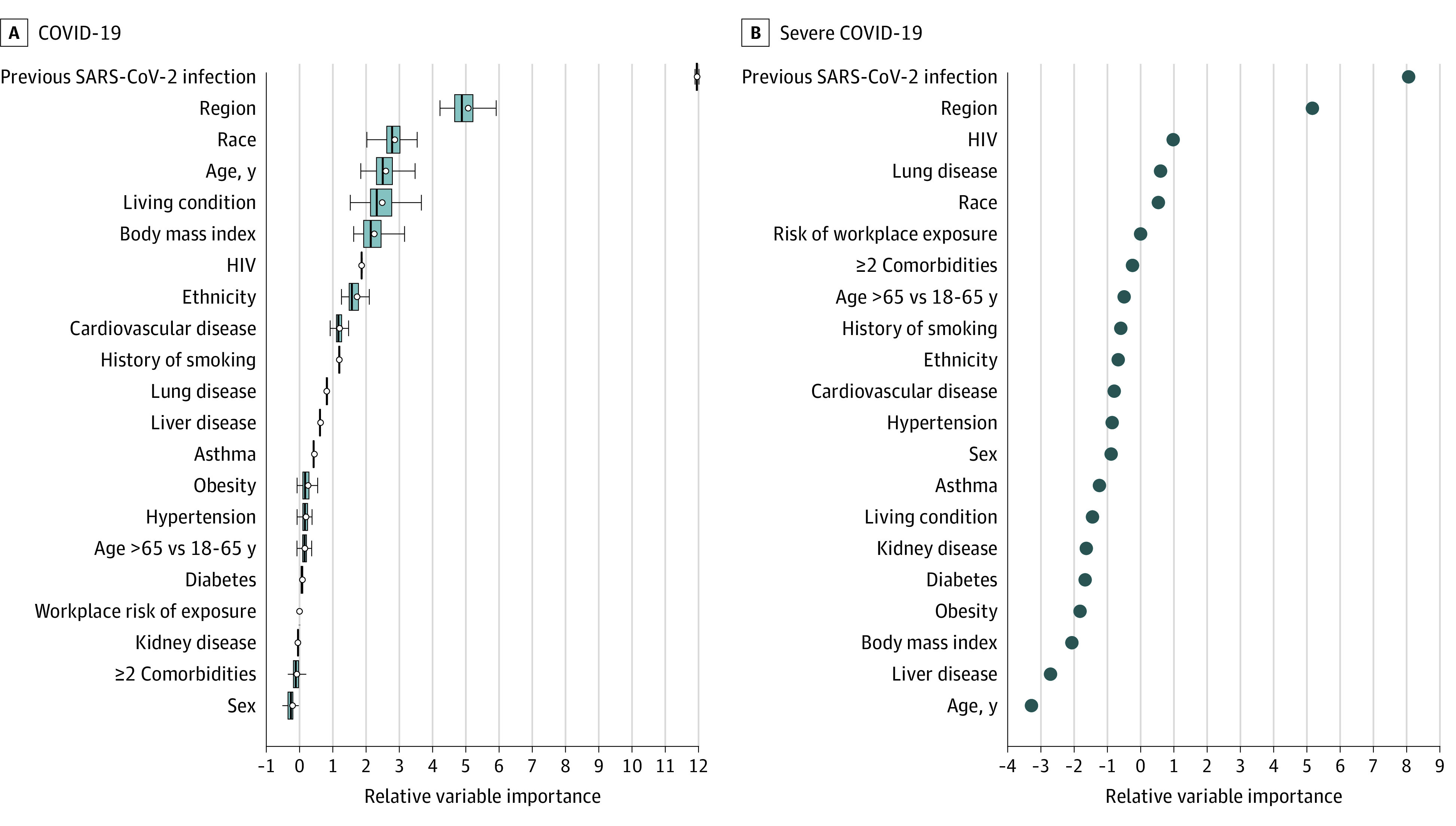
Survival Random Forest Plot: Ranking of Baseline Covariates by Strength of Association with the Coprimary Study End Points of COVID-19 and Severe COVID-19 Survival random forest was used to rank variables associated with rates of study outcomes. This analysis included both the covariates and stratification variables considered in the Cox models. Age is presented as both a continuous variable in years and stratified as 18 to 64 years vs 65 years or older. The default splitting rule (log-rank) and the default number of covariates randomly selected (square root of the total number of covariates) for each split of 1000 trees were used. The y-axis lists the variables that were included in the construction of the survival random forest. The x-axis ranks the strength of association, where variables to the left of the 0 are considered of negligible importance and variables to the right of 0 demonstrated increasing strength of association with the study outcomes. To calculate the relative variable importance of each variable, values of the given variable that were not used in the construction of a specific survival tree in the ensemble were permuted and the resulting estimation error was compared to that obtained without the permutation. The difference between the 2 estimation errors was then aggregated over all trees in the ensemble as the variable importance measure for the given variable. A subsampling approach was used to estimate the variance of the variable importance and for constructing confidence intervals. A, Ranking of variables associated with the COVID-19 end point, with circles indicating variable importance estimates. The midlines indicate medians; boxes, IQRs of estimates based on the subsampling approach. The whiskers extend to the most extreme estimates that are no more than 1.5 times the width of the box or if no estimate meets this criterion, to the estimate extremes. B, Ranking of variables associated with the severe COVID-19 end point. For this end point, all variables had a relatively small number for cases, thereby prohibiting confidence interval estimates.

The relative strength of association of covariates with each end point was assessed using a variable importance ranking analysis. Consistent with the Cox regression models, evidence of previous SARS-CoV-2 infection at enrollment, regional differences in SARS-CoV-2 epidemiological characteristics (eFigure 2 in Supplement 1), race, age, living condition, and BMI were top variables associated with COVID-19 rates; for severe COVID-19, evidence of previous SARS-CoV-2 infection at enrollment and region were top variables (Figure 4).

As age and ethnicity, 2 demographic characteristics of COVID-19 research focus, were associated with COVID-19 rates in the univariate analyses, we examined their interactions with other covariates and found significant interactions between age and workplace exposure risk and between evidence of previous SARS-CoV-2 infection and ethnicity. High workplace exposure was associated with an increased rate of COVID-19 among adults aged 18 to 64 years but a decreased rate among adults 65 years or older (eFigure 9 in Supplement 1). The inverse association of older age with COVID-19 rates lessened in strength as workplace exposure risk decreased (high exposure risk aHR, 0.32 [95% CI, 0.24-0.43]; medium exposure risk aHR, 0.59 [95% CI, 0.47-0.74]; low exposure risk aHR, 0.69 [95% CI, 0.58-0.81]; *P* for interaction < .001). Evidence of previous SARS-CoV-2 infection at enrollment demonstrated a stronger association with a decreased rate of COVID-19 among Hispanic or Latino (aHR, 0.08 [95% CI, 0.04-0.14]) compared with participants who were not Hispanic or Latino (aHR, 0.23 [95% CI, 0.15-0.36]; *P* for interaction = .004).

## Discussion

In this secondary cross protocol analysis of 4 randomized clinical trials, we present the largest, most diverse, global cohort with prospective active surveillance follow-up to our knowledge, enabling precise outcome ascertainment and identification of variables associated with COVID-19 rates. We noted high incidence of SARS-CoV-2 infection in approximately 4 months of follow-up, highlighting the remarkable infectivity of the virus and providing sufficient events for a robust risk assessment. Risk factors identified in this analysis can inform mitigation strategies for SARS-CoV-2 and viruses with comparable epidemiological characteristics. In addition, the characterization of these risk factors will increase efficiency in future analyses of this unique data set.

The sequential rollout of the 4 clinical trials allowed evaluation over different pandemic waves and diverse SARS-CoV-2 strains, affording generalizable results by capturing infections during periods of circulation of the ancestral strain, B.1.1.7 (Alpha variant), B.1.617.2 (Delta variant), B.1.351 (Beta variant), P.1 (Gamma variant), and C.37 (Lambda variant).^44^

In our analysis, hazard of COVID-19 was associated with workplace exposure risks and living condition risks and inversely associated with previous SARS-CoV-2 infection. Additionally, region had a strong association with hazard of COVID-19, which may reflect differences in study population and local mitigation practices, force of infection during the follow-up period, or the circulating variant.^14,15^ In this study, we confirm previous findings reporting associations of infection rates with exposure risk and baseline serostatus,^14,15^ and we offer additional insights, showing that prior infection and ongoing exposure risks at the individual, household, and regional level were associated not only with infection but also disease.

Some of our findings diverge from previously reported observations. For example, large prospective studies have found male sex was associated with SARS-CoV-2 acquisition and severe disease,^17,45^ which we did not find. Second, Black or African American race was associated with a higher rate of any SARS-CoV-2 infection and subclinical infection but a lower rate of COVID-19. This could reflect selection bias in our study population related to socioeconomic status or health care–seeking behavior of trial participants. Consistent with other reports, in the analysis of severe COVID-19 among participants with COVID-19, Black or African American race was associated with severe disease, as was American Indian or Alaska Native race. This striking finding for American Indian or Alaska Native participants has been previously reported^46,47,48^ with SARS-CoV-2 and other respiratory viruses^49,50^ and highlights the need to elucidate the mechanism behind the association.

Previous studies have demonstrated increased risk for severe COVID-19 among older individuals,^31^ which is consistent with other respiratory viruses, like influenza and respiratory syncytial virus.^51,52,53^ In our analysis, age had a strong inverse association with any SARS-CoV-2 infection and COVID-19, with the strongest inverse association among those with the highest workplace exposure risk. These findings likely reflect stricter adherence to social distancing and masking and lower social activity by older participants. This association was not seen with severe COVID-19, suggesting that a lower rate of infection due to behavioral adjustment obscured a truly higher rate of severe disease in older adults as was seen in our ad hoc analysis of severe disease rates restricted to participants with COVID-19.

In contrast to other reports that have shaped Centers for Disease Control and Prevention and WHO guidelines for prevention and treatment,^5,18,21^ in this study, specific comorbidities did not drive COVID-19 rates. Rather, we found inverse associations for COVID-19 with smoking and hypertension. Previous studies of the association of smoking with COVID-19 outcomes have yielded conflicting results, while hypertension emerged as a perceived risk factor for COVID-19 early in the pandemic, potentially influencing the behavior of study participants. As these associations did not persist when assessing COVID-19 risk only among participants with SARS-CoV-2 infection, their link with COVID-19 outcomes remains unclear. Regarding severe COVID-19, diabetes was the sole comorbidity associated with an increased rate; although we did find that having at least 2 comorbidities was associated with a 40% increased rate of severe disease. Overall, our data suggest preexisting immunity, demographics and exposure risks were the strongest variables associated with disease rates.

### Limitations

This study has some limitations. It is unclear how infection with specific variants (eg, Omicron) not captured in our study or vaccination would moderate identified risk factors. There were also differences in the severe COVID-19 definition for each trial, warranting additional study of the severe cases within this cohort. The relatively short follow-up time limits understanding of the durability of associations. Any infection and subclinical infection analyses were limited by lack of frequent molecular or anti-N testing, and emerging evidence of variable N antibody responses.^54^ Differentiation between symptomatic and subclinical infection depended on self-report, which may vary by demographics. In addition, although our analyses considered aggregated study- and region-specific associations of the risk factors, there could still be heterogeneity across the trials (eg, percentage of participants with smoking history) that was not adequately accounted for in the final multivariate models. Furthermore, ascertainment of comorbidities also relied on self-report, excepting obesity, which was measured by BMI. Variable access to health care among participants may have limited self-awareness of comorbid conditions. These considerations suggest a role for individualized risk stratification.

## Conclusions

In this secondary cross-protocol analysis, we conducted a detailed assessment of variables associated with rates of COVID-19, severe disease, any SARS-CoV-2 infection, and subclinical SARS-CoV-2 infection obtained systematically from the largest clinical trial cohort enrolled in diverse regions of the world with follow-up spanning multiple waves of the pandemic. Results offer generalizable and precise identification of risk factors and may inform future vaccination policy as SARS-CoV-2 becomes endemic to human populations. Results may also help identify populations at risk in the setting of potential future pandemics.

## Notes

Supplement 1.
eMethods.
**eFigure 1.** Cox Proportional Hazard Scaled Schoenfeld Residual Plots for Each Risk Factor **eFigure 2.** Smoothed Hazard Estimates of COVID-19 in Trial Placebo Participants Plotted by Calendar Time and Epidemiologic Trends **eFigure 3.** Univariate Cox Proportional Hazard Regression Models for the COVID-19 and Severe COVID-19 Coprimary End Points **eFigure 4.** Univariate Cox Proportional Hazard Regression Models for the Subclinical SARS-CoV-2 and Any SARS-CoV-2 Secondary End Points **eFigure 5.** Univariate Cox Proportional Hazard Regression Model for the Severe COVID-19 End Point Among Participants With COVID-19 **eFigure 6.** Multivariate Cox Proportional Hazard Regression Model for the Severe COVID-19 End Point Among Participants With COVID-19 **eFigure 7.** Univariate Cox Proportional Hazard Regression Model for the COVID-19 End Point Among Participants With SARS-CoV-2 **eFigure 8.** Multivariate Cox Proportional Hazard Regression Model for the COVID-19 End Point Among Participants With SARS-CoV-2 **eFigure 9.** Multivariate Cox Proportional Hazard Regression Models With 2-Way Interaction Terms for the COVID-19 End Point **eTable 1.** Study Case Definitions **eTable 2.** Schedule of Antinucleocapsid Protein Testing Across Protocols **eTable 3.** CDC Risk Factors for Severe Disease **eTable 4.** Study End Points Stratified by Trial **eTable 5.** Variant Counts From COVID-19 Cases

Supplement 2.
Members of the COVID-19 Prevention Network (CoVPN)

Supplement 3.
Data Sharing Statement

## References

[1] World Health Organization. Weekly operational update on COVID-19. Accessed June 6, 2023. https://www.who.int/emergencies/diseases/novel-coronavirus-2019/situation-reports

[2] Johns Hopkins Coronavirus Research Center. Accessed June 6, 2023. https://coronavirus.jhu.edu/

[3] Al Maskari Z, Al Blushi A, Khamis F, . Characteristics of healthcare workers infected with COVID-19: a cross-sectional observational study. Int J Infect Dis. 2021;102:32-36. doi:10.1016/j.ijid.2020.10.009 33039607 PMC7543901

[4] Kilpatrick RD, Sánchez-Soliño O, Alami NN, . Epidemiological Population Study of SARS-CoV-2 in Lake County, Illinois (CONTACT): methodology and baseline characteristics of a community-based surveillance study. Infect Dis Ther. 2022;11(2):899-911. doi:10.1007/s40121-022-00593-0 35107821 PMC8808268

[5] McCloskey JK, Ellis JL, Uratsu CS, . Accounting for social risk does not eliminate race/ethnic disparities in COVID-19 infection among insured adults: a cohort study. J Gen Intern Med. 2022;37(5):1183-1190. doi:10.1007/s11606-021-07261-y 35107716 PMC8809238

[6] Owusu M, Sylverken AA, Ankrah ST, . Epidemiological profile of SARS-CoV-2 among selected regions in Ghana: a cross-sectional retrospective study. PLoS One. 2020;15(12):e0243711. doi:10.1371/journal.pone.0243711 33301533 PMC7728229

[7] Rodriguez-Diaz CE, Guilamo-Ramos V, Mena L, . Risk for COVID-19 infection and death among Latinos in the United States: examining heterogeneity in transmission dynamics. Ann Epidemiol. 2020;52:46-53.e2. doi:10.1016/j.annepidem.2020.07.007 32711053 PMC7375962

[8] Schwartz KL, Achonu C, Buchan SA, . Epidemiology, clinical characteristics, household transmission, and lethality of severe acute respiratory syndrome coronavirus-2 infection among healthcare workers in Ontario, Canada. PLoS One. 2020;15(12):e0244477. doi:10.1371/journal.pone.0244477 33370384 PMC7769426

[9] Hall VJ, Foulkes S, Charlett A, ; SIREN Study Group. SARS-CoV-2 infection rates of antibody-positive compared with antibody-negative health-care workers in England: a large, multicentre, prospective cohort study (SIREN). Lancet. 2021;397(10283):1459-1469. doi:10.1016/S0140-6736(21)00675-9 33844963 PMC8040523

[10] Holt H, Talaei M, Greenig M, . Risk factors for developing COVID-19: a population-based longitudinal study (COVIDENCE UK). Thorax. 2022;77(9):900-912. doi:10.1136/thoraxjnl-2021-217487 34848555

[11] Iversen K, Kristensen JH, Hasselbalch RB, . Seroprevalence of SARS-CoV-2 antibodies and reduced risk of reinfection through 6 months: a Danish observational cohort study of 44 000 healthcare workers. Clin Microbiol Infect. 2022;28(5):710-717. doi:10.1016/j.cmi.2021.09.005 34543759 PMC8447554

[12] Krutikov M, Palmer T, Tut G, . Incidence of SARS-CoV-2 infection according to baseline antibody status in staff and residents of 100 long-term care facilities (VIVALDI): a prospective cohort study. Lancet Healthy Longev. 2021;2(6):e362-e370. doi:10.1016/S2666-7568(21)00093-3 34104901 PMC8175048

[13] Letizia AG, Ge Y, Vangeti S, . SARS-CoV-2 seropositivity and subsequent infection risk in healthy young adults: a prospective cohort study. Lancet Respir Med. 2021;9(7):712-720. doi:10.1016/S2213-2600(21)00158-2 33865504 PMC8049591

[14] Lin A, Vittinghoff E, Olgin J, . Predictors of incident SARS-CoV-2 infections in an international prospective cohort study. BMJ Open. 2021;11(9):e052025. doi:10.1136/bmjopen-2021-052025 34548363 PMC8457993

[15] Lumley SF, O’Donnell D, Stoesser NE, ; Oxford University Hospitals Staff Testing Group. Antibody status and incidence of SARS-CoV-2 infection in health care workers. N Engl J Med. 2021;384(6):533-540. doi:10.1056/NEJMoa2034545 33369366 PMC7781098

[16] Merino J, Joshi AD, Nguyen LH, . Diet quality and risk and severity of COVID-19: a prospective cohort study. Gut. 2021;70(11):2096-2104. doi:10.1136/gutjnl-2021-325353 34489306 PMC8500931

[17] Radon K, Bakuli A, Pütz P, ; KoCo19 study group. From first to second wave: follow-up of the prospective COVID-19 cohort (KoCo19) in Munich (Germany). BMC Infect Dis. 2021;21(1):925. doi:10.1186/s12879-021-06589-4 34493217 PMC8423599

[18] Wang G, Foney DM, DiBari J, . A prospective cohort study on the intersectionality of obesity, chronic disease, social factors, and incident risk of COVID-19 in US low-income minority middle-age mothers. Int J Obes (Lond). 2021;45(12):2577-2584. doi:10.1038/s41366-021-00943-x 34413468 PMC8374030

[19] Cordero-Franco HF, De La Garza-Salinas LH, Gomez-Garcia S, Moreno-Cuevas JE, Vargas-Villarreal J, González-Salazar F. Risk factors for SARS-CoV-2 infection, pneumonia, intubation, and death in Northeast Mexico. Front Public Health. 2021;9:645739. doi:10.3389/fpubh.2021.645739 34291023 PMC8287121

[20] de Lusignan S, Dorward J, Correa A, . Risk factors for SARS-CoV-2 among patients in the Oxford Royal College of General Practitioners Research and Surveillance Centre primary care network: a cross-sectional study. Lancet Infect Dis. 2020;20(9):1034-1042. doi:10.1016/S1473-3099(20)30371-6 32422204 PMC7228715

[21] Wander PL, Lowy E, Beste LA, . The incidence of diabetes among 2,777,768 veterans with and without recent SARS-CoV-2 infection. Diabetes Care. 2022;45(4):782-788. doi:10.2337/dc21-1686 35085391 PMC9016731

[22] Choi YJ, Park JY, Lee HS, . Effect of asthma and asthma medication on the prognosis of patients with COVID-19. Eur Respir J. 2021;57(3):2002226. doi:10.1183/13993003.02226-2020 32978309 PMC7518077

[23] Izquierdo JL, Almonacid C, González Y, . The impact of COVID-19 on patients with asthma. Eur Respir J. 2021;57(3):2003142. doi:10.1183/13993003.03142-2020 33154029 PMC7651839

[24] Shastri MD, Shukla SD, Chong WC, . Smoking and COVID-19: what we know so far. Respir Med. 2021;176:106237. doi:10.1016/j.rmed.2020.106237 33246296 PMC7674982

[25] Fresán U, Guevara M, Trobajo-Sanmartín C, Burgui C, Ezpeleta C, Castilla J. Hypertension and related comorbidities as potential risk factors for COVID-19 hospitalization and severity: a prospective population-based cohort study. J Clin Med. 2021;10(6):1194. doi:10.3390/jcm10061194 33809217 PMC8000595

[26] Galloway JB, Norton S, Barker RD, . A clinical risk score to identify patients with COVID-19 at high risk of critical care admission or death: An observational cohort study. J Infect. 2020;81(2):282-288. doi:10.1016/j.jinf.2020.05.064 32479771 PMC7258846

[27] Hamer M, Gale CR, Batty GD. Diabetes, glycaemic control, and risk of COVID-19 hospitalisation: population-based, prospective cohort study. Metabolism. 2020;112:154344. doi:10.1016/j.metabol.2020.154344 32835758 PMC7442562

[28] Kaeuffer C, Le Hyaric C, Fabacher T, ; Covid Alsace Study Group; COVID Alsace Study Group. Clinical characteristics and risk factors associated with severe COVID-19: prospective analysis of 1,045 hospitalised cases in North-Eastern France, March 2020. Euro Surveill. 2020;25(48):2000895. doi:10.2807/1560-7917.ES.2020.25.48.2000895 33272355 PMC7716399

[29] Kragholm K, Andersen MP, Gerds TA, . Association between male sex and outcomes of coronavirus disease 2019 (COVID-19)—a Danish nationwide, register-based study. Clin Infect Dis. 2021;73(11):e4025-e4030. doi:10.1093/cid/ciaa924 32634827 PMC7454435

[30] Lowe KE, Zein J, Hatipoglu U, Attaway A. Association of smoking and cumulative pack-year exposure with COVID-19 outcomes in the Cleveland Clinic COVID-19 registry. JAMA Intern Med. 2021;181(5):709-711. doi:10.1001/jamainternmed.2020.8360 33492361 PMC7835916

[31] Telle KE, Grøsland M, Helgeland J, Håberg SE. Factors associated with hospitalization, invasive mechanical ventilation treatment and death among all confirmed COVID-19 cases in Norway: prospective cohort study. Scand J Public Health. 2021;49(1):41-47. doi:10.1177/1403494820985172 33461404

[32] Carfì A, Bernabei R, Landi F; Gemelli Against COVID-19 Post-Acute Care Study Group. Persistent symptoms in patients after acute COVID-19. JAMA. 2020;324(6):603-605. doi:10.1001/jama.2020.12603 32644129 PMC7349096

[33] Xie Y, Xu E, Bowe B, Al-Aly Z. Long-term cardiovascular outcomes of COVID-19. Nat Med. 2022;28(3):583-590. doi:10.1038/s41591-022-01689-3 35132265 PMC8938267

[34] Corey L, Mascola JR, Fauci AS, Collins FS. A strategic approach to COVID-19 vaccine R&D. Science. 2020;368(6494):948-950. doi:10.1126/science.abc5312 32393526

[35] Baden LR, El Sahly HM, Essink B, ; COVE Study Group. Efficacy and Safety of the mRNA-1273 SARS-CoV-2 vaccine. N Engl J Med. 2021;384(5):403-416. doi:10.1056/NEJMoa2035389 33378609 PMC7787219

[36] Dunkle LM, Kotloff KL, Gay CL, ; 2019nCoV-301 Study Group. Efficacy and safety of NVX-CoV2373 in adults in the United States and Mexico. N Engl J Med. 2022;386(6):531-543. doi:10.1056/NEJMoa2116185 34910859 PMC8693692

[37] Falsey AR, Sobieszczyk ME, Hirsch I, ; AstraZeneca AZD1222 Clinical Study Group. Phase 3 safety and efficacy of AZD1222 (ChAdOx1 nCoV-19) COVID-19 vaccine. N Engl J Med. 2021;385(25):2348-2360. doi:10.1056/NEJMoa2105290 34587382 PMC8522798

[38] Sadoff J, Gray G, Vandebosch A, ; ENSEMBLE Study Group. Safety and efficacy of single-dose Ad26.COV2.S vaccine against COVID-19. N Engl J Med. 2021;384(23):2187-2201. doi:10.1056/NEJMoa2101544 33882225 PMC8220996

[39] Mena Lora AJ, Long JE, Huang Y, ; COVID-19 Prevention Network. Rapid development of an integrated network infrastructure to conduct phase 3 COVID-19 vaccine trials. JAMA Netw Open. 2023;6(1):e2251974. doi:10.1001/jamanetworkopen.2022.51974 36689221 PMC10546713

[40] World Health Organization. WHO Coronavirus (COVID-19) Dashboard. Accessed June 7, 2023. https://covid19.who.int/

[41] GISAID. Accessed June 7, 2023. https://gisaid.org/

[42] Ishwaran H, Lu M. Standard errors and confidence intervals for variable importance in random forest regression, classification, and survival. Stat Med. 2019;38(4):558-582. doi:10.1002/sim.7803 29869423 PMC6279615

[43] Grambsch PM, Therneau TM. Proportional Hazards Tests and Diagnostics Based on Weighted Residuals. Biometrika. 1994;81(3):515-526. doi:10.1093/biomet/81.3.515

[44] Bai Y, He Q, Yang J, . B.1.351 SARS-CoV-2 variant exhibits higher virulence but less viral shedding than that of the ancestral strain in young nonhuman primates. Microbiol Spectr. 2022;10(5):e0226322. doi:10.1128/spectrum.02263-22 36069561 PMC9603226

[45] Jassat W, Mudara C, Ozougwu L, ; DATCOV author group. Difference in mortality among individuals admitted to hospital with COVID-19 during the first and second waves in South Africa: a cohort study. Lancet Glob Health. 2021;9(9):e1216-e1225. doi:10.1016/S2214-109X(21)00289-8 34252381 PMC8270522

[46] Hatcher SM, Agnew-Brune C, Anderson M, . COVID-19 among American Indian and Alaska Native persons—23 states, January 31-July 3, 2020. MMWR Morb Mortal Wkly Rep. 2020;69(34):1166-1169. doi:10.15585/mmwr.mm6934e1 32853193 PMC7451969

[47] Dorn AV, Cooney RE, Sabin ML. COVID-19 exacerbating inequalities in the US. Lancet. 2020;395(10232):1243-1244. doi:10.1016/S0140-6736(20)30893-X 32305087 PMC7162639

[48] The COVID Tracking Project. COVID-19 is affecting Black, Indigenous, Latinx, and other people of color the most. *The Atlantic*. March 7, 2021. Accessed November 3, 2022. https://covidtracking.com/race

[49] Centers for Disease Control and Prevention (CDC). Deaths related to 2009 pandemic influenza A (H1N1) among American Indian/Alaska Natives - 12 states, 2009. MMWR Morb Mortal Wkly Rep. 2009;58(48):1341-1344. 20010508

[50] Hennessy TW, Bruden D, Castrodale L, ; Investigative Team. A case-control study of risk factors for death from 2009 pandemic influenza A(H1N1): is American Indian racial status an independent risk factor? Epidemiol Infect. 2016;144(2):315-324. doi:10.1017/S0950268815001211 26118767 PMC5222627

[51] Falsey AR, Hennessey PA, Formica MA, Cox C, Walsh EE. Respiratory syncytial virus infection in elderly and high-risk adults. N Engl J Med. 2005;352(17):1749-1759. doi:10.1056/NEJMoa043951 15858184

[52] Branche AR, Saiman L, Walsh EE, . Incidence of respiratory syncytial virus infection among hospitalized adults, 2017-2020. Clin Infect Dis. 2022;74(6):1004-1011. doi:10.1093/cid/ciab595 34244735

[53] Macias AE, McElhaney JE, Chaves SS, . The disease burden of influenza beyond respiratory illness. Vaccine. 2021;39(suppl 1):A6-A14. doi:10.1016/j.vaccine.2020.09.048 33041103 PMC7545338

[54] Follmann D, Janes HE, Buhule OD, . Antinucleocapsid antibodies after SARS-CoV-2 infection in the blinded phase of the randomized, placebo-controlled mRNA-1273 COVID-19 vaccine efficacy clinical trial. Ann Intern Med. 2022;175(9):1258-1265. doi:10.7326/M22-1300 35785530 PMC9258784

