# Supplementary material for: Clinical and Demographic Factors Associated With COVID-19, Severe COVID-19, and SARS-CoV-2 Infection in Adults: A Secondary Cross-Protocol Analysis of 4 Randomized Clinical Trials
Source: JAMA Netw Open. 2023 Jul 13;6(7):e2323349. doi: 10.1001/jamanetworkopen.2023.23349 (PMC10346130; doi:10.1001/jamanetworkopen.2023.23349)
Supplement: Supplement 1. — eMethods. eFigure 1. Cox Proportional Hazard Scaled Schoenfeld Residual Plots for Each Risk Factor eFigure 2. Smoothed Hazard Estimates of COVID-19 in Trial Placebo Participants Plotted by Calendar Time and Epidemiologic Trends eFigure 3. Univariate Cox Proportional Hazard Regression Models for the COVID-19 and Severe COVID-19 Coprimary End Points eFigure 4. Univariate Cox Proportional Hazard Regression Models for the Subclinical SARS-CoV-2 and Any SARS-CoV-2 Secondary End Points eFigure 5. Univariate Cox Proportional Hazard Regression Model for the Severe COVID-19 End Point Among Participants With COVID-19 eFigure 6. Multivariate Cox Proportional Hazard Regression Model for the Severe COVID-19 End Point Among Participants With COVID-19 eFigure 7. Univariate Cox Proportional Hazard Regression Model for the COVID-19 End Point Among Participants With SARS-CoV-2 eFigure 8. Multivariate Cox Proportional Hazard Regression Model for the COVID-19 End Point Among Participants With SARS-CoV-2 eFigure 9. Multivariate Cox Proportional Hazard Regression Models With 2-Way Interaction Terms for the COVID-19 End Point eTable 1. Study Case Definitions eTable 2. Schedule of Antinucleocapsid Protein Testing Across Protocols eTable 3. CDC Risk Factors for Severe Disease eTable 4. Study End Points Stratified by Trial eTable 5. Variant Counts From COVID-19 Cases [file jamanetwopen-e2323349-s001.pdf]

Supplemental Online Content

Theodore DA, Branche AR, Zhang L, et al; COVID-19 Prevention Network (CoVPN). Clinical and demographic factors associated with COVID-19, severe COVID-19, and SARS-CoV-2 infection in adults: a secondary cross-protocol analysis of 4 randomized clinical trials. *JAMA Netw Open*. 2023;6(7):e2323349. doi:10.1001/jamanetworkopen.2023.23349

eMethods.

eFigure 1. Cox Proportional Hazard Scaled Schoenfeld Residual Plots for Each Risk Factor

eFigure 2. Smoothed Hazard Estimates of COVID-19 in Trial Placebo Participants Plotted by Calendar Time and Epidemiologic Trends

eFigure 3. Univariate Cox Proportional Hazard Regression Models for the COVID-19 and Severe COVID-19 Coprimary End Points

eFigure 4. Univariate Cox Proportional Hazard Regression Models for the Subclinical SARS-CoV-2 and Any SARS-CoV-2 Secondary End Points

eFigure 5. Univariate Cox Proportional Hazard Regression Model for the Severe COVID-19 End Point Among Participants With COVID-19

eFigure 6. Multivariate Cox Proportional Hazard Regression Model for the Severe COVID-19 End Point Among Participants With COVID-19

eFigure 7. Univariate Cox Proportional Hazard Regression Model for the COVID-19 End Point Among Participants With SARS-CoV-2

eFigure 8. Multivariate Cox Proportional Hazard Regression Model for the COVID-19 End Point Among Participants With SARS-CoV-2

eFigure 9. Multivariate Cox Proportional Hazard Regression Models With 2-Way Interaction Terms for the COVID-19 End Point

eTable 1. Study Case Definitions

eTable 2. Schedule of Antinucleocapsid Protein Testing Across Protocols

eTable 3. CDC Risk Factors for Severe Disease

eTable 4. Study End Points Stratified by Trial

eTable 5. Variant Counts From COVID-19 Cases

This supplemental material has been provided by the authors to give readers additional information about their work.

© 2023 Theodore DA et al. *JAMA Network Open.*

eMethods

Study Outcomes

The primary Covid-19 study outcome of this analysis was determined by a harmonized definition across trials with minor differences as detailed below. All trials required molecular (PCR or NAAT) confirmation of SARS-CoV-2 infection in addition to listed symptoms.

The Moderna trial defined Covid-19 symptomatic illness as having one or more respiratory symptoms (cough, shortness of breath or pneumonia) or at least two systemic symptoms (fever ≥38°C, chills, myalgia, headache, sore throat, anosmia or ageusia). The AstraZeneca trial defined Covid-19 symptomatic illness as having 1 day of fever, dyspnea or shortness of breath or ≥2 days of chills, cough, myalgia, fatigue, headache, vomiting, diarrhea, anosmia, ageusia, sore throat, congestion, runny nose. The Janssen trial defined Covid-19 symptomatic illness as mild (FDA criteria for mild Covid-19), moderate (one or more: respiratory symptoms, DVT; or two or more: FDA criteria for moderate COVID-19, anosmia, ageusia, red or bruised looking feet or toes) and severe disease (FDA for severe or critical Covid-19 disease). The Novavax trial defined Covid-19 symptomatic illness as mild (FDA criteria for mild Covid-19), moderate (high fever ≥38.4°C for more than 3 days, evidence of significant lower respiratory tract infection) and severe (FDA criteria for severe or critical Covid-19 disease) disease.

Derivations of Variables

Baseline Comorbidities

In this meta-analysis, comorbid conditions (yes or no) were defined as the presence of a given medical condition indicated in either the medical history eCRF or the comorbid questionnaire CRF data. Comorbid conditions listed by CDC as associated with severe COVID-19 were first mapped to MedDRA coding. Specifically, the CDC updated on Feb 15, 2022 the listing of underlying medical conditions associated with higher risk for severe COVID-19 (https://www.cdc.gov/coronavirus/2019-ncov/science/science-briefs/underlying-evidence-table.html). All of the medical conditions in this CDC list were mapped to MedDRA 24.0- English version coding for the applicable Preferred Term (PT) and High-level Term (HLT) for each medical condition. The medical diagnosis listed in medical history eCRF of each participant were available as MedDRA terms. The frequencies of occurrence of each of these conditions were tabulated and included as an independent variable. The medical conditions and subcategories with a frequency of occurrence < 5% were combined or eliminated for the construction of variables analyzed in the final dataset (eTable 3). The mapped coding was then used to determine the presence of each condition as listed in the medical history eCRF.

Occupational Risk

Occupational risk was determined by attributing Occupational Safety and Health Administration (OSHA) hazard recognition scores to self-reported workplace information provided by participants. OSHA functions as a regulatory agency under the United States Department of Labor to ensure safe and healthful working conditions, and as such, defined categories in response to the Covid-19 pandemic to aid in the assessment and mitigation of exposure risk in the workplace. Low exposure risk jobs have minimal contact with the public or coworkers. Medium exposure risk jobs have frequent or sustained close contact with the public or coworkers in outdoor or well-ventilated settings. High exposure risk jobs include close or poorly ventilated working conditions with known or suspected sources of SARS-CoV-2 (such as a hospital, grocery store or public transit). Very high exposure risk jobs are performing specific medical, postmortem or laboratory procedures. If individuals selected more than one category, the maximum score was taken. High and very high exposure risk categories were combined for analysis.

Living Situation Risk

Living situation risk synthesizes variables across all four trials and is scored on a scale of low, medium, high, or very high risk. It is based on housing type for the Moderna trial and number of co-habitants for the other 3 trials. For the AstraZeneca, Janssen, and Novavax trials, low, medium, high, and very high risk conditions corresponded to 0-1, 2, 3 and 4 or more co-habitants, respectively. For the Moderna study, individuals self-reported the housing type(s) that applied. Each housing type was assigned to low (participants specified as not having risk of exposure related to housing), medium (single-family or detached housing, housing without shared entrances or elevators), high (congregate settings such as dormitory, group housing, or high density such as apartments with shared entrances or elevators), or very high (nursing homes, long-term care facilities, shelters, and multi-family dwellings) risk categories. If participants selected more than one housing type, the highest risk score was taken. If "other" was selected, a value was imputed using the most frequent category within a given study.

© 2023 Theodore DA et al. *JAMA Network Open.*

eFigure 1: Cox Proportional Hazard Scaled Schoenfeld Residual Plots for Each Risk Factor.

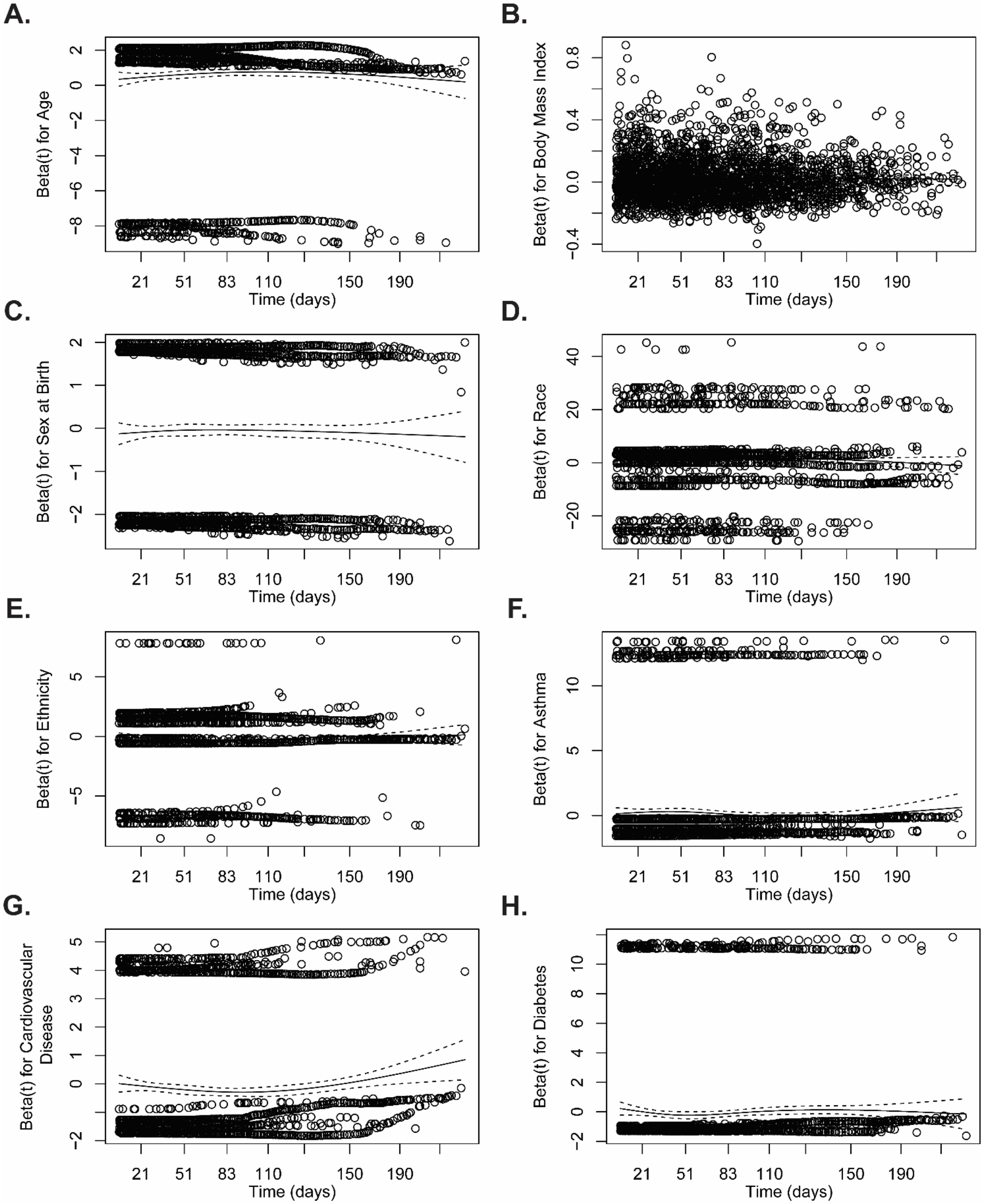

© 2023 Theodore DA et al. *JAMA Network Open.*

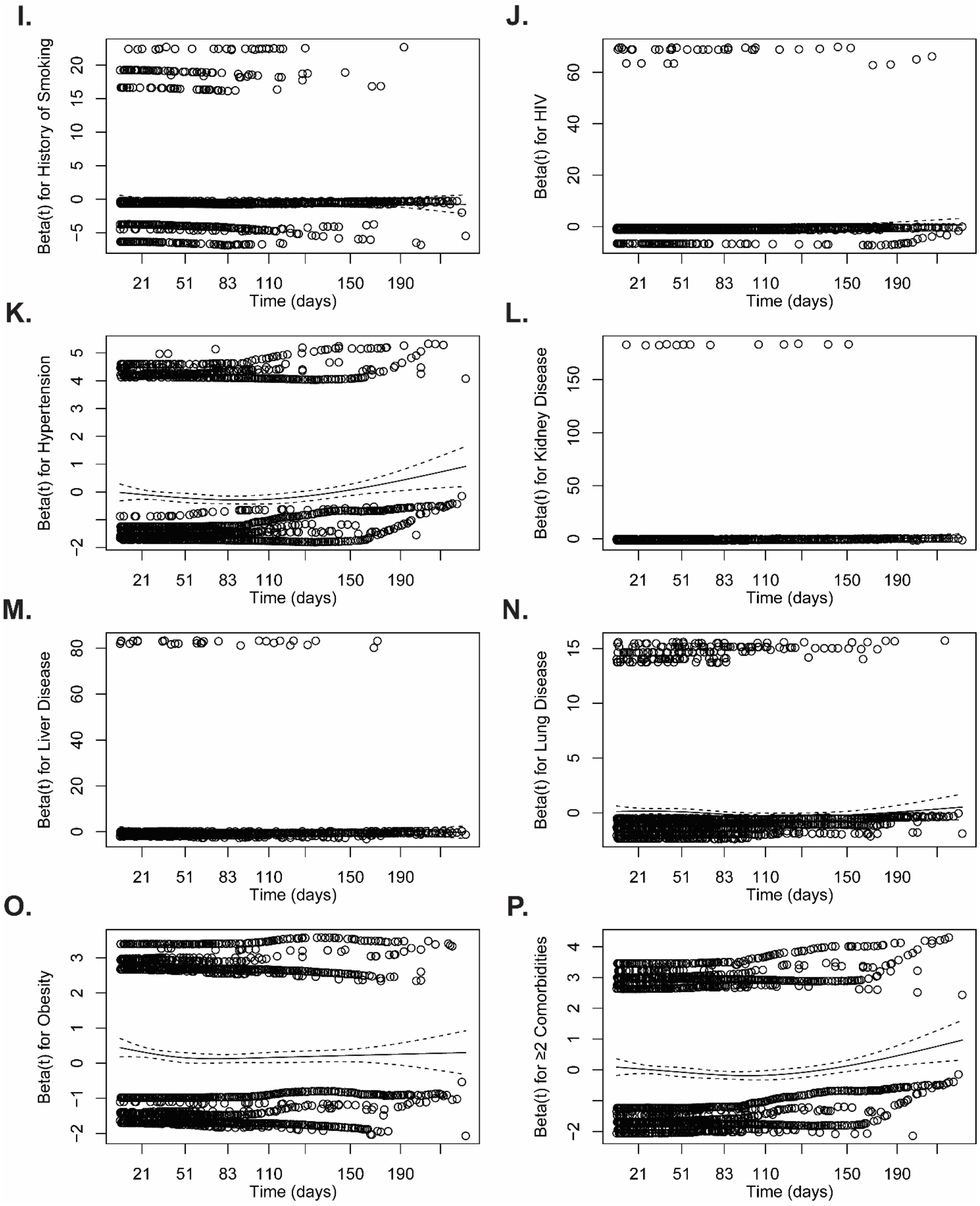

© 2023 Theodore DA et al. *JAMA Network Open.*

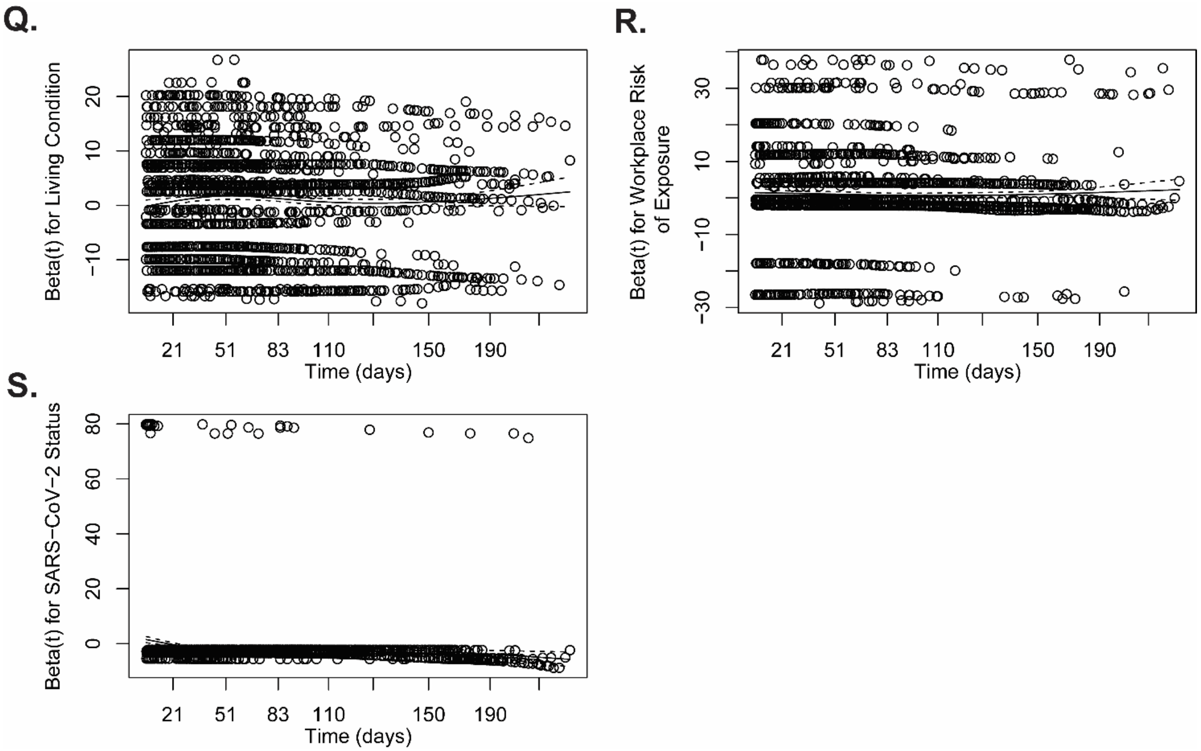

© 2023 Theodore DA et al. *JAMA Network Open.*

eFigure 2: Smoothed Hazard Estimates of COVID-19 in Trial Placebo Participants Plotted by Calendar Time and Epidemiologic Trends.

Smoothed hazard estimates are plotted over calendar time for the COVID-19 endpoint in each trial (Top). Epidemiological trends within the countries contributing data to the trials (top 4 countries for Janssen), including number of cases and emergence of viral variants, are presented directly below corresponding hazard estimates and based on information reported on COVID.who.int and gisaid.org. Variants of Interest or Concern accounting for ≥20% of a weekly submission for each region are included (see legend). The baseline curves on the epidemiologic figures express new cases within the trial. The solid black vertical line denotes the start of enrollment for each trial and the dashed black vertical line denotes the data cutoff point.

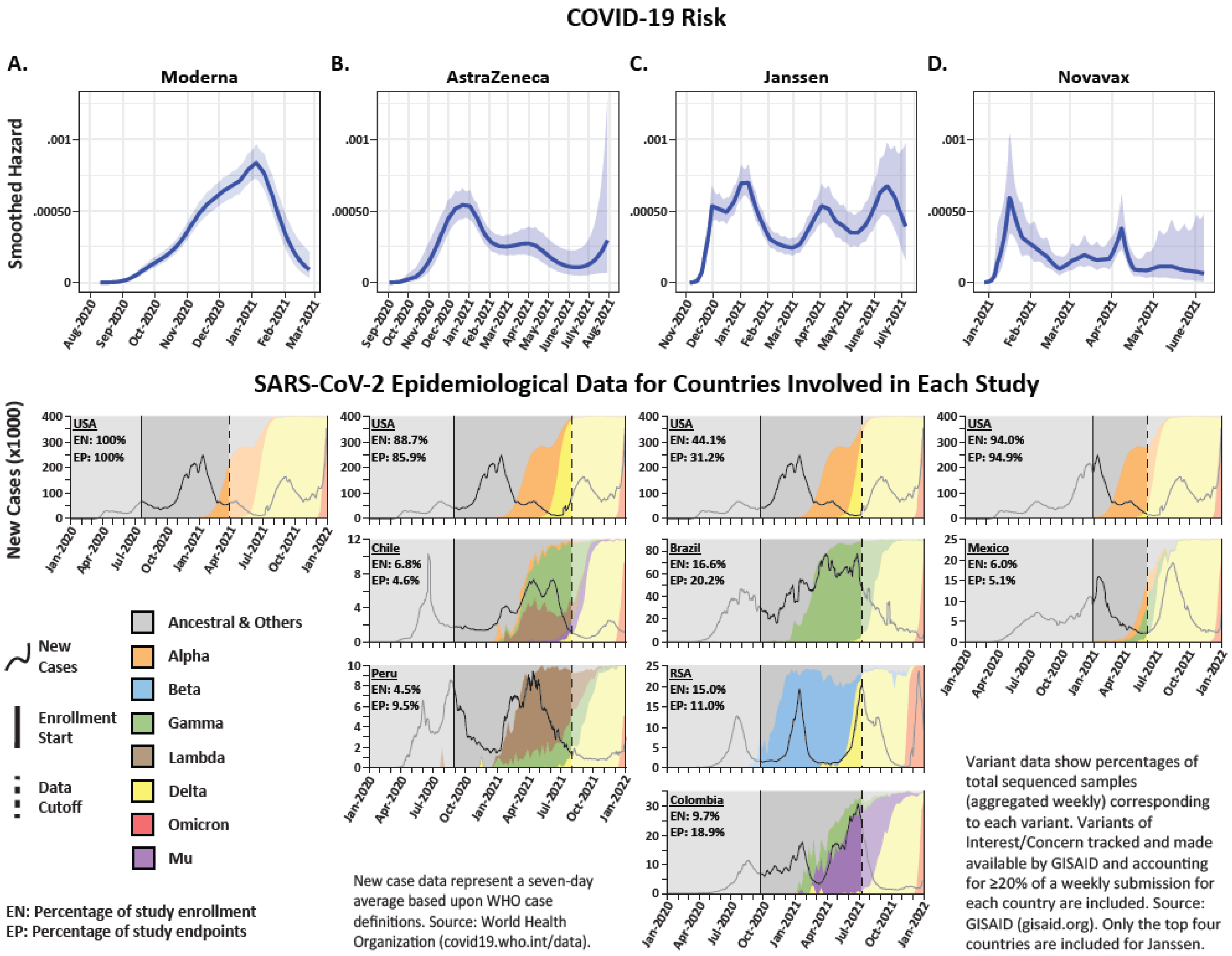

© 2023 Theodore DA et al. *JAMA Network Open.*

eFigure 3: Univariate Cox Proportional Hazard Regression Models for the COVID-19 and Severe COVID-19 Co-primary Endpoints.

All models adjusted for study (Moderna, AstraZeneca, Janssen, Novavax), Region (South America, North America, South Africa) and calendar time to account for potentially different baseline hazard functions across studies, regions, and time. Univariate hazard estimates for the two co-primary endpoints of COVID-19 (black, solid circle) and severe COVID-19 (red, open circle) are shown. The ≥3 Comorbidities and ≥4 Comorbidities variables were not considered for inclusion in the multivariate analysis. Unadjusted and adjusted P-values are shown. P-values ≤ 0.01 were considered statistically significant.

Covariates		HR (95% CI)	P-Value	Adj. P-Value
Age, in Years		0.99 (0.98 - 0.99)<br>1.01 (1.01 - 1.02)	<0.001<br><0.001	<0.001<br><0.001
Age	≥65 (vs. 18-64)	0.53 (0.47 - 0.60)<br>1.08 (0.83 - 1.41)	<0.001<br>0.56	<0.001<br>0.68
Sex	Male (vs. Female)	0.93 (0.86 - 1.01)<br>0.87 (0.71 - 1.06)	0.08<br>0.18	0.12<br>0.25
Race	American Indian or Alaska Native (vs. White)	0.97 (0.83 - 1.13)<br>2.03 (1.43 - 2.88)	0.001<br><0.001	0.002<br><0.001
	Asian (vs. White)	0.80 (0.63 - 1.02)<br>0.54 (0.22 - 1.32)		
	Black or African American (vs. White)	0.79 (0.68 - 0.92)<br>1.12 (0.78 - 1.62)		
	Other (vs. White)	1.15 (0.93 - 1.44)<br>1.05 (0.55 - 1.99)		
	Multiple (vs. White)	1.17 (0.98 - 1.39)<br>2.09 (1.43 - 3.05)		
Ethnicity	Hispanic or Latino (vs. Not Hispanic or Latino)	1.22 (1.09 - 1.36)<br>1.01 (0.72 - 1.42)	<0.001<br>0.94	0.002<br>0.95
Body Mass Index		1.02 (1.01 - 1.03)<br>1.05 (1.04 - 1.07)	<0.001<br><0.001	<0.001<br><0.001
Previous SARS-CoV-2 Infection	Y (vs. N)	0.14 (0.10 - 0.21)<br>0.04 (0.01 - 0.18)	<0.001<br><0.001	<0.001<br><0.001
Workplace Risk of Exposure	High (vs. Low)	1.42 (1.22 - 1.66)<br>0.91 (0.55 - 1.51)	<0.001<br>0.81	<0.001<br>0.90
	Medium (vs. Low)	1.50 (1.29 - 1.75)<br>1.01 (0.60 - 1.70)		
Living Condition	Very High Risk (vs. Low Risk)	1.42 (1.21 - 1.66)<br>0.95 (0.61 - 1.46)	<0.001<br>0.95	<0.001<br>0.95
	High Risk (vs. Low Risk)	1.17 (1.03 - 1.32)<br>0.92 (0.67 - 1.27)		
	Medium Risk (vs. Low Risk)	1.25 (1.13 - 1.38)<br>1.00 (0.78 - 1.30)		
Asthma	Y (vs. N)	1.16 (1.00 - 1.34)<br>1.41 (0.96 - 2.08)	0.05<br>0.09	0.08<br>0.14
Cardiovascular Disease	Y (vs. N)	0.85 (0.78 - 0.93)<br>1.61 (1.29 - 2.00)	<0.001<br><0.001	0.002<br><0.001
Diabetes	Y (vs. N)	0.96 (0.83 - 1.10)<br>2.17 (1.65 - 2.85)	0.52<br><0.001	0.60<br><0.001
History of Smoking	Y (vs. N)	0.80 (0.66 - 0.96)<br>1.24 (0.69 - 2.22)	0.01<br>0.49	0.02<br>0.65
HIV	Y (vs. N)	0.92 (0.66 - 1.27)<br>0.81 (0.38 - 1.74)	0.60<br>0.58	0.66<br>0.68
Hypertension	Y (vs. N)	0.86 (0.78 - 0.94)<br>1.64 (1.31 - 2.04)	<0.001<br><0.001	0.002<br><0.001
Kidney Disease	Y (vs. N)	1.09 (0.65 - 1.85)<br>3.35 (1.38 - 8.12)	0.75<br>0.03	0.75<br>0.05
Liver Disease	Y (vs. N)	1.31 (0.92 - 1.87)<br>2.44 (1.15 - 5.17)	0.16<br>0.04	0.20<br>0.07
Lung Disease	Y (vs. N)	0.97 (0.83 - 1.14)<br>1.53 (1.05 - 2.25)	0.72<br>0.04	0.75<br>0.07
Obesity	Y (vs. N)	1.23 (1.14 - 1.34)<br>1.96 (1.59 - 2.42)	<0.001<br><0.001	<0.001<br><0.001
≥2 Comorbidities	Y (vs. N)	0.96 (0.88 - 1.04)<br>1.88 (1.53 - 2.32)	0.29<br><0.001	0.36<br><0.001
≥3 Comorbidities	Y (vs. N)	0.92 (0.84 - 1.02)<br>1.98 (1.58 - 2.50)	0.13<br><0.001	0.18<br><0.001

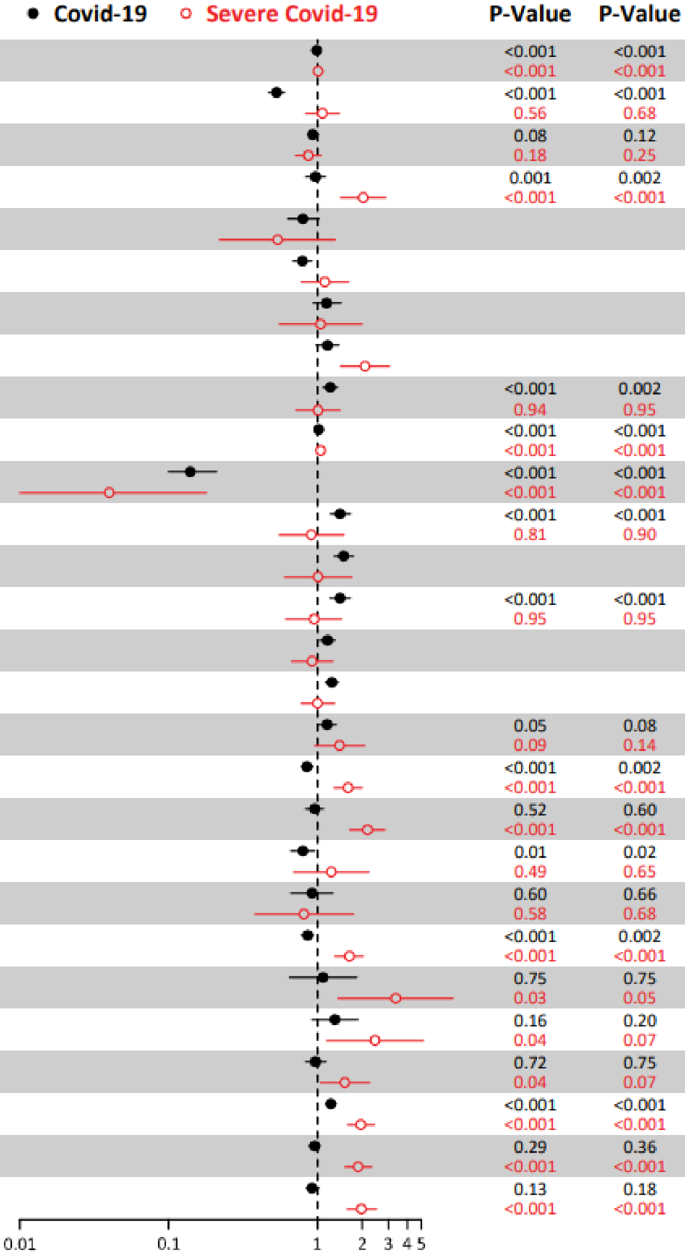

© 2023 Theodore DA et al. *JAMA Network Open.*

eFigure 4: Univariate Cox Proportional Hazard Regression Models for the Subclinical SARS-CoV-2 and Any SARS-CoV-2 Secondary Endpoints.

All models adjusted for study (Moderna, AstraZeneca, Janssen, Novavax), Region (South America, North America, South Africa) and calendar time to account for potentially different baseline hazard functions across studies, regions, and time. Univariate hazard estimates for the secondary endpoints, any SARS-CoV-2 infection (black, solid circle) and subclinical SARS-CoV-2 infection (blue, open circle) are shown. The ≥3 Comorbidities and ≥4 Comorbidities variables were not considered for inclusion in the multivariate analysis. Unadjusted and adjusted P-values are shown. P-values ≤ 0.01 were considered statistically significant.

Covariates		HR (95% CI)	P-Value	Adj. P-Value
**Age, in Years**		0.99 (0.99 - 0.99)	<0.001	<0.001
		0.99 (0.99 - 1.00)	<0.001	<0.001
**Age**	≥65 (vs. 18-64)	0.65 (0.59 - 0.71)	<0.001	<0.001
		0.81 (0.71 - 0.94)	0.004	0.01
**Sex**	Male (vs. Female)	0.94 (0.88 - 1.01)	0.08	0.14
		0.94 (0.85 - 1.04)	0.22	0.43
**Race**	American Indian or Alaska Native (vs. White)	1.11 (0.97 - 1.28)	<0.001	<0.001
		1.00 (0.76 - 1.31)	<0.001	<0.001
	Asian (vs. White)	0.71 (0.57 - 0.88)		
		0.57 (0.39 - 0.83)		
	Black or African American (vs. White)	1.19 (1.07 - 1.32)		
		1.61 (1.40 - 1.85)		
	Other (vs. White)	1.19 (0.99 - 1.43)		
		1.23 (0.92 - 1.65)		
	Multiple (vs. White)	1.18 (1.01 - 1.37)		
		1.26 (0.97 - 1.62)		
**Ethnicity**	Hispanic or Latino (vs. Not Hispanic or Latino)	1.11 (1.01 - 1.22)	0.04	0.08
		0.95 (0.82 - 1.11)	0.51	0.65
**Body Mass Index**		1.02 (1.01 - 1.02)	<0.001	<0.001
		1.01 (1.00 - 1.02)	0.002	0.008
**Workplace Risk of Exposure**	High (vs. Low)	1.46 (1.27 - 1.68)	<0.001	<0.001
		1.57 (1.26 - 1.95)	<0.001	<0.001
	Medium (vs. Low)	1.29 (1.12 - 1.49)		
		1.17 (0.94 - 1.46)		
**Living Condition**	Very High Risk (vs. Low Risk)	1.56 (1.37 - 1.77)	<0.001	<0.001
		1.50 (1.25 - 1.81)	<0.001	0.002
	High Risk (vs. Low Risk)	1.22 (1.10 - 1.35)		
		1.18 (1.02 - 1.38)		
	Medium Risk (vs. Low Risk)	1.22 (1.12 - 1.32)		
		1.09 (0.96 - 1.24)		
**Asthma**	Y (vs. N)	1.06 (0.94 - 1.20)	0.35	0.49
		1.03 (0.84 - 1.25)	0.80	0.88
**Cardiovascular Disease**	Y (vs. N)	0.88 (0.82 - 0.95)	<0.001	0.003
		0.92 (0.82 - 1.03)	0.14	0.37
**Diabetes**	Y (vs. N)	1.02 (0.91 - 1.14)	0.72	0.84
		1.09 (0.93 - 1.29)	0.29	0.46
**History of Smoking**	Y (vs. N)	0.92 (0.78 - 1.09)	0.32	0.49
		1.13 (0.90 - 1.42)	0.31	0.46
**HIV**	Y (vs. N)	1.19 (0.97 - 1.45)	0.10	0.18
		1.39 (1.09 - 1.79)	0.01	0.04
**Hypertension**	Y (vs. N)	0.89 (0.82 - 0.96)	0.002	0.005
		0.92 (0.82 - 1.04)	0.18	0.38
**Kidney Disease**	Y (vs. N)	1.03 (0.64 - 1.66)	0.91	1.00
		1.01 (0.45 - 2.26)	0.97	1.00
**Liver Disease**	Y (vs. N)	1.00 (0.71 - 1.42)	0.99	1.00
		0.65 (0.32 - 1.29)	0.18	0.39
**Lung Disease**	Y (vs. N)	0.97 (0.85 - 1.11)	0.66	0.81
		1.08 (0.88 - 1.31)	0.47	0.65
**Obesity**	Y (vs. N)	1.16 (1.08 - 1.24)	<0.001	<0.001
		1.06 (0.96 - 1.18)	0.26	0.46
**≥2 Comorbidities**	Y (vs. N)	0.95 (0.89 - 1.02)	0.19	0.31
		0.97 (0.87 - 1.08)	0.56	0.65
**≥3 Comorbidities**	Y (vs. N)	0.97 (0.89 - 1.05)	0.42	0.55
		1.04 (0.92 - 1.18)	0.52	0.65

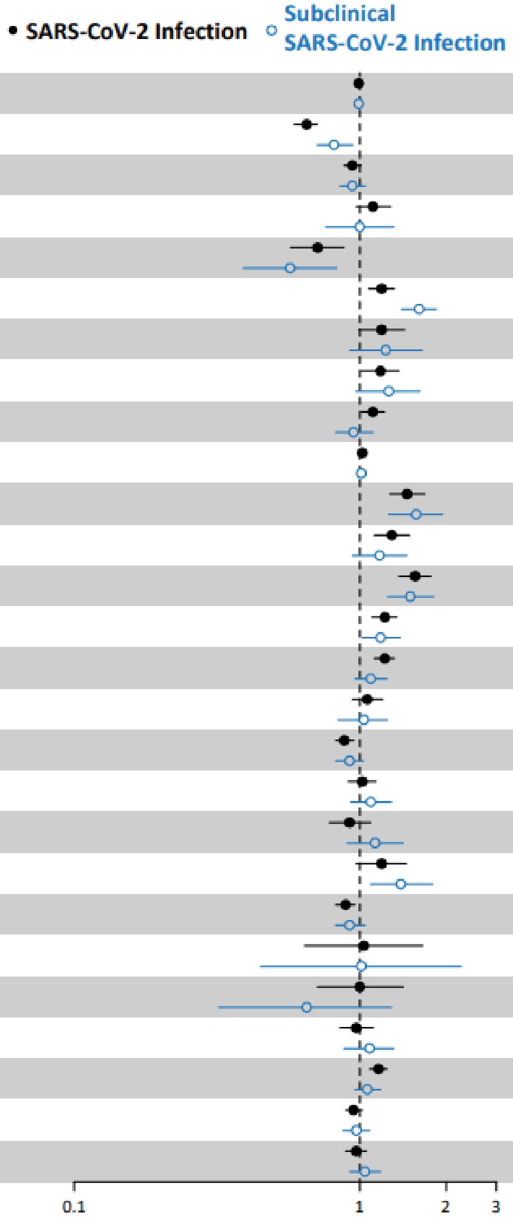

© 2023 Theodore DA et al. *JAMA Network Open.*

eFigure 5: Univariate Cox Proportional Hazard Regression Model for the Severe COVID-19 Endpoint among Participants with COVID-19.
All models adjusted for study (Moderna, AstraZeneca, Janssen, Novavax), Region (South America, North America, South Africa) and calendar time to account for potentially different baseline hazard functions across studies, regions, and time. Univariate hazard estimates for the co-primary endpoint Severe COVID-19 among participants with COVID-19 are shown. The ≥3 Comorbidities and ≥4 Comorbidities variables were not considered for inclusion in the multivariate analysis. Unadjusted and adjusted P-values are shown. P-values ≤ 0.01 were considered statistically significant.

Covariates		HR (95% CI)	P-Value	Adj P-Value
Age, in years		1.04 (1.03 - 1.04)	<0.001	<0.001
Age	≥65yr (vs. 18-64 yr)	2.18 (1.68 - 2.84)	<0.001	<0.001
Sex:	Male (vs. Female)	0.92 (0.75 - 1.13)	0.41	0.43
Race	American Indian or Alaska Native (vs. White)	1.80 (1.26 - 2.56)	0.003	0.007
	Asian (vs. White)	0.68 (0.28 - 1.65)		
	Black or African American (vs. White)	1.52 (1.04 - 2.21)		
	Other (vs. White)	0.91 (0.48 - 1.71)		
	Multiple (vs. White)	1.73 (1.17 - 2.56)		
Ethnicity	Hispanic or Latino (vs. Not Hispanic or Latino)	0.82 (0.58 - 1.15)	0.25	0.29
Body Mass Index		1.04 (1.02 - 1.05)	<0.001	<0.001
SARS-CoV-2 Status	Positive (vs. Negative)	0.35 (0.09 - 1.42)	0.08	0.11
Workplace risk of exposure to SARS-CoV-2	High (vs. Low)	0.63 (0.39 - 1.03)	0.16	0.21
	Medium (vs. Low)	0.67 (0.41 - 1.11)		
Living Condition	Very high risk (vs. Low risk)	0.59 (0.38 - 0.91)	0.04	0.06
	High risk (vs. Low risk)	0.72 (0.52 - 0.99)		
	Medium risk (vs. Low risk)	0.77 (0.59 - 0.99)		
Asthma	Y (vs. N)	1.25 (0.85 - 1.83)	0.28	0.31
Cardiovascular Disease	Y (vs. N)	1.99 (1.60 - 2.47)	<0.001	<0.001
Diabetes	Y (vs. N)	2.60 (1.98 - 3.42)	<0.001	<0.001
History of Smoking	Y (vs. N)	1.45 (0.80 - 2.62)	0.24	0.29
HIV	Y (vs. N)	0.97 (0.45 - 2.07)	0.94	0.94
Hypertension	Y (vs. N)	2.01 (1.51 - 2.50)	<0.001	<0.001
Kidney Disease	Y (vs. N)	3.75 (1.54 - 9.10)	0.02	0.03
Liver Disease	Y (vs. N)	2.35 (1.09 - 5.06)	0.05	0.08
Lung Disease	Y (vs. N)	1.65 (1.12 - 2.42)	0.02	0.03
Obesity	Y (vs. N)	1.69 (1.37 - 2.09)	<0.001	<0.001
≥2 Comorbidities	Y (vs. N)	2.07 (1.68 - 2.55)	<0.001	<0.001
≥3 Comorbidities	Y (vs. N)	2.34 (1.85 - 2.96)	<0.001	<0.001

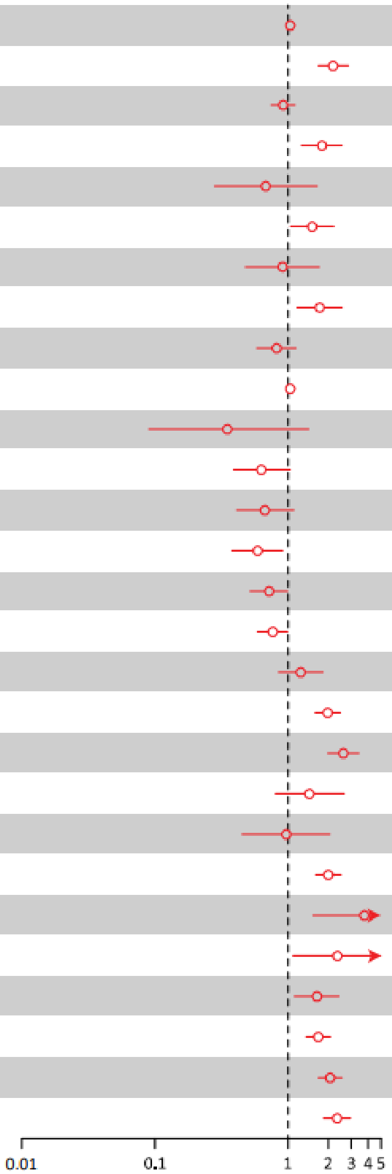

© 2023 The

eFigure 6: Multivariate Cox Proportional Hazard Regression Model for the Severe COVID-19 Endpoint among Participants with COVID-19.
All models adjusted for study (Moderna, AstraZeneca, Janssen, Novavax), Region (South America, North America, South Africa) and calendar time to account for potentially different baseline hazard functions across studies, regions, and time. Multivariate hazard estimates for the co-primary endpoint Severe COVID-19 among participants with COVID-19 are shown. The model was produced via Akaike information criterion in a stepwise algorithm. P-values ≤ 0.01 were considered statistically significant.

Covariates		HR (95% CI)	P-Value
**Age**	≥65yr (vs. 18-64 yr)	1.75 (1.32 - 2.31)	<0.001
**Race**	American Indian or Alaska Native (vs. White)	1.98 (1.38 - 2.83)	0.001
	Asian (vs. White)	0.82 (0.34 - 2.00)	
	Black or African American (vs. White)	1.49 (1.03 - 2.14)	
	Other (vs. White)	0.95 (0.50 - 1.81)	
	Multiple (vs. White)	1.81 (1.21 - 2.69)	
**Body Mass Index**		1.03 (1.01 - 1.04)	0.001
**SARS-CoV-2 Status**		0.33 (0.08 - 1.36)	0.12
**Living Condition**	Very high risk (vs. Low risk)	0.61 (0.40 - 0.95)	0.07
	High risk (vs. Low risk)	0.74 (0.53 - 1.02)	
	Medium risk (vs. Low risk)	0.78 (0.60 - 1.02)	
**Diabetes**	Y (vs. N)	1.85 (1.37 - 2.49)	<0.001
**Hypertension**	Y (vs. N)	1.36 (1.06 - 1.75)	0.02
**Lung Disease**	Y (vs. N)	1.49 (1.02 - 2.19)	0.05

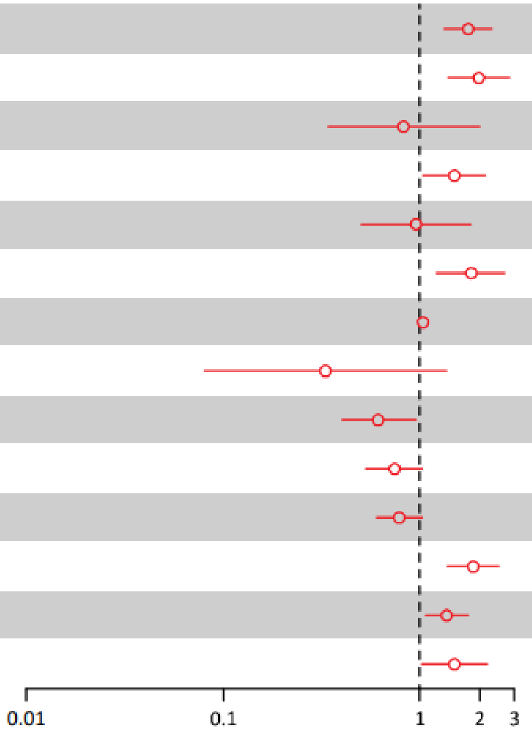

© 2023 Theodore DA et al. *JAMA Network Open.*

eFigure 7: Univariate Cox Proportional Hazard Regression Model for the COVID-19 Endpoint among Participants with SARS-CoV-2.
All models adjusted for study (Moderna, AstraZeneca, Janssen, Novavax), Region (South America, North America, South Africa) and calendar time to account for potentially different baseline hazard functions across studies, regions, and time. Univariate hazard estimates for the co-primary endpoint COVID-19 among participants with SARS-CoV-2 are shown. The ≥3 Comorbidities and ≥4 Comorbidities variables were not considered for inclusion in the multivariate analysis. Unadjusted and adjusted P-values are shown. P-values ≤ 0.01 were considered statistically significant.

Covariates		HR (95% CI)	P-Value	Adj P-Value
**Age, in years**		1.00 (1.00 - 1.00)	0.35	0.54
**Age**	≥65yr (vs. 18-64 yr)	1.05 (0.92 - 1.20)	0.46	0.66
**Sex:**	Male (vs. Female)	0.93 (0.85 - 1.01)	0.09	0.29
**Race**	American Indian or Alaska Native (vs. White)	0.63 (0.54 - 0.74)	<0.001	<0.001
	Asian (vs. White)	1.27 (0.98 - 1.65)		
	Black or African American (vs. White)	0.57 (0.48 - 0.67)		
	Other (vs. White)	0.95 (0.75 - 1.20)		
	Multiple (vs. White)	0.72 (0.59 - 0.86)		
**Ethnicity**	Hispanic or Latino (vs. Not Hispanic or Latino)	0.90 (0.80 - 1.02)	0.11	0.30
**Body Mass Index**		1.01 (1.00 - 1.01)	0.03	0.21
**Workplace risk of exposure to SARS-CoV-2**	High (vs. Low)	0.91 (0.76 - 1.10)	0.57	0.66
	Medium (vs. Low)	0.91 (0.75 - 1.10)		
**Living Condition**	Very high risk (vs. Low risk)	1.04 (0.88 - 1.24)	0.16	0.40
	High risk (vs. Low risk)	0.99 (0.87 - 1.14)		
	Medium risk (vs. Low risk)	1.12 (1.00 - 1.24)		
**Asthma**	Y (vs. N)	1.02 (0.87 - 1.20)	0.80	0.80
**Cardiovascular Disease**	Y (vs. N)	1.05 (0.95 - 1.16)	0.35	0.54
**Diabetes**	Y (vs. N)	0.96 (0.83 - 1.11)	0.59	0.66
**History of Smoking**	Y (vs. N)	0.77 (0.60 - 1.00)	0.04	0.21
**HIV**	Y (vs. N)	0.79 (0.56 - 1.13)	0.19	0.42
**Hypertension**	Y (vs. N)	1.03 (0.93 - 1.14)	0.55	0.66
**Kidney Disease**	Y (vs. N)	1.16 (0.64 - 2.10)	0.63	0.67
**Liver Disease**	Y (vs. N)	1.50 (1.00 - 2.25)	0.07	0.27
**Lung Disease**	Y (vs. N)	0.90 (0.75 - 1.08)	0.26	0.46
**Obesity**	Y (vs. N)	1.10 (1.00 - 1.20)	0.04	0.21
**≥2 Comorbidities**	Y (vs. N)	1.06 (0.96 - 1.16)	0.25	0.46
**≥3 Comorbidities**	Y (vs. N)	0.97 (0.86 - 1.09)	0.59	0.66

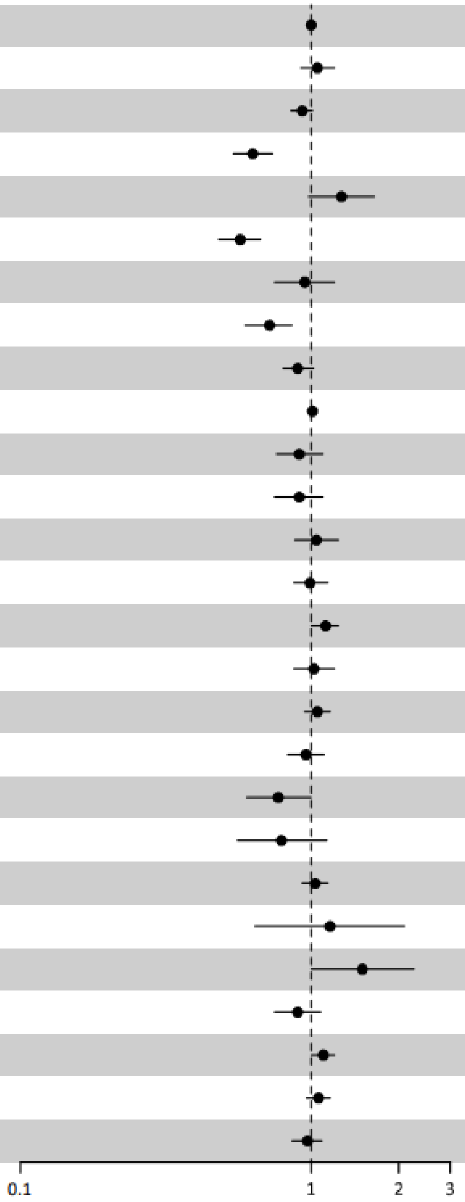

© 202[illegible]

eFigure 8: Multivariate Cox Proportional Hazard Regression Model for the COVID-19 Endpoint among Participants with SARS-CoV-2.

All models adjusted for study (Moderna, AstraZeneca, Janssen, Novavax), Region (South America, North America, South Africa) and calendar time to account for potentially different baseline hazard functions across studies, regions, and time. Multivariate hazard estimates for the co-primary endpoint COVID-19 among participants with SARS-CoV-2 are shown. The model was produced via Akaike information criterion in a stepwise algorithm. P-values ≤ 0.01 were considered statistically significant.

Covariates		HR (95% CI)	P-Value
Sex	Male (vs. Female)	0.94 (0.86 - 1.02)	0.15
Race	American Indian or Alaska Native (vs. White)	0.63 (0.54 - 0.75)	<0.001
	Asian (vs. White)	1.27 (0.98 - 1.66)	
	Black or African American (vs. White)	0.56 (0.47 - 0.67)	
	Other (vs. White)	1.02 (0.80 - 1.29)	
	Multiple (vs. White)	0.71 (0.59 - 0.86)	
Ethnicity	Hispanic or Latino (vs. Not Hispanic or Latino)	0.86 (0.76 - 0.98)	0.03
Body Mass Index		1.01 (1.00 - 1.01)	0.05
Living Condition	Very high risk (vs. Low risk)	1.08 (0.90 - 1.29)	0.10
	High risk (vs. Low risk)	1.02 (0.89 - 1.17)	
	Medium risk (vs. Low risk)	1.14 (1.02 - 1.27)	
History of Smoking	Y (vs. N)	0.76 (0.58 - 0.99)	0.04
Hypertension	Y (vs. N)	0.84 (0.69 - 1.02)	0.07
Liver Disease	Y (vs. N)	1.45 (0.96 - 2.21)	0.08
Lung Disease	Y (vs. N)	0.80 (0.65 - 1.00)	0.05
≥2 Comorbidities	Y (vs. N)	1.26 (1.04 - 1.53)	0.02

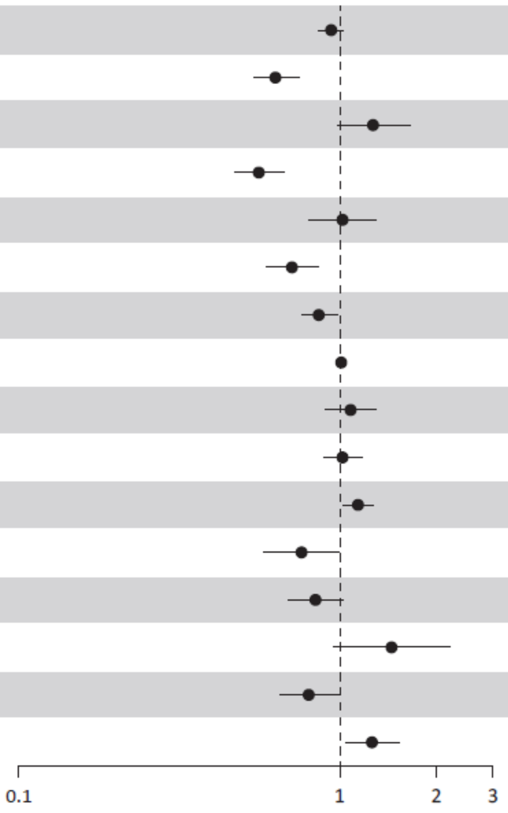

© 2023 Theodore DA et al. *JAMA Network Open.*

eFigure 9: Multivariate Cox Proportional Hazard Regression Models with Two-Way Interaction Terms for the COVID-19 Endpoint.

Two-way interaction terms were included in the Cox models via a cross-product term of the two covariates of interest. The Multivariate Cox model of COVID-19 with two-way interactions for age, ethnicity was chosen by Akaike information criterion, while keeping only interaction terms with a p-value <0.01. Not all variables that were significant in the univariate analysis were included in the multivariate model. P-values ≤ 0.01 for the adjusted hazard ratios were considered statistically significant.

Covariates	Interaction	HR (95% CI)	P-Value
Age: ≥65 yr (vs. 18-64 yr)	Workplace risk of exposure to SARS-CoV-2: Low	0.69 (0.58 - 0.81)	<0.001
	Workplace risk of exposure to SARS-CoV-2: Medium	0.59 (0.47 - 0.74)	
	Workplace risk of exposure to SARS-CoV-2: High	0.32 (0.24 - 0.43)	
Sex: Male (vs. Female)	Ethnicity: Not Hispanic or Latino	0.86 (0.77 - 0.95)	0.01
	Ethnicity: Hispanic or Latino	1.06 (0.93 - 1.19)	
Race: American Indian or Alaska Native (vs. White)		1.10 (0.94 - 1.28)	0.001
Race: Asian (vs. White)		0.79 (0.62 - 1.01)	
Race: Black or African American (vs. White)		0.78 (0.67 - 0.91)	
Race: Other (vs. White)		1.08 (0.87 - 1.35)	
Race: Multiple (vs. White)		1.18 (0.99 - 1.40)	
Ethnicity: Hispanic or Latino (vs. Not Hispanic or Latino)	SARS-CoV-2 Status: Negative	0.97 (0.84 - 1.12)	0.004
	SARS-CoV-2 Status: Positive	0.33 (0.16 - 0.69)	
	Sex: Female	0.97 (0.84 - 1.12)	0.01
	Sex: Male	1.20 (1.04 - 1.37)	
Body Mass Index		1.02 (1.01 - 1.03)	<0.001
SARS-CoV-2 Status: Positive (vs. Negative)	Ethnicity: Not Hispanic or Latino	0.23 (0.15 - 0.36)	0.004
	Ethnicity: Hispanic or Latino	0.08 (0.04 - 0.14)	
Workplace risk of exposure to SARS-CoV-2: High (vs. Low)	Age: 18-64 yr	1.44 (1.23 - 1.69)	<0.001
	Age: ≥65 yr	0.67 (0.47 - 0.96)	
Workplace risk of exposure to SARS-CoV-2: Medium (vs. Low)	Age: 18-64 yr	1.42 (1.21 - 1.66)	
	Age: ≥65 yr	1.22 (0.91 - 1.63)	
Living Condition: Very high risk (vs. Low Risk)		1.43 (1.22 - 1.68)	<0.001
Living Condition: High risk (vs. Low Risk)		1.13 (0.99 - 1.28)	
Living Condition: Medium risk (vs. Low Risk)		1.20 (1.09 - 1.33)	
Asthma Y (vs. N)		1.23 (1.00 - 1.51)	0.05
History of Smoking Y (vs. N)		0.81 (0.67 - 0.97)	0.03
HIV Y (vs. N)		0.99 (0.71 - 1.37)	0.95
Lung Disease Y (vs. N)		0.82 (0.65 - 1.02)	0.07

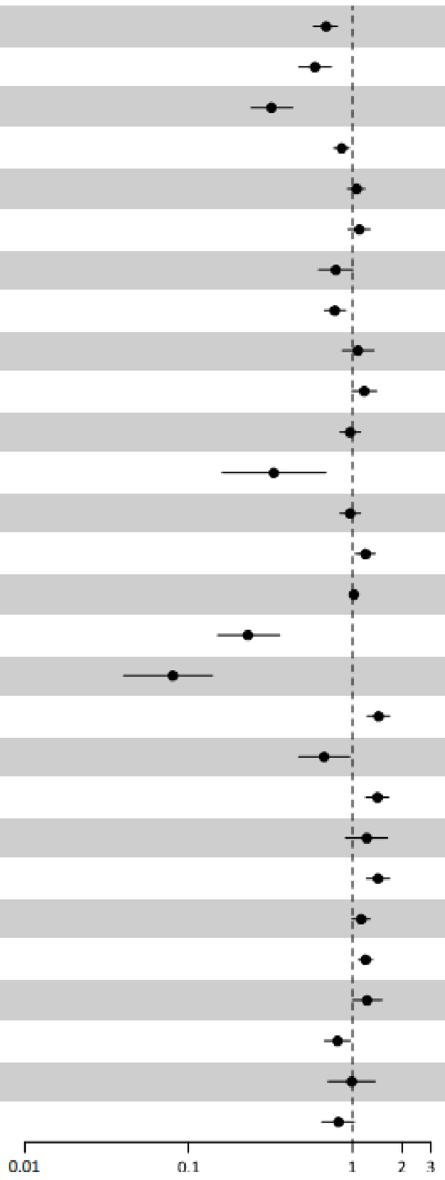

© 2023 Theodore DA et al. *JAMA Network Open.*

eTable 1. Study Case Definitions.

	Case Definition	
	**COVID-19**	**Severe COVID-19**
**Moderna**	PCR-confirmed SARS-CoV-2 infection AND one of the following: cough, shortness of breath or difficulty breathing, or clinical or radiological evidence of pneumonia; OR two of the following: fever (≥38° C), chills, myalgia, headache, sore throat, or new anosmia or ageusia.	Confirmed COVID-19 as per the Primary Efficacy Endpoint case definition, plus any of the following: clinical signs indicative of severe systemic illness (respiratory rate ≥ 30 per minute, heart rate ≥ 125 beats per minute, SpO2 ≤ 93% on room air at sea level or PaO2/FIO2 <300 mm Hg); respiratory failure or ARDS, (defined as needing high-flow oxygen, non-invasive or mechanical ventilation, or extracorporeal membrane oxygenation); evidence of shock (systolic blood pressure < 90 mmHg, diastolic BP < 60 mmHg or requiring vasopressors); significant acute renal, hepatic or neurologic dysfunction; admission to an ICU; or death.
**AstraZeneca**	PCR-confirmed SARS-CoV-2 infection AND one of the following: new or worsening dyspnea/shortness of breath, pneumonia diagnosed by chest X-ray or computed tomography scan, or oxygen saturation ≤ 94% on room air or requiring either new initiation or escalation in supplemental O2; OR two of the following: fever, new/worsening cough, myalgia, fatigue that interferes with activities of daily living, vomiting and/or diarrhea, or anosmia and/or ageusia.	SARS-CoV-2 RT-PCR-positive symptomatic illness plus any of the following: clinical signs at rest indicative of severe systemic illness (respiratory rate ≥ 30 breaths per minute, heart rate ≥ 125 beats per minute, oxygen saturation ≤ 93% on room air at sea level, or PaO2/FIO2 < 300 mm Hg); respiratory failure (defined as needing high-flow oxygen, noninvasive ventilation, mechanical ventilation or extracorporeal membrane oxygenation); evidence of shock (systolic blood pressure < 90 mm Hg, diastolic blood pressure < 60 mm Hg, or requiring vasopressors); significant acute renal, hepatic, or neurologic dysfunction; admission to an ICU; or death.
**Janssen**	PCR or NAAT confirmation of SARS-CoV-2 infection AND ≥2 of the following symptoms (new or worsening): fever or chills, cough, heart rate ≥90 beats/minute, muscle or body pain, headache, new loss of taste or smell, sore throat, red or bruised-looking feet or toes, nausea, vomiting, or diarrhea; or one or more of the following signs or symptoms: shortness of breath, respiratory rate >20 breaths/minute, clinical or radiologic evidence of pneumonia, deep vein thrombosis, or abnormal oxygen saturation (but above 93%).	SARS-CoV-2 RT-PCR-positive symptomatic illness with any of the following: respiratory failure; evidence of shock (systolic blood pressure <90mm Hg, diastolic blood pressure <60 mm Hg, or requiring vasopressors); respiratory rate >30 breaths/minute; heart rate ≥125 beats/minute; oxygen saturation of 93% or less (ambient air at sea level), or a ratio of the partial pressure of oxygen to the fraction of inspired oxygen <300 mm Hg; intensive care unit admission; significant acute renal, hepatic, or neurologic dysfunction; or death.

© 2023 Theodore DA et al. *JAMA Network Open.*

**Novavax**	PCR-confirmed COVID-19, either mild (≥ 1 of the following: subjective or objective fever or new onset cough; or ≥ 2 of the following: new onset or worsening of shortness of breath or difficulty breathing compared to baseline, new onset fatigue, new onset generalized muscle or body aches, new onset headache, new loss of taste or smell, acute onset of sore throat, congestion or runny nose, new onset nausea, vomiting or diarrhea) OR moderate (≥ 1 of the following: fever ≥ 38.4°C for ≥ 3 days, any evidence of significant lower respiratory tract infection [shortness of breath or breathlessness or difficulty breathing with or without exertion greater than baseline], tachypnea [24 to 29 breaths per minute at rest], SpO2 94% to 95% on room air, abnormal chest X-ray or chest computerized tomography consistent with pneumonia or lower respiratory tract infection, adventitious sounds on lung auscultation [e.g., crackles/rales, wheeze, rhonchi, pleural rub, stridor].	SARS-CoV-2 RT-PCR positive symptomatic illness with any of the following: clinical signs at rest indicative of severe systemic illness (respiratory rate ≥30 breaths/minute, heart rate ≥125 beats/ minute, SpO2 ≤93% on room air at sea level, or PaO2/FiO2 <300 mmHg); respiratory failure (defined as needing high-flow oxygen, non-invasive ventilation, mechanical ventilation, or extracorporeal membrane oxygenation); one or more major organ system dysfunction or failure to be defined by diagnostic testing/clinical syndrome/interventions, including ARDS, acute renal failure, acute hepatic failure, acute right or left heart failure; septic or cardiogenic shock (with shock defined as systolic blood pressure < 90 mm Hg OR diastolic blood pressure < 60 mm Hg); acute stroke (ischemic or hemorrhagic), acute thrombotic event (acute myocardial infarction, deep vein thrombosis, pulmonary embolism); requirement for: vasopressors, systemic corticosteroids, or hemodialysis; multisystem inflammatory syndrome in children as per the CDC definition (in participants <21 years), ICU admission; or death.
**FDA**	Virologically confirmed SARS-CoV-2 infection with one or more of the following symptoms: fever or chills, cough, shortness of breath or difficulty breathing, fatigue, muscle or body aches, headache, new loss of taste or smell, sore throat, congestion or runny nose, nausea or vomiting, or diarrhea.	Virologically confirmed SARS-CoV-2 infection with any of the following: Clinical signs at rest indicative of severe systemic illness (respiratory rate ≥ 30 per minute, heart rate ≥ 125 per minute, SpO2 ≤ 93% on room air at sea level or PaO2/FiO2 < 300 mm Hg); respiratory failure (defined as needing high-flow oxygen, noninvasive ventilation, mechanical ventilation or extracorporeal membrane oxygenation); evidence of shock (SBP < 90 mm Hg, DBP < 60 mm Hg, or requiring vasopressors); significant acute renal, hepatic, or neurologic dysfunction; admission to an ICU; or death.

Abbreviations: PCR, polymerase chain reaction; NAAT, nucleic acid amplification test; FDA, US Food and Drug Administration; ARDS, Acute Respiratory Distress Syndrome; PaO2/FIO2, partial pressure of oxygen to fraction of inspired oxygen ratio; ICU, Intensive Care Unit. Participants meeting the severe COVID-19 case definition were included in the COVID-19 primary endpoint.

© 2023 Theodore DA et al. *JAMA Network Open.*

eTable 2: Schedule of Anti-Nucleocapsid Protein Testing Across Protocols. Planned testing is shown through week 30 of the studies. Ranges are given for later timepoints to account for slight differences in the individual trials. Only data from samples accrued through the blinded, pre-crossover phases of the trials are included in this analysis. Participants were screened for symptomatic illness by passive surveillance and active surveillance at all study visits. In Moderna, routine non-symptom-driven SARS-CoV-2 PCR testing was performed at week 0, week 4, and at the Participant Decision Visit (this visit provided the opportunity for participants to be unblinded following Emergency Use Authorization (EUA) of COVID-19 vaccines). In Novavax, routine non-symptom-driven SARS-CoV-2 PCR testing was performed at week 0 and at the time of blinded crossover following EUA submission.

Study	**Scheduled, per protocol, by study week**									
	**0**	**2**	**4-5**	**6**	**8**	**10**	**12-13**	**24-26**	**30**	**Unblinding or Crossover Visit Testing**
**Moderna**	x		x		x				x	x
**AstraZeneca**	x	x[a]	x	x[a]	x		x	x		
**Janssen**	x		x			x		x		x
**Novavax**	x		x				x	N/A	N/A	x

[a]Substudy participants only, n=3000.

© 2023 Theodore DA et al. *JAMA Network Open.*

eTable 3: CDC Risk Factors for Severe Disease.

CDC Category	Condition	Sub-condition	Supporting Literature	Decision to Include	MedDRA term (Version 24.0)
Mixed evidence	Alpha 1 antitrypsin deficiency		CDC systematic review of 6 studies, inconclusive for mortality/ICU/intubation/ventilation/hospitalization, insufficient to assess confounding	Yes	Alpha-1 antitrypsin deficiency (PT 10083869)
Mixed evidence	Asthma		Meta-analysis, review, cohort studies without clear direction of association	Yes	Asthma (PT 10003553) Childhood asthma (PT 10081274) Asthma late onset (PT 10003559) Occupational asthma (PT 10070836) Cough variant asthma (PT 10063076) Asthma exercise induced (PT 10003557)
Mixed evidence	Bronchopulmonary dysplasia		CDC systematic review found 1 relevant study, descriptive cohort of 185 peds inpatients: among 9 with bronchopulmonary dysplasia, no deaths, 2/9 ICU admit, 4/9 noninvasive ventilation	Yes	Bronchopulmonary dysplasia (PT 10006475)
Higher risk	Cancer		Meta-analysis (n=18k pts with COVID + cancer with search terms "cancer" "tumor" "malignancy" "neoplasia" through Jul 2020) generated pooled CFR of 25%; meta-analysis found "malignancy" OR 2.7 for composite severe outcomes (2.8 for severe disease, 1.7 for ICU, 3.2 for mortality); meta-analysis of 181k COVID pts (23k with cancer with search term "tumor OR cancer OR malignancy OR neoplasm") found OR 2.5 for death, higher in hematologic malignancy and lung cancer; prospective cohort n=800 with cancer + COVID through Apr 2020 found 28% mortality, not associated with cancer therapy; prospective cohort of 928 pts with cancer + COVID through Apr 2020 found 13% mortality, associated with active cancer vs remission	Yes	Neoplasms malignant site unspecified (HLT 10029105), neoplasm malignant (PT 10028997), neoplasm (PT 10028980)
Higher risk	Cerebrovascular disease		Meta-analysis (n=4k COVID pts) suggests cerebrovascular disease (search terms "cerebrovascular disease" "stroke" "brain infarction") associated with composite mortality + severe COVID (RR 2.0); separate analysis of 65k COVID pts suggests cerebrovascular disease (term "cerebrovascular disorders" and "stroke") associated with mortality (RR 2.2 but CI includes null); review from early in pandemic suggests cerebrovascular disease associated with mortality (RR 3.3); cohort	Yes	Cerebrovascular disorder (PT 10008196)

© 2023 Theodore DA et al. *JAMA Network Open.*

CDC Category	Condition	Sub-condition	Supporting Literature	Decision to Include	MedDRA term (Version 24.0)
			n=1590 suggests associated with mortality (HR 3.1, CI 1.1-8.9)		
Higher risk	Chronic kidney disease		Meta-analysis (n=27k COVID pts) found renal diseases (including Cr >1.1 or 1.3, also urinary tract issues such as infection, kidney stone, prostate disease) associated with COVID death. Large UK cohort found increased HR for COVID death with decreasing GFR (HR>2 for GFR<30) and with ESRD (HR 3.7)	Yes	Chronic kidney disease (PT 10064848)
	Chronic liver disease				
Higher risk	Chronic liver disease	Alcoholic liver disease	Limited (CDC systematic review found 1 study, n=1701): alcoholic liver disease associated with increased risk of mortality vs no liver disease	Yes	Alcoholic liver disease (PT 10001627) Fatty liver alcoholic (PT 10016262)
Higher risk	Chronic liver disease	Autoimmune hepatitis	Limited (CDC systematic review found 1 study, n=1701): propensity score matching indicates AIH may be associated with increased risk of severe outcomes including mortality, ICU, ventilation, but CI included the null	Yes	Autoimmune hepatitis (PT 10003827), Hepatic autoimmune disorders (HLT 10003820)
Higher risk	Chronic liver disease	Cirrhosis	(CDC systematic review of 64 cohort and case/control): cirrhosis associated with increased risk of mortality & hospitalization vs no cirrhosis	Yes	Hepatic cirrhosis (PT 10019641)
Higher risk	Chronic liver disease	Non-alcoholic fatty liver disease	CDC systematic review: NAFLD not associated with mortality. Possible increased risk ICU admission (4 studies) and mechanical ventilation (1 study)	Yes	Nonalcoholic fatty liver disease (PT 10082249)
Higher risk	Chronic liver disease	not specified	CDC systematic review of 64 cohort and case/control: any chronic liver disease associated with increased mortality, ICU admission, intubation, ventilation, hospitalization	Yes	Liver disorder (PT 10024670) Hepatic and hepatobiliary disorders NEC (HLT 10027681) Hepatic fibrosis and cirrhosis (HLT 10019669) Hepatocellular damage and hepatitis NEC (HLT 10019833)
	Chronic lung disease			Yes	Chronic respiratory disease (PT 10061768) Respiratory tract disorders (HLT 10024974) Bronchospasm and obstruction (HLT 10006484) Parenchymal lung disorders (HLT 10033979)

© 2023 Theodore DA et al. *JAMA Network Open.*

CDC Category	Condition	Sub-condition	Supporting Literature	Decision to Include	MedDRA term (Version 24.0)
Higher risk	Chronic lung disease	Bronchiectasis	CDC systematic review of 4 cohort studies: suggest increased risk of mortality (inconsistent) and ICU admission (CI included null). 1 study suggested increased risk of hospitalization	Yes	Bronchiectasis (PT 10006445) Congenital bronchiectasis (PT 10010391)
Higher risk	Chronic lung disease	COPD (chronic obstructive pulmonary disease)	Meta-analysis (n=1592 COVID pts) suggests COPD associated with severe disease (OR 5.7); meta-analysis (n=39k hospitalized COVID pts) suggests associated with severe disease (RR 1.7) and death (RR 2.0); meta-analysis (n=26k COVID pts) suggests associated with severe outcomes (pooled effect estimate 1.5)	Yes	Chronic obstructive pulmonary disease (PT 10009033) Asthma-chronic obstructive pulmonary disease overlap (PT 10077005) Obstructive airways disorder (PT 10061877)
Higher risk	Chronic lung disease	Interstitial lung disease	CDC systematic review of 7 cohort and 1 modeling study: suggest increased mortality, ICU admission. This is true for idiopathic pulm fibrosis (IPF) and sarcoid. Hypersensitivity pneumonitis and other ILD also associated with mortality and hospitalization	Yes	Interstitial lung disease (PT 10022611) Idiopathic pulmonary fibrosis (PT 10021240) Pulmonary fibrosis (PT 10037383) idiopathic pulmonary fibrosis (PT 10021240) Pulmonary sarcoidosis (PT 10037430) Hypersensitivity pneumonitis (PT 10081988)
Higher risk	Chronic lung disease	Pulmonary embolism	CDC systematic review found 1 cohort study which suggests history of PE is associated with mortality	Yes	Pulmonary embolism (PT 10037377) Pulmonary thrombosis (PT 10037437) Pulmonary artery thrombosis (PT 10037340)
Higher risk	Chronic lung disease	Pulmonary hypertension	CDC systematic review of 3 cohort studies: suggests Pulm HTN associated with increased mortality, ICU admission, hospitalization	Yes	Pulmonary hypertensions (HLT 10037401) (PT pulmonary hypertension10037400, pulmonary arterial hypertension10064911, pulmonary venous hypertension10085364)
Higher risk	Cystic fibrosis		Series (n=181 registry pts with CF including 32 post-transplant) through Jun 2020 reported 6% ICU admission, 3.8% mortality; series (n=40 registry pts with CF including 11 post-transplant) through Apr 2020 reported 10% ICU admission, 3% invasive ventilation, 0 deaths	Yes	Cystic fibrosis, Cystic fibrosis lung (PT 10011762, PT 10011763)
Higher risk	Diabetes mellitus			Yes	Diabetes mellitus (incl subtypes) (HLT 10012602) Hyperglycemic conditions NEC (HLT 10020638)
Higher risk	Diabetes mellitus	type 1	Large cohort study (263k w/DM1, >2 million with DM2) found associated with COVID-related death (OR of 2.8 and 1.8). Smaller study (40 with DM1,	Yes	Type 1 diabetes mellitus (PT 10067584)

© 2023 Theodore DA et al. *JAMA Network Open.*

CDC Category	Condition	Sub-condition	Supporting Literature	Decision to Include	MedDRA term (Version 24.0)
			273 with DM2) found associated with hospitalization and severe disease		
Higher risk	Diabetes mellitus	type 2	Other cohort studies find pre-existing, new dx, and hyperglycemia on admission associated with severe outcomes. Meta-analysis (n=438k pregnant pts) finds gestational DM associated with severe disease	Yes	Type 2 diabetes mellitus (PT 10067585), Insulin requiring type 2 diabetes mellitus (PT 10053247)
Higher risk	Disability	Attention-Deficit/Hyperactivity Disorder (ADHD)	CDC systematic review for disabilities (pending per website)	Yes	Attention deficit hyperactivity disorder (PT 10083622) Atypical attention deficit syndrome (PT 10003746)
Higher risk	Disability	Cerebral Palsy		Yes	Cerebral palsy (PT 10008129) Acquired cerebral palsy (PT 10084737)
Higher risk	Disability	Congenital Malformations (Birth Defects)		Yes	Congenital anomaly (PT 10010356)
Higher risk	Disability	Intellectual and Developmental Disabilities		Yes	Intellectual disabilities (HLT 10077548) Developmental disorders cognitive (HLT 10012561) Pervasive developmental disorders NEC (HLT 10034740)
Higher risk	Disability	Learning Disabilities		Yes	Learning disability (PT 10024092) Learning disorders (HLT 10024094)
Higher risk	Disability	Limitations with self-care or activities of daily living		Yes	Impaired self-care (PT 10052404) Loss of personal independence in daily activities (PT 10079487)
Higher risk	Disability	other disability		Yes	Amputee (PT 10075307) Bedridden (PT 100048948) Dependence on oxygen therapy (PT 10079637) Device dependence (PT 10078340) Disability (PT 10013050) Physical disability (PT 10048624) Prosthesis user (PT 10053669) Sight disability (PT 10053154) Hearing disability (PT 10053194) Walking aid user (PT 10050778) Walking disability (PT 10053204) Wheelchair user (PT 10047920)

© 2023 Theodore DA et al. *JAMA Network Open.*

CDC Category	Condition	Sub-condition	Supporting Literature	Decision to Include	MedDRA term (Version 24.0)
Higher risk	Disability	Spinal Cord Injuries		Yes	Spinal cord injury (PT 10041552) Injury to spinal cord secondary to birth trauma (PT 10022343) Spinal cord injuries NEC (HLT 10027662) Spinal cord and nerve root disorders traumatic (HLT 10041545)
Higher risk	Heart condition	cardiomyopathy	Meta-analyses from 2020 in May (n=1576), Aug (n=3027), Oct (n=2794) suggest "cardiovascular disease" NOS associated with severe infection (OR 2.5, 5.2, 2.8). Cohort studies (pub Jul-Aug 2020) suggest CAD or chronic heart disease (heart failure, ischemic heart disease, severe valvular or congenital heart disease) associated with risk of death	Yes	Cardiomyopathies (HLT 10007635)
Higher risk	Heart condition	coronary artery disease		Yes	Coronary artery disorders NEC (HLT 10011083) Ischemic coronary artery disorders (HLT 10011085)
Higher risk	Heart condition	heart failure		Yes	Heart failures NEC (HLT 10019281) Left ventricular failures (HLT 10024120) Right ventricular failures (HLT 10039164)
Higher risk	Heart condition	not specified			
Mixed evidence	Hepatitis B		CDC systematic review of 64 cohort and case/control: HBV not associated with increased risk of severe outcomes	Yes	Hepatitis B (PT 10019731) Chronic hepatitis B (PT 10008910) Congenital hepatitis B infection (PT 10010496) Perinatal HBV infection (PT 10075233) Hepatitis B reactivation (PT 10058827)
Mixed evidence	Hepatitis C		CDC systematic review of 64 cohort and case/control: HCV not associated with mortality or ICU admission	Yes	Hepatitis C (PT 10019744) Chronic hepatitis C (PT 10008912) Congenital hepatitis C infection (PT 10084252)
Higher risk	HIV		Meta-analysis/systematic review (20.9 million patients) RR for infection 1.2 (1.05-1.46), RR for mortality 1.78 (1.21-2.60), cohort studies	Yes	HIV infection (PT 10020161), Acquired immunodeficiency syndrome (PT 10000565) Asymptomatic HIV infection (PT 10003581) Congenital HIV infection (PT 10010504)
Mixed evidence	Hypertension		Meta-analysis, systematic review, cohort studies without clear direction of association	Yes	Hypertension (PT 10020772) White coat hypertension (PT 10051581) Vascular hypertensive disorders NEC (HLT 10020774)

© 2023 Theodore DA et al. *JAMA Network Open.*

CDC Category	Condition	Sub-condition	Supporting Literature	Decision to Include	MedDRA term (Version 24.0)
Higher risk	Mental health disorder	Mood disorders, including depression	Meta-analysis (21 studies, >91 million overall pts) suggests higher odds of hospitalization (1.3) & death (OR 1.5) in those with mood disorders (depression or bipolar), no associated with severe events (ICU/ventilation/O2/ECMO/ARDS/CPR)	Yes	Mood disorders NEC (HLT 10027948) Fluctuating mood symptoms (PT 10016798) Emotional and mood disturbances NEC (HLT 10014556) Affect alterations NEC (HLT 10001438) Depressive disorders (HLT 10012401) Mood alterations with depressive symptoms (HLT 10027938)
Higher risk	Mental health disorder	Schizophrenia spectrum disorders	Meta-analysis (16 studies, 19k patients with mental health disorder including mood, depression, anxiety, bipolar, schizophrenia, personality, eating, EtOH, substance use) suggests higher odds of death (OR 1.4) compared to those w/o mental health disorder, higher risk with severe mental health disorder	Yes	Schizophrenia NEC (HLT 10039631)
Higher risk	Neurologic Condition	Dementia	Meta-analysis (2) and review (1) suggest higher mortality among patients with dementia (OR 3.7 or OR 5.2)	Yes	Alzheimer's disease incl subtype (HLT 10001897), Dementia excl Alzheimer's (HLT 10012268) Dementia NEC (HLT 10012289) Vascular dementia disorders (HLT 10062914)
Higher risk	Obesity (BMI ≥30 kg/m2)		Systematic Review and Meta-Analysis (9 - n=4444) pooled OR 2.31 severe disease. See overweight	Yes	Obesity (PT 10029883)
Suggestive higher risk	Overweight (BMI ≥25 kg/m2, but <30 kg/m2)		Cohort (England 334329) OR hospitalization early 2020 Overweight 1.39 (1.13-1.71) OR obese 1.70 (1.34-2.16) maintains on adjustment for smoking, PE, EtOH, diabetes, HTN. Not if looking at labs cholesterol and A1c. Cohort (Ney York SUNY n=684). increase aRR overweight and obese for mortality 1.4 and 1.3 and intubation 2.0 and 2.4 significant.	Yes	Overweight (PT 10033307) Body mass index increased (PT 10005897)
Higher risk	Physical inactivity		Cohort (Kaiser 48,4400) consistently inactive severe disease aOR 2.26 and death 2.49. Pending CDC review.	Yes - though may not have been recorded	Exercise lack of (PT 10015650) Housebound (PT 10079226) Immobile (PT 10021417) Immobilization prolonged (PT 10066112)
Higher risk	Pregnancy and recent pregnancy			No - excluded from study	
Higher risk	Primary Immunodeficiencies		Cohort (Turkey = n26, severe disease 38.4% case cases). Pending CDC review.	Yes	Primary immunodeficiency syndromes (HLT 10036700)

© 2023 Theodore DA et al. *JAMA Network Open.*

CDC Category	Condition	Sub-condition	Supporting Literature	Decision to Include	MedDRA term (Version 24.0)
Suggestive higher risk	Sickle cell disease		Cohort (3): (n=4 non-negligible risk); (N=4 children with ACS); (n=83 France - 28% with ACS, worse with increased age, single case reports or small series.	Yes	Sickle cell trait and disorders (HLT 10040651)
Higher risk	Smoking, current and former		OR 8.2 Case Control USA IBM Watson 73 million under SUD category for TUD. Meta-analysis (7; n1576) didn't actually look directly at smoking (Lung disease was RF). Meta-analysis (7; n1592 - COPD. Not smoking). meta-analysis (19; n=11,590 smoker vs never smoker) OR 1.91 severe disease. Combined meta-analysis 2.17 and 2.17 (460,592 patients).	Yes	ex-tobacco user (PT 10065386), Tobacco user (PT 10057581) Passive smoking (PT 10064066) electronic cigarette user (PT 10077387) Tobacco abuse (PT 10043903)
Higher risk	Solid organ or hematopoietic cell transplantation		Multi-center cohort (n=482 SOT) mortality 20.5%, ICU 31%. Cohort - Multi center HSCT n=318 - Mild 49%, critical 15%, survival 68%. No matched systematic review KT vs Nontransplant n=88 KTR; ICU 34.1 vs 15%, death 22 vs 16%.	No - excluded and/or being addressed in another study	
Suggestive higher risk	Substance use disorders		Case control (EHR 73million USA IBM Watson). Increased incidence aOR 8.6, OUD 10.2, TUD 8.2). Worse outcomes death and hospitalization 9.6% & 41% vs 6.6% and 30% general comparison. All subtypes of SUB increased aOR. Significantly less notable aOR for history rather than recent use. Cohort (n=188653 New York) hospitalizations EtOH 6.68aOR but all SUD or other (OUD/CAUD/COUD not significant). Cohort (Korea n=219,961) aOR for hospitalization with SUD 1.32.	Yes	Drug abuse (PT 10013654) Drug dependence (PT 10013663) Drug use disorder (10079381) Drug abuser (PT 10061111) Substance abuser (PT 10067688) Substance abuse (PT 10066169) Substance dependence (PT 10076595) Substance use disorder (PT 10079384)
Suggestive higher risk	Thalassemia		Cross sectional (IRAN B-thal TD and NTD n=23 cases (15 confirmed) - no difference in incidence, increased mortality 26% vs 6.34%. Multiple confounders in Thal group compared to general population. Case series (Italy 11 cases) no difference to general population. two other single patient case reports. Did not include.	Yes	Thalassemic disorders (HLT 10043389)

© 2023 Theodore DA et al. *JAMA Network Open.*

CDC Category	Condition	Sub-condition	Supporting Literature	Decision to Include	MedDRA term (Version 24.0)
Higher risk	Tuberculosis		CDC Systematic Review of 5 cohort studies: suggests underlying TB associated with increased risk/hazard OR of mortality. Single study limited for increased risk of hospitalization OR 1.2 (CI 1.04-1.38)	Yes	Tuberculosis (PT 10044755) Disseminated tuberculosis (PT 10013453) Extrapulmonary tuberculosis (PT 10064445) Intestinal tuberculosis (PT 10075268) Joint tuberculosis (PT 10056367) Lymph node tuberculosis (PT 10025183) Meningitis tuberculous (PT 10027259) Pulmonary tuberculosis (PT 10037440) Pulmonary tuberculoma (PT 10066927) Tuberculous pleurisy (PT 10045104) Tuberculosis of central nervous system (PT 10061391)Tuberculosis of genitourinary system (PT 10044828) Tuberculosis tenosynovitis (PT 10059161)Pericarditis tuberculous 9PT 10055069) Peritoneal tuberculosis (PT 10053583) Bone tuberculosis (PT 10056377) Cutaneous tuberculosis (PT 10011684)
Higher risk	Use of corticosteroids or other immunosuppressive medications		(Meta-analysis (16) Chemotherapy within 30 days OR death 1.85. However severe COVID-19 risk did not increase OR 1.02. Significant heterogeneity. Cohort (n=523 IBD registry) steroids aOR 6.9% severe COVID. Not seen with TNFa. Conflicting: Cohort (n=959 Adult/Peds rheumatic diseases on DMARD Barcelona - Incidence 0.48% of COVID similar to general public and too small for severity (only 11 cases). Case series (n148 northern Italy autoimmune liver disease on steroid or antimetabolites) no difference on self-reported severity of illness compared to general population. Case series (n=7 Madrid, MS with anti-CD20) no increased severity noted.	No - being addressed in another study	

© 2023 Theodore DA et al. *JAMA Network Open.*

eTable 4: Study Endpoints Stratified by Trial. The absolute number of cases for each endpoint, total number of participants available and median follow up time in months is presented. Annual attack rates with 95% confidence intervals were calculated.

Outcome	Study	Number of Endpoints	Total Number of Participants	Median Follow Up Months (Range)	Annual Attack Rate (95% CI)
COVID-19	Pooled Trials	2559	57692	3.8 (0, 11.1)	13.9% (13.3%, 14.4%)
	Moderna	842	15162	4.7 (0, 8.1)	14.6% (13.6%, 15.6%)
	AstraZeneca	370	10793	2.9 (0, 11.1)	12.9% (11.6%, 14.3%)
	Janssen	1191	21890	4 (0, 9.5)	16% (15.1%, 16.9%)
	Novavax	156	9847	2.9 (0, 9.1)	6.6% (5.6%, 7.8%)
Severe COVID-19	Pooled Trials	367	57692	3.9 (0, 11.1)	2% (1.8%, 2.2%)
	Moderna	115	15162	4.8 (0, 8.1)	2% (1.6%, 2.3%)
	AstraZeneca	18	10793	2.9 (0, 11.1)	0.6% (0.4%, 1%)
	Janssen	229	21890	4 (0, 9.5)	3% (2.6%, 3.4%)
	Novavax	5	9847	2.9 (0, 9.1)	0.2% (0.1%, 0.5%)
SARS-COV-2 Infection	Pooled Trials	3774	47492	3.9 (0, 11.1)	24.3% (23.5%, 25.1%)
	Moderna	1333	14164	4.7 (0.6, 8.1)	24.6% (23.3%, 26%)
	AstraZeneca	351	8524	3.2 (1.3, 11.1)	13.9% (12.5%, 15.5%)
	Janssen	1956	19608	3.8 (0, 9.5)	31.1% (29.7%, 32.5%)
	Novavax	134	5196	3.1 (0.9, 5.7)	10.1% (8.5%, 12%)
Subclinical SARS-COV-2 Infection	Pooled Trials	1612	47492	3.9 (0, 11.1)	10.3% (9.8%, 10.9%)
	Moderna	567	14164	4.8 (0.6, 8.1)	10.4% (9.6%, 11.3%)
	AstraZeneca	167	8524	3.2 (1.3, 11.1)	6.6% (5.7%, 7.7%)
	Janssen	792	19608	3.8 (0, 9.5)	12.6% (11.7%, 13.5%)
	Novavax	86	5196	3.1 (1.2, 5.7)	6.5% (5.2%, 8%)

© 2023 Theodore DA et al. *JAMA Network Open.*

eTable 5: Variant Counts from COVID-19 Cases. Sequencing data was available from a subset of cases where sufficient quantity of the virus was available for the four trials. Counts represent absolute numbers. Eleven COVID-19 cases had more than one variant detected; the one based on specimen collected closest to SARS-CoV-2 diagnosis was included.

Variant	Moderna, No.	AZ, No.		Janssen, No.			Novavax, No.
	North America	South America	North America	South Africa	South America	North America	North America
Alpha	--	--	11	2	11	16	38
B.1.1.519	--	--	--	--	--	4	--
B.1.621	--	--	--	--	60	--	--
Beta	--	--	--	70	--	--	1
Delta	--	--	--	12	--	--	--
Epsilon	14	--	18	--	2	19	8
Gamma	1	1	--	--	120	--	3
Iota	--	--	--	--	--	4	3
Lambda	--	19	--	1	46	--	--
Other	406	10	131	2	172	142	54
Other + E484K	6	--	10	3	22	3	4
Reference Sequence	--	--	--	--	21	113	--
Reference Strain	171	--	11	--	--	--	6
Zeta	2	--	--	--	104	1	1

© 2023 Theodore DA et al. *JAMA Network Open.*
