# Supplementary material for: Clinical and Demographic Factors Associated With COVID-19, Severe COVID-19, and SARS-CoV-2 Infection in Adults: A Secondary Cross-Protocol Analysis of 4 Randomized Clinical Trials
Source: JAMA Netw Open. 2023 Jul 13;6(7):e2323349. doi: 10.1001/jamanetworkopen.2023.23349 (PMC10346130; doi:10.1001/jamanetworkopen.2023.23349)
Supplement: Supplement 2. — Members of the COVID-19 Prevention Network (CoVPN) [file jamanetwopen-e2323349-s002.pdf]

Supplemental Online Content: Nonauthor Collaborators
*First name, last name, and suffix (if applicable) are required and will appear in PubMed.

*Group Name(s): COVID-19 Prevention Network (CoVPN)							
***First Name and Middle Initial(s)**	***Last Name**	***Suffix (eg, Jr, III)**	Academic Degrees	Institution	Location (city, state/province, country)	Role or Contribution, eg, chair, principal investigator	Group (if more than 1 Group listed in the byline) and/or Subgroup (eg, Steering Committee)
Atoya	Adams		MD, MBA				COVID-19 Prevention Network
Eric	Miller						COVID-19 Prevention Network
Bruce G.	Rankin		DO				COVID-19 Prevention Network
Steven	Shinn		MD				COVID-19 Prevention Network
Marshall	Nash		MD				COVID-19 Prevention Network
Sinikka L.	Green		MD				COVID-19 Prevention Network
Colleen	Jacobsen						COVID-19 Prevention Network
Jayasree	Krishnankutty						COVID-19 Prevention Network
Sikhongi	Phungwayo						COVID-19 Prevention Network
Richard M.	Glover	II	MD				COVID-19 Prevention Network
Stacy	Slechta		DO				COVID-19 Prevention Network
Troy	Holdeman		MD				COVID-19 Prevention Network
Robyn	Hartvickson		MD				COVID-19 Prevention Network
Amber	Grant		APRN				COVID-19 Prevention Network
Terry L.	Poling		MD				COVID-19 Prevention Network
Terry D.	Klein		MD				COVID-19 Prevention Network
Thomas C.	Klein		MD				COVID-19 Prevention Network
Tracy R.	Klein		MD				COVID-19 Prevention Network
William B.	Smith		MD				COVID-19 Prevention Network
Richard L.	Gibson		MD				COVID-19 Prevention Network
Jennifer	Winbigler		MD				COVID-19 Prevention Network
Elizabeth	Parker		PA				COVID-19 Prevention Network
Priyantha N.	Wijewardane		MD				COVID-19 Prevention Network
Eric	Bravo		MD				COVID-19 Prevention Network
Jeffrey	Thessing		MD				COVID-19 Prevention Network
Michelle	Maxwell		APRN				COVID-19 Prevention Network
Amanda	Horn		APRN				COVID-19 Prevention Network
Catherine	Mary Healy		MD				COVID-19 Prevention Network
Christine	Akamine		MD				COVID-19 Prevention Network
Laurence	Chu		MD				COVID-19 Prevention Network
R. Michelle	Chouteau		MD				COVID-19 Prevention Network
Michael J.	Cotugno		MD				COVID-19 Prevention Network
George H.	Bauer	Jr.	MD				COVID-19 Prevention Network
Greg	Hachigian		MD				COVID-19 Prevention Network
Masaru	Oshita		MD				COVID-19 Prevention Network
Michael	Cancilla		NP				COVID-19 Prevention Network
Kristen	Kiersey		NP				COVID-19 Prevention Network
William	Seger		MD				COVID-19 Prevention Network
Mohammed	Antwi						COVID-19 Prevention Network
Allison	Green						COVID-19 Prevention Network

*First Name and Middle Initial(s)	*Last Name	*Suffix (eg, Jr, III)	Academic Degrees	Institution	Location (city, state/province, country)	Role or Contribution, eg, chair, principal investigator	Group (if more than 1 Group listed in the byline) and/or Subgroup (eg, Steering Committee)
Anthony	Kim						COVID-19 Prevention Network
Michael	Desjardins		MD				COVID-19 Prevention Network
Jennifer A.	Johnson		MD				COVID-19 Prevention Network
Amy	Sherman		MD				COVID-19 Prevention Network
Stephen R.	Walsh		MD				COVID-19 Prevention Network
Judith	Borger		DO				COVID-19 Prevention Network
Nafisa	Saleem		MD				COVID-19 Prevention Network
Joel	Solis		MD				COVID-19 Prevention Network
Martha	Carmen Medina		PA-C				COVID-19 Prevention Network
Westly	Keating		PA-C				COVID-19 Prevention Network
Edgar	Garcia		PA-C				COVID-19 Prevention Network
Cynthia	Bueno		PA-C				COVID-19 Prevention Network
Nathan	Segall		MD				COVID-19 Prevention Network
Douglas S.	Denham		DO				COVID-19 Prevention Network
Thomas	Weiss		MD				COVID-19 Prevention Network
Ayoade	Avworo		DNP				COVID-19 Prevention Network
Parke	Hedges		MD				COVID-19 Prevention Network
Cynthia	Becher Strout		MD				COVID-19 Prevention Network
Rica	Santiago						COVID-19 Prevention Network
Yvonne	Davis						COVID-19 Prevention Network
Patty	Howenstine						COVID-19 Prevention Network
Alison	Bondell						COVID-19 Prevention Network
Kristin	Marks		MS, MD				COVID-19 Prevention Network
Tina	Wang		MD				COVID-19 Prevention Network
Timothy	Wilkin		MD, MPH				COVID-19 Prevention Network
Mary	Vogler		MD				COVID-19 Prevention Network
Carrie	Johnston		MD, MS				COVID-19 Prevention Network
Michele P.	Andrasik		PhD				COVID-19 Prevention Network
Jessica G.	Andriesen		PhD				COVID-19 Prevention Network
Gail	Broder						COVID-19 Prevention Network
Niles	Eaton						COVID-19 Prevention Network
Huub G.	Gelderblom		MD, PhD, MPH				COVID-19 Prevention Network
Rachael	McClennen						COVID-19 Prevention Network
Nelson	Michael						COVID-19 Prevention Network
Merlin	Robb		MD				COVID-19 Prevention Network
Carrie	Sopher						COVID-19 Prevention Network
Vicki E.	Miller		MD, MPH				COVID-19 Prevention Network
Fredric	Santiago		MD				COVID-19 Prevention Network
Blanca	Gomez		FNP-C				COVID-19 Prevention Network
Insiya	Valika		PA-C				COVID-19 Prevention Network
Amy	Starr		FNP-C				COVID-19 Prevention Network
Valeria D.	Cantos		MD				COVID-19 Prevention Network

*First Name and Middle Initial(s)	*Last Name	*Suffix (eg, Jr, III)	Academic Degrees	Institution	Location (city, state/province, country)	Role or Contribution, eg, chair, principal investigator	Group (if more than 1 Group listed in the byline) and/or Subgroup (eg, Steering Committee)
Sheetal	Kandiah		MD, MPH				COVID-19 Prevention Network
Carlos	del Rio		MD				COVID-19 Prevention Network
Nadine	Rouphael		MD				COVID-19 Prevention Network
Srilatha	Edupuganti						COVID-19 Prevention Network
Evan J.	Anderson		MD				COVID-19 Prevention Network
Andres	Camacho-Gonzalez		MD				COVID-19 Prevention Network
Satoshi	Kamidani		MD				COVID-19 Prevention Network
Christiana A.	Rostad		MD				COVID-19 Prevention Network
Meghan	Teherani		MD				COVID-19 Prevention Network
David J.	Diemert		MD				COVID-19 Prevention Network
Elissa	Malkin						COVID-19 Prevention Network
Marc	Siegel						COVID-19 Prevention Network
Afsoon	Roberts						COVID-19 Prevention Network
Gary	Simon						COVID-19 Prevention Network
Bindu	Balani		MD				COVID-19 Prevention Network
Carolene	Stephenson						COVID-19 Prevention Network
Steven	Sperber						COVID-19 Prevention Network
Cristina	Cicogna						COVID-19 Prevention Network
Marcus J.	Zervos		MD				COVID-19 Prevention Network
Paul	Kilgore		MD, MPH				COVID-19 Prevention Network
Mayur	Ramesh		MD				COVID-19 Prevention Network
Erica	Herc		MD				COVID-19 Prevention Network
Kate	Zenlea		MPH				COVID-19 Prevention Network
Abram	Burgher		MD				COVID-19 Prevention Network
Ann M.	Milliken						COVID-19 Prevention Network
Joseph D.	Davis		MD				COVID-19 Prevention Network
Brendan	Levy						COVID-19 Prevention Network
Sandra	Kelman						COVID-19 Prevention Network
Matthew W.	Doust		MD				COVID-19 Prevention Network
Denise	Sample						COVID-19 Prevention Network
Sandra	Erickson						COVID-19 Prevention Network
Shane G.	Christensen		MD				COVID-19 Prevention Network
Christopher	Matich						COVID-19 Prevention Network
James	Longe						COVID-19 Prevention Network
John	Witbeck						COVID-19 Prevention Network
James T.	Peterson		MD				COVID-19 Prevention Network
Alexander	Clark						COVID-19 Prevention Network
Gerald	Kelty						COVID-19 Prevention Network
Issac	Pena-Renteria						COVID-19 Prevention Network
Michael J.	Koren		MD				COVID-19 Prevention Network
Darlene	Bartilucci		MD				COVID-19 Prevention Network
Alpa	Patel		MD				COVID-19 Prevention Network

Supplemental Online Content: Nonauthor Collaborators
*First name, last name, and suffix (if applicable) are required and will appear in PubMed.

*First Name and Middle Initial(s)	*Last Name	*Suffix (eg, Jr, III)	Academic Degrees	Institution	Location (city, state/province, country)	Role or Contribution, eg, chair, principal investigator	Group (if more than 1 Group listed in the byline) and/or Subgroup (eg, Steering Committee)
Carolyn	Tran		MD				COVID-19 Prevention Network
Christina	Kennelly		MD				COVID-19 Prevention Network
Robert	Brownlee						COVID-19 Prevention Network
Jacob	Coleman						COVID-19 Prevention Network
Hala	Webster						COVID-19 Prevention Network
Carlos A.	Fierro		MD				COVID-19 Prevention Network
Natalia	Leistner						COVID-19 Prevention Network
Amy	Thompson						COVID-19 Prevention Network
Celia	Gonzalez						COVID-19 Prevention Network
Lisa A	Jackson		MD, MPH				COVID-19 Prevention Network
Janice	Suyehira		MD				COVID-19 Prevention Network
Milton	Haber		MD				COVID-19 Prevention Network
Maria M.	Regalado		MD				COVID-19 Prevention Network
Veronica	Procasky		RN, JD				COVID-19 Prevention Network
Alisha	Lutat						COVID-19 Prevention Network
Carl P.	Griffin		MD				COVID-19 Prevention Network
Ripley R.	Hollister		MD				COVID-19 Prevention Network
Jeremy	Brown		DO				COVID-19 Prevention Network
Melody	Ronk		PA-C				COVID-19 Prevention Network
Wayne L.	Harper		MD				COVID-19 Prevention Network
Lisa	Cohen		DO				COVID-19 Prevention Network
Lynn	Eckert		PA-C				COVID-19 Prevention Network
Matthew	Hong		MD				COVID-19 Prevention Network
Rambod	Rouhbakhsh		MD, MBA				COVID-19 Prevention Network
Elizabeth	Danford		MD				COVID-19 Prevention Network
John	Johnson		MD				COVID-19 Prevention Network
Richard	Calderone		MD				COVID-19 Prevention Network
Shishir K.	Khetan		MD				COVID-19 Prevention Network
Oyebisi	Olanrewaju		AC-CRNP				COVID-19 Prevention Network
Nan	Zhai		NP-C				COVID-19 Prevention Network
Kimberly	Nieves		AC-CRNP				COVID-19 Prevention Network
Allison	O'Brien		AC-CRNP				COVID-19 Prevention Network
Paul S	Bradley		MD				COVID-19 Prevention Network
Amanda	Lilienthal		MSN NP-C				COVID-19 Prevention Network
Jim	Callis		PA-C				COVID-19 Prevention Network
Adam B	Brosz		MD				COVID-19 Prevention Network
Andrea	Clement		PA				COVID-19 Prevention Network
Whitney	West		APRN				COVID-19 Prevention Network
Luke	Friesen		PA				COVID-19 Prevention Network
Paul	Cramer		APRN				COVID-19 Prevention Network
Frank S	Eder		MD				COVID-19 Prevention Network
Ryan	Little		FNP				COVID-19 Prevention Network

*First name, last name, and suffix (if applicable) are required and will appear in PubMed.

*First Name and Middle Initial(s)	*Last Name	*Suffix (eg, Jr, III)	Academic Degrees	Institution	Location (city, state/province, country)	Role or Contribution, eg, chair, principal investigator	Group (if more than 1 Group listed in the byline) and/or Subgroup (eg, Steering Committee)
Victoria	Engler		FNP				COVID-19 Prevention Network
Heather	Rattenbury-Shaw		DO				COVID-19 Prevention Network
David J.	Ensz		MD				COVID-19 Prevention Network
Allie	Oplinger						COVID-19 Prevention Network
Brandon J.	Essink		MD				COVID-19 Prevention Network
Jay	Meyer		MD				COVID-19 Prevention Network
Frederick	Raiser	III	MD				COVID-19 Prevention Network
Kimberly	Mueller		APRN				COVID-19 Prevention Network
Keith W.	Vrbicky		MD				COVID-19 Prevention Network
Charles	Harper		MD				COVID-19 Prevention Network
Chelsie	Nutsch		MD				COVID-19 Prevention Network
Wendell	Lewis	III	MD				COVID-19 Prevention Network
Cathy	Laflan		MD				COVID-19 Prevention Network
Jordan L.	Whatley		MD				COVID-19 Prevention Network
Nicole	Harrell		MD				COVID-19 Prevention Network
Amie	Shannon		MD				COVID-19 Prevention Network
Crystal	Rowell		APRN, FNP-C				COVID-19 Prevention Network
Christopher	Dedon		APRN, FNP-C				COVID-19 Prevention Network
Mamodikoe	Makhene		MD, MPH				COVID-19 Prevention Network
Gregory M.	Gottschlich		MD				COVID-19 Prevention Network
Kate	Harden		PA-C				COVID-19 Prevention Network
Melissa	Gottschlich		PA-C				COVID-19 Prevention Network
Mary	Smith		MSN, FNP-C				COVID-19 Prevention Network
Richard	Powell		MD				COVID-19 Prevention Network
Murray A.	Kimmel		DO				COVID-19 Prevention Network
Simmy	Pinto		MD				COVID-19 Prevention Network
Timothy P.	Vachris		MD				COVID-19 Prevention Network
Mark	Hutchens		MD				COVID-19 Prevention Network
Stephen	Daniels		DO				COVID-19 Prevention Network
Margaret	Wells		MD				COVID-19 Prevention Network
Mimi	Van Der Leden		MD, PhD				COVID-19 Prevention Network
Peta-Gay	Jackson-Booth		MD				COVID-19 Prevention Network
Mira	Baron		MD				COVID-19 Prevention Network
Pamela	Kane		DO				COVID-19 Prevention Network
Shannen	Seversen		PA-C				COVID-19 Prevention Network
Mara	Kryvicky		PA-C				COVID-19 Prevention Network
Julia	Lord		PA-C				COVID-19 Prevention Network
Jamshid	Saleh		MD				COVID-19 Prevention Network
Matthew	Miles						COVID-19 Prevention Network
Rafael	Lupercio						COVID-19 Prevention Network
John W.	McGettigan	Jr.	MD				COVID-19 Prevention Network
Walter	Patton		MD				COVID-19 Prevention Network

Supplemental Online Content: Nonauthor Collaborators
*First name, last name, and suffix (if applicable) are required and will appear in PubMed.

***First Name and Middle Initial(s)**	***Last Name**	***Suffix (eg, Jr, III)**	Academic Degrees	Institution	Location (city, state/province, country)	Role or Contribution, eg, chair, principal investigator	Group (if more than 1 Group listed in the byline) and/or Subgroup (eg, Steering Committee)
Riemke	Brakema		MD				COVID-19 Prevention Network
Karin	Choquette		MSN, ABNP-C				COVID-19 Prevention Network
Jonlyn	McGettigan		MSN, RN				COVID-19 Prevention Network
Judith L.	Kirstein		MD				COVID-19 Prevention Network
Marcia	Bernard		NP				COVID-19 Prevention Network
Mary Beth	Manning		MD				COVID-19 Prevention Network
Joan	Rothenberg		MD				COVID-19 Prevention Network
Toby	Briskin		MD				COVID-19 Prevention Network
Denise	Roadman		PAC				COVID-19 Prevention Network
Sharita	Tedder-Edwards		FNP				COVID-19 Prevention Network
Howard I.	Schwartz		MD				COVID-19 Prevention Network
Surisday	Mederos						COVID-19 Prevention Network
Shobha	Swaminathan		MD				COVID-19 Prevention Network
Amesika	Nyaku		MD, MS				COVID-19 Prevention Network
Tilly	Varughese		MD				COVID-19 Prevention Network
Michelle	DallaPiazza		MD				COVID-19 Prevention Network
Sharon E.	Frey		MD				COVID-19 Prevention Network
Irene	Graham		MD				COVID-19 Prevention Network
Getahun	Abate		MD, PhD, MSc				COVID-19 Prevention Network
Daniel	Hoft		MD, PhD				COVID-19 Prevention Network
Leland N.	Allen	III	MD				COVID-19 Prevention Network
Leslie A.	Edwards		MSN, CRNP				COVID-19 Prevention Network
William S.	Davis	Jr.	MS, PA-C				COVID-19 Prevention Network
Jessica M.	Mena		PA				COVID-19 Prevention Network
Mark E.	Kutner		MD				COVID-19 Prevention Network
Jorge	Caso		MD, CPI				COVID-19 Prevention Network
Maria	Hernandez Moran		APRN				COVID-19 Prevention Network
Marianela	Carvajal		APRN				COVID-19 Prevention Network
Janet	Mendez		APRN				COVID-19 Prevention Network
Larkin T.	Wadsworth	III	MD				COVID-19 Prevention Network
Michael R.	Adams		MD				COVID-19 Prevention Network
Leslie	Iverson		PA				COVID-19 Prevention Network
Joseph L.	Newberg		MD				COVID-19 Prevention Network
Laura	Pearlman		MS, MD, MBA				COVID-19 Prevention Network
Paul J.	Nugent		DO				COVID-19 Prevention Network
Michele D.	Reynolds		MD				COVID-19 Prevention Network
Jennifer	Bashour		MD				COVID-19 Prevention Network
Robert	Schmidt		MD				COVID-19 Prevention Network
Neil P.	Sheth		MD				COVID-19 Prevention Network
Kenneth	Steil		DO				COVID-19 Prevention Network
Ramy J.	Toma		MD				COVID-19 Prevention Network
William	Kirby		MD				COVID-19 Prevention Network

***First Name and Middle Initial(s)**	***Last Name**	***Suffix (eg, Jr, III)**	Academic Degrees	Institution	Location (city, state/province, country)	Role or Contribution, eg, chair, principal investigator	Group (if more than 1 Group listed in the byline) and/or Subgroup (eg, Steering Committee)
Pink	Folmar		MD				COVID-19 Prevention Network
Samantha	Williams		NP				COVID-19 Prevention Network
Paul	Pickrell		MD				COVID-19 Prevention Network
Stefanie	Mott		FNP-C				COVID-19 Prevention Network
Carol Ann	Linebarger		MD				COVID-19 Prevention Network
Hussain	Malbari		MD				COVID-19 Prevention Network
David	Pampe		MD				COVID-19 Prevention Network
Veronica G.	Fragoso		MD				COVID-19 Prevention Network
Lisa	Holloway		MD				COVID-19 Prevention Network
Cecilia	McKeown-Bragas		MD				COVID-19 Prevention Network
Teresa	Becker		MD				COVID-19 Prevention Network
Barton G.	Williams		MD				COVID-19 Prevention Network
William H.	Jones		MD				COVID-19 Prevention Network
Jesse L.	Clark		MD				COVID-19 Prevention Network
Steven	Shoptaw		PhD				COVID-19 Prevention Network
Michele	Vertucci		PA, NP				COVID-19 Prevention Network
Will	Hernandez		NP				COVID-19 Prevention Network
Stephen A.	Spector		MD				COVID-19 Prevention Network
Amaran	Moodley		MD				COVID-19 Prevention Network
Jill	Blumenthal		MD				COVID-19 Prevention Network
Lisa	Stangl		NP				COVID-19 Prevention Network
Karen	Deutsch		NP				COVID-19 Prevention Network
Kathleen M.	Mullane		DO, PharmD				COVID-19 Prevention Network
David	Pitrak		MD				COVID-19 Prevention Network
Cheryl	Nuss		FNP				COVID-19 Prevention Network
Judy	Pi		PharmD				COVID-19 Prevention Network
Carl	Fichtenbaum		MD				COVID-19 Prevention Network
Margaret	Powers-Fletcher		PhD				COVID-19 Prevention Network
Michelle	Saemann		RN				COVID-19 Prevention Network
Sharon	Kohrs		RN				COVID-19 Prevention Network
Thomas B.	Campbell		MD				COVID-19 Prevention Network
Andrew	Lauria						COVID-19 Prevention Network
Jose C.	Mancilla						COVID-19 Prevention Network
Hillary	Dunlevy						COVID-19 Prevention Network
Richard M.	Novak		MD				COVID-19 Prevention Network
Andrea	Wendrow						COVID-19 Prevention Network
Scott	Borgetti						COVID-19 Prevention Network
Ben	Ladner						COVID-19 Prevention Network
Matthew	Laurens		MD				COVID-19 Prevention Network
Lisa	Chrisley						COVID-19 Prevention Network
Cheryl	Young						COVID-19 Prevention Network
Susanne	Doblecki-Lewis		MD				COVID-19 Prevention Network

*First name, last name, and suffix (if applicable) are required and will appear in PubMed.

*First Name and Middle Initial(s)	*Last Name	*Suffix (eg, Jr, III)	Academic Degrees	Institution	Location (city, state/province, country)	Role or Contribution, eg, chair, principal investigator	Group (if more than 1 Group listed in the byline) and/or Subgroup (eg, Steering Committee)
Maria L.	Alcaide						COVID-19 Prevention Network
Jose	Gonzales-Zamora						COVID-19 Prevention Network
Stephen	Morris						COVID-19 Prevention Network
David	Wohl		MD				COVID-19 Prevention Network
Joseph	Eron	Jr.	MD				COVID-19 Prevention Network
Ian	Frank		MD				COVID-19 Prevention Network
Debora	Dunbar						COVID-19 Prevention Network
David	Metzger						COVID-19 Prevention Network
Florence	Momplaisir						COVID-19 Prevention Network
Judith	Martin		MD				COVID-19 Prevention Network
Alejandro	Hoberman		MD				COVID-19 Prevention Network
Timothy	Shope		MD, MPH				COVID-19 Prevention Network
Gysella	Muniz		MD				COVID-19 Prevention Network
Richard	Rupp		MD				COVID-19 Prevention Network
Amber	Stanford		PA-C				COVID-19 Prevention Network
Megan	Berman		MD				COVID-19 Prevention Network
Laura	Porterfield		MD				COVID-19 Prevention Network
Michael	Lewis		MD				COVID-19 Prevention Network
Elham	Ghadishah						COVID-19 Prevention Network
Joseph	Yusin						COVID-19 Prevention Network
Mai	Pham						COVID-19 Prevention Network
Clarence B.	Creech	II	MD				COVID-19 Prevention Network
Shannon	Walker		MD				COVID-19 Prevention Network
Stephanie	Rolsma		MD, PhD				COVID-19 Prevention Network
Robert	Samuels						COVID-19 Prevention Network
Isaac	Thomsen		MD				COVID-19 Prevention Network
Spyros A.	Kalams		MD				COVID-19 Prevention Network
Greg	Wilson		MD				COVID-19 Prevention Network
Gregg H.	Lucksinger		MD				COVID-19 Prevention Network
Kevin	Parks		MD				COVID-19 Prevention Network
Ryan	Israelsen		MD				COVID-19 Prevention Network
Jaleh	Ostovar		FNP-C				COVID-19 Prevention Network
Kary	Kelly		FNP-C				COVID-19 Prevention Network
Jeffrey S.	Overcash		MD				COVID-19 Prevention Network
Hanh	Chu						COVID-19 Prevention Network
Kia	Lee						COVID-19 Prevention Network
Luis I.	De La Cruz		MD				COVID-19 Prevention Network
Steve	Clemons						COVID-19 Prevention Network
Elizabeth	Everette						COVID-19 Prevention Network
Suzanna	Studdard						COVID-19 Prevention Network
Gowdhami	Mohan		MD				COVID-19 Prevention Network
Stefanie	Tyson						COVID-19 Prevention Network

Supplemental Online Content: Nonauthor Collaborators
*First name, last name, and suffix (if applicable) are required and will appear in PubMed.

*First Name and Middle Initial(s)	*Last Name	*Suffix (eg, Jr, III)	Academic Degrees	Institution	Location (city, state/province, country)	Role or Contribution, eg, chair, principal investigator	Group (if more than 1 Group listed in the byline) and/or Subgroup (eg, Steering Committee)
Alyssa-Kay	Peay						COVID-19 Prevention Network
Danyel	Johnson						COVID-19 Prevention Network
Gregory J.	Feldman		MD				COVID-19 Prevention Network
May-Yin	Suen						COVID-19 Prevention Network
Jacqueline	Muenzner						COVID-19 Prevention Network
Joseph	Boscia						COVID-19 Prevention Network
Farhan	Siddiqui						COVID-19 Prevention Network
John	Sanders		MD, PhD				COVID-19 Prevention Network
James	Peacock		MD				COVID-19 Prevention Network
Julio	Nasim		MD				COVID-19 Prevention Network
Michael L.	Levin		MD				COVID-19 Prevention Network
Julie	Hussey		MSN, APRN, FNP-C				COVID-19 Prevention Network
Marcy	Kulic		MD				COVID-19 Prevention Network
Mark M.	McKenzie		MD				COVID-19 Prevention Network
Teresa	Deese						COVID-19 Prevention Network
Erica	Osmundsen						COVID-19 Prevention Network
Christy	Sweet						COVID-19 Prevention Network
Valentine M.	Ebuh		MD, MA, MSc				COVID-19 Prevention Network
Elwaleed	Elnagar		MD				COVID-19 Prevention Network
Georgette	Ebuh		DNP, APRN, FNP-C				COVID-19 Prevention Network
Genevieve	Iwuala		FNP				COVID-19 Prevention Network
Laurie J.	Han-Conrad		MD				COVID-19 Prevention Network
Todd	Simmons		MD				COVID-19 Prevention Network
Denis	Tarakjian		MD				COVID-19 Prevention Network
Jeremy	Ackermann						COVID-19 Prevention Network
Mark S.	Adams						COVID-19 Prevention Network
José O.	Alemán						COVID-19 Prevention Network
Mohamed S.	Al-Ibrahim						COVID-19 Prevention Network
David R.	Andes						COVID-19 Prevention Network
Jeb	Andrews						COVID-19 Prevention Network
Roberto C.	Arduino						COVID-19 Prevention Network
Martín	Bäcker						COVID-19 Prevention Network
Diana	Badillo						COVID-19 Prevention Network
Emma	Bainbridge						COVID-19 Prevention Network
Teresa A.	Batteiger						COVID-19 Prevention Network
Jose A.	Bazan						COVID-19 Prevention Network
Roger J.	Bedimo						COVID-19 Prevention Network
Jorge A.	Benitez						COVID-19 Prevention Network
Annette R.	Bennett						COVID-19 Prevention Network
David I.	Bernstein						COVID-19 Prevention Network
Kristin	Bialobok						COVID-19 Prevention Network
Rebecca	Boas						COVID-19 Prevention Network

Supplemental Online Content: Nonauthor Collaborators
*First name, last name, and suffix (if applicable) are required and will appear in PubMed.

***First Name and Middle Initial(s)**	***Last Name**	***Suffix (eg, Jr, III)**	Academic Degrees	Institution	Location (city, state/province, country)	Role or Contribution, eg, chair, principal investigator	Group (if more than 1 Group listed in the byline) and/or Subgroup (eg, Steering Committee)
Judith	Brady						COVID-19 Prevention Network
Cynthia	Brown						COVID-19 Prevention Network
Catherine A.	Bunce						COVID-19 Prevention Network
Robert S.	Call						COVID-19 Prevention Network
Wesley	Campbell						COVID-19 Prevention Network
Ellie	Carmody						COVID-19 Prevention Network
Christopher	Carpenter						COVID-19 Prevention Network
Steven E.	Carsons						COVID-19 Prevention Network
Marvin	Castellon						COVID-19 Prevention Network
Mario	Castro						COVID-19 Prevention Network
Hannah	Catan						COVID-19 Prevention Network
Jennifer	Chang						COVID-19 Prevention Network
Mouna G.	Chebib						COVID-19 Prevention Network
Corey M.	Chen						COVID-19 Prevention Network
Margaret	Cheng						COVID-19 Prevention Network
Brian D. W.	Chow						COVID-19 Prevention Network
Annie	Ciambruschini						COVID-19 Prevention Network
Joseph P.	Connor						COVID-19 Prevention Network
James H.	Conway						COVID-19 Prevention Network
Maureen	Cooney						COVID-19 Prevention Network
Marcel	Curlin						COVID-19 Prevention Network
Claudia	De La Matta Rodriguez						COVID-19 Prevention Network
Jon F.	Dedon						COVID-19 Prevention Network
Emily	Degan						COVID-19 Prevention Network
Michelle	Dickey						COVID-19 Prevention Network
Craig	Dietz						COVID-19 Prevention Network
Jennifer L	Dong						COVID-19 Prevention Network
Brenda	Dorcely						COVID-19 Prevention Network
Michael P.	Dube						COVID-19 Prevention Network
Carmel B.	Dyer						COVID-19 Prevention Network
Benjamin	Eckhardt						COVID-19 Prevention Network
Edward	Ellerbeck						COVID-19 Prevention Network
Evan C.	Ewers						COVID-19 Prevention Network
Amy	Falk						COVID-19 Prevention Network
Brittany	Feijoo						COVID-19 Prevention Network
Uriel R.	Felsen						COVID-19 Prevention Network
Tom	Fiel						COVID-19 Prevention Network
David	Fitz-Patrick						COVID-19 Prevention Network
Charles M.	Fogarty						COVID-19 Prevention Network
Stacy	Ford						COVID-19 Prevention Network
Lina M.	Forero						COVID-19 Prevention Network
Elizabeth	Formentini						COVID-19 Prevention Network

Supplemental Online Content: Nonauthor Collaborators
*First name, last name, and suffix (if applicable) are required and will appear in PubMed.

***First Name and Middle Initial(s)**	***Last Name**	***Suffix (eg, Jr, III)**	Academic Degrees	Institution	Location (city, state/province, country)	Role or Contribution, eg, chair, principal investigator	Group (if more than 1 Group listed in the byline) and/or Subgroup (eg, Steering Committee)
Doris	Franco-Vitteri						COVID-19 Prevention Network
Robert W.	Frenck	Jr.					COVID-19 Prevention Network
Elie	Gharib						COVID-19 Prevention Network
Suzanne	Gharib						COVID-19 Prevention Network
Rola G.	Rucker						COVID-19 Prevention Network
James N.	Goldenberg						COVID-19 Prevention Network
Luis H.	González						COVID-19 Prevention Network
Brett	Gray						COVID-19 Prevention Network
Rusty	Greene						COVID-19 Prevention Network
Robert M.	Grossberg						COVID-19 Prevention Network
Juan V.	Guanira-Carranza						COVID-19 Prevention Network
Alfredo	Gilberto Guerreros Benavides						COVID-19 Prevention Network
Clint C.	Guillory						COVID-19 Prevention Network
Shauna H.	Gunaratne						COVID-19 Prevention Network
David	Halpert						COVID-19 Prevention Network
Holli	Hamilton						COVID-19 Prevention Network
William R.	Hartman						COVID-19 Prevention Network
Sheryl L.	Henderson						COVID-19 Prevention Network
Ramin	Herati						COVID-19 Prevention Network
Laura	Hernandez Guarin						COVID-19 Prevention Network
Robin	Hilder						COVID-19 Prevention Network
Ken	Ho						COVID-19 Prevention Network
Leila	Hojat						COVID-19 Prevention Network
Sybil G.	Hosek						COVID-19 Prevention Network
Jeffrey M.	Jacobson						COVID-19 Prevention Network
Melanie	Jay						COVID-19 Prevention Network
Diane H.	Johnson						COVID-19 Prevention Network
Kathleen S.	Jones						COVID-19 Prevention Network
Edward C.	Jones-López						COVID-19 Prevention Network
Jessica E.	Justman						COVID-19 Prevention Network
Scott	Kahney						COVID-19 Prevention Network
Lois	Katz						COVID-19 Prevention Network
Melinda	Katz						COVID-19 Prevention Network
Daniel	Kaul						COVID-19 Prevention Network
Michael C.	Keefer						COVID-19 Prevention Network
Ashley	Kennedy						COVID-19 Prevention Network
Jennifer	Knishinsky						COVID-19 Prevention Network
Laura	Kogelman						COVID-19 Prevention Network
Susan L.	Koletar						COVID-19 Prevention Network
Angelica	Kottkamp						COVID-19 Prevention Network
Maryrose	Laguio-Vila						COVID-19 Prevention Network
Raphael J.	Landovitz						COVID-19 Prevention Network

Supplemental Online Content: Nonauthor Collaborators
*First name, last name, and suffix (if applicable) are required and will appear in PubMed.

***First Name and Middle Initial(s)**	***Last Name**	***Suffix (eg, Jr, III)**	Academic Degrees	Institution	Location (city, state/province, country)	Role or Contribution, eg, chair, principal investigator	Group (if more than 1 Group listed in the byline) and/or Subgroup (eg, Steering Committee)
Jessica L.	Lee						COVID-19 Prevention Network
Albert	Liu						COVID-19 Prevention Network
Eneyda	Giuvanela Llerena Zegarra						COVID-19 Prevention Network
Anna S.	Lok						COVID-19 Prevention Network
James	Lovell						COVID-19 Prevention Network
Ronald	Lubelchek						COVID-19 Prevention Network
John	Lucaj						COVID-19 Prevention Network
Gary	Luckasen						COVID-19 Prevention Network
Annie	Luetkemeyer						COVID-19 Prevention Network
Njira	Lucia Lugogo						COVID-19 Prevention Network
Janine	Maenza						COVID-19 Prevention Network
Carlos	Malvestutto						COVID-19 Prevention Network
Monica	Mauri						COVID-19 Prevention Network
Ryan C.	Maves						COVID-19 Prevention Network
Kenneth H.	Mayer						COVID-19 Prevention Network
Michael J.	McCartney						COVID-19 Prevention Network
Margaret E.	McCort						COVID-19 Prevention Network
M. Juliana	McElrath						COVID-19 Prevention Network
Meredith	McNairy						COVID-19 Prevention Network
Fernando L.	Merino						COVID-19 Prevention Network
Eric A.	Meyerowitz						COVID-19 Prevention Network
Carol L.	Mitchell						COVID-19 Prevention Network
Cynthia L.	Monaco						COVID-19 Prevention Network
Sauda	Muhammad						COVID-19 Prevention Network
Sigridh	Muñoz-Gómez						COVID-19 Prevention Network
Sonal	Munsiff						COVID-19 Prevention Network
Paul	Nee						COVID-19 Prevention Network
Nicole L.	Nollen						COVID-19 Prevention Network
Asif	Noor						COVID-19 Prevention Network
Claudio	Nuñez Lagos						COVID-19 Prevention Network
Jason F.	Okulicz						COVID-19 Prevention Network
Patrick A.	Oliver						COVID-19 Prevention Network
Jessica	Ortega						COVID-19 Prevention Network
Steven	Palmer						COVID-19 Prevention Network
Lalitha	Parameswaran						COVID-19 Prevention Network
Purvi	Parikh						COVID-19 Prevention Network
Susan	Parker						COVID-19 Prevention Network
Reza	Parungao						COVID-19 Prevention Network
Juana R.	Pavie						COVID-19 Prevention Network
Rebecca P.	Madan						COVID-19 Prevention Network
Henry	Peralta						COVID-19 Prevention Network
Jennifer	Petts						COVID-19 Prevention Network

Supplemental Online Content: Nonauthor Collaborators
*First name, last name, and suffix (if applicable) are required and will appear in PubMed.

*First Name and Middle Initial(s)	*Last Name	*Suffix (eg, Jr, III)	Academic Degrees	Institution	Location (city, state/province, country)	Role or Contribution, eg, chair, principal investigator	Group (if more than 1 Group listed in the byline) and/or Subgroup (eg, Steering Committee)
Kristen K.	Pierce						COVID-19 Prevention Network
E. Javier	Pretell Alva						COVID-19 Prevention Network
Lawrence J.	Purpura						COVID-19 Prevention Network
Vanessa	Raabe						COVID-19 Prevention Network
Sergio E.	Recuenco						COVID-19 Prevention Network
Tamara	Richards						COVID-19 Prevention Network
Sharon A.	Riddler						COVID-19 Prevention Network
Barbara	Rizzardi						COVID-19 Prevention Network
Rachel	Rokser						COVID-19 Prevention Network
Charlotte-Paige	Rolle						COVID-19 Prevention Network
Adam	Rosen						COVID-19 Prevention Network
Jeffrey	Rosen						COVID-19 Prevention Network
Lena R.	Freese						COVID-19 Prevention Network
María E.	Santolaya						COVID-19 Prevention Network
Linda M.	Schipani						COVID-19 Prevention Network
Adam	Schwartz						COVID-19 Prevention Network
Tiffany	Schwasinger-Schmidt						COVID-19 Prevention Network
Hyman	Scott						COVID-19 Prevention Network
Beverly E.	Sha						COVID-19 Prevention Network
Shivanjali	Shankaran						COVID-19 Prevention Network
Adrienne E.	Shapiro						COVID-19 Prevention Network
Stephan C.	Sharp						COVID-19 Prevention Network
Bo	Shopsin						COVID-19 Prevention Network
Matthew D.	Sims						COVID-19 Prevention Network
Stephanie	Skipper						COVID-19 Prevention Network
Derek M.	Smith						COVID-19 Prevention Network
Michael J.	Smith						COVID-19 Prevention Network
M. Mahdee	Sobhanie						COVID-19 Prevention Network
Brit	Sovic						COVID-19 Prevention Network
Stephanie	Sterling						COVID-19 Prevention Network
Robert	Striker						COVID-19 Prevention Network
Karla Beatriz	Tafur Bances						COVID-19 Prevention Network
Kawsar R.	Talaat						COVID-19 Prevention Network
Edward M.	Tavel	Jr.					COVID-19 Prevention Network
Hong V	Tieu						COVID-19 Prevention Network
Christian	Tomaszewski						COVID-19 Prevention Network
Ryan	Tomlinson						COVID-19 Prevention Network
Juan P.	Torres						COVID-19 Prevention Network
Julian A.	Torres						COVID-19 Prevention Network
John J	Treanor						COVID-19 Prevention Network
Sade	Tukuru						COVID-19 Prevention Network
Robert J.	Ulrich						COVID-19 Prevention Network

*First Name and Middle Initial(s)	*Last Name	*Suffix (eg, Jr, III)	Academic Degrees	Institution	Location (city, state/province, country)	Role or Contribution, eg, chair, principal investigator	Group (if more than 1 Group listed in the byline) and/or Subgroup (eg, Steering Committee)
Gregory C.	Utz						COVID-19 Prevention Network
Veronica	Viar						COVID-19 Prevention Network
Roberto A.	Viau Colindres						COVID-19 Prevention Network
Edward E.	Walsh						COVID-19 Prevention Network
Mary C.	Walsh						COVID-19 Prevention Network
Emmanuel B.	Walter						COVID-19 Prevention Network
Jessica L.	Weidler						COVID-19 Prevention Network
Yi H.	Wu						COVID-19 Prevention Network
Kinara S.	Yang						COVID-19 Prevention Network
Juan Luis	Yrivarren Giorza						COVID-19 Prevention Network
Arthur L.	Zemanek						COVID-19 Prevention Network
Kevin	Zhang						COVID-19 Prevention Network
Barry S.	Zingman						COVID-19 Prevention Network
Richard	Gorman		MD				COVID-19 Prevention Network
Carmen A.	Paez		MD, MBA				COVID-19 Prevention Network
Edith	Swann		PhD				COVID-19 Prevention Network
Simbarashe G.	Takuva		MBChB, MSc				COVID-19 Prevention Network
Alex	Greninger		MD, PhD, MS, Mphil				COVID-19 Prevention Network
Pavitra	Roychoudhury		PhD				COVID-19 Prevention Network
Robert W.	Coombs		MD, PhD				COVID-19 Prevention Network
Keith R.	Jerome		MD, PhD				COVID-19 Prevention Network
Flora	Castellino		MD				COVID-19 Prevention Network
Xiaomi	Tong		PhD				COVID-19 Prevention Network
Corrina	Pavetto		MS, RAC				COVID-19 Prevention Network
Teletha	Gipson		PhD, MS				COVID-19 Prevention Network
Tina	Tong		DrPH(c), MS, RAC(US)				COVID-19 Prevention Network
Marina	Lee		PhD				COVID-19 Prevention Network
James	Zhou		PhD, MS				COVID-19 Prevention Network
Michael	Fay		PhD				COVID-19 Prevention Network
Kelly	McQuarrie		BSN				COVID-19 Prevention Network
Chimeremma	Nnadi		MD, PhD				COVID-19 Prevention Network
Obiageli	Sogbetun		MD, MPH				COVID-19 Prevention Network
Nina	Ahmad		MD				COVID-19 Prevention Network
Ian	De Proost		PhD				COVID-19 Prevention Network
Cyrus	Hoseyni		PhD				COVID-19 Prevention Network
Paul	Coplan		ScD, MS, MBA				COVID-19 Prevention Network
Najat	Khan		PhD				COVID-19 Prevention Network
Peter	Ronco		BA				COVID-19 Prevention Network
Dawn	Furey		BA				COVID-19 Prevention Network
Jodi	Meck		MHA				COVID-19 Prevention Network
Johan	Vingerhoets		PhD				COVID-19 Prevention Network
Boerries	Brandenburg		PhD				COVID-19 Prevention Network

*First Name and Middle Initial(s)	*Last Name	*Suffix (eg, Jr, III)	Academic Degrees	Institution	Location (city, state/province, country)	Role or Contribution, eg, chair, principal investigator	Group (if more than 1 Group listed in the byline) and/or Subgroup (eg, Steering Committee)
Jerome	Custers		PhD				COVID-19 Prevention Network
Jenny	Hendriks		PhD				COVID-19 Prevention Network
Jarek	Juraszek		PhD				COVID-19 Prevention Network
Anne Marit	de Groot		PhD				COVID-19 Prevention Network
Griet	Van Roey		PhD				COVID-19 Prevention Network
Dirk	Heerwegh		PhD				COVID-19 Prevention Network
Ilse	Van Dromme		PhD				COVID-19 Prevention Network
Jorge F.	Méndez Galván		MD				COVID-19 Prevention Network
Monica B.	Carrascal						COVID-19 Prevention Network
Adriana	Sordo Duran						COVID-19 Prevention Network
Laura Ruy	Sanchez Guerrero						COVID-19 Prevention Network
Martha Cecilia	Gómora Madrid						COVID-19 Prevention Network
Alejandro Quintín	Barrat Hernández		MD				COVID-19 Prevention Network
Sharzhaad	Molina Guizar						COVID-19 Prevention Network
Denisse Alejandra	González Estrada						COVID-19 Prevention Network
Silvano Omar	Martínez Pérez		MD				COVID-19 Prevention Network
Zindy Yazmín	Zárate Hinojosa		MD				COVID-19 Prevention Network
Guillermo Miguel	Ruiz-Palacios		MD				COVID-19 Prevention Network
Aurelio	Cruz-Valdez		PhD				COVID-19 Prevention Network
Janeth	Pacheco-Flores		MD				COVID-19 Prevention Network
Anyela	Lara		MD				COVID-19 Prevention Network
Secia	Díaz-Miralrio						COVID-19 Prevention Network
María José	Reyes Fentanes		MD				COVID-19 Prevention Network
Jocelyn Zuleica	Olmos Vega		MD				COVID-19 Prevention Network
Daniela	Pineda Méndez		MD				COVID-19 Prevention Network
Karina	Cano Martínez		MD				COVID-19 Prevention Network
Winniberg Stephany	Alvarez León						COVID-19 Prevention Network
Vida Veronica	Ruiz Herrera		MD				COVID-19 Prevention Network
Eduardo Gabriel	Vázquez Saldaña						COVID-19 Prevention Network
Laura Julia	Camacho Choza						COVID-19 Prevention Network
Karen Sofia	Vega Orozco						COVID-19 Prevention Network
Sandra Janeth	Ortega Domínguez						COVID-19 Prevention Network
Jorge A.	Chacón		MD				COVID-19 Prevention Network
Juan J.	Rivera		MD				COVID-19 Prevention Network
Erika A.	Cutz		MD				COVID-19 Prevention Network
Maricruz E.	Ortegón		MD				COVID-19 Prevention Network
María I.	Rivera		MD				COVID-19 Prevention Network
David	Browder		MD				COVID-19 Prevention Network
Cortney	Burch						COVID-19 Prevention Network
Terri	Moye						COVID-19 Prevention Network
Paul	Bondy		MD				COVID-19 Prevention Network
Lesley	Browder		MD				COVID-19 Prevention Network

Supplemental Online Content: Nonauthor Collaborators
*First name, last name, and suffix (if applicable) are required and will appear in PubMed.

***First Name and Middle Initial(s)**	***Last Name**	***Suffix (eg, Jr, III)**	Academic Degrees	Institution	Location (city, state/province, country)	Role or Contribution, eg, chair, principal investigator	Group (if more than 1 Group listed in the byline) and/or Subgroup (eg, Steering Committee)
Rickey D.	Manning		MD				COVID-19 Prevention Network
James W.	Hurst		MD				COVID-19 Prevention Network
Rodney E.	Sturgeon		MD				COVID-19 Prevention Network
Paul H.	Wakefield		MD				COVID-19 Prevention Network
John A.	Kirby		MD				COVID-19 Prevention Network
James	Andersen		MD				COVID-19 Prevention Network
Szheckera	Fearon		MSN, FNP-C				COVID-19 Prevention Network
Rosa	Negron		MD				COVID-19 Prevention Network
Amy	Medina		ADN, BS				COVID-19 Prevention Network
John M.	Hill		MD				COVID-19 Prevention Network
Vivek	Rajasekhar		DO				COVID-19 Prevention Network
Hayes	Williams		MD, PhD				COVID-19 Prevention Network
LaShondra	Cade						COVID-19 Prevention Network
Rhodna	Fouts						COVID-19 Prevention Network
Connie	Moya						COVID-19 Prevention Network
Corey G.	Anderson		MD				COVID-19 Prevention Network
Naomi	Devine		NP-C				COVID-19 Prevention Network
James	Ramsey		NP-C				COVID-19 Prevention Network
Ashley	Perez						COVID-19 Prevention Network
David	Tatelbaum						COVID-19 Prevention Network
Michael	Jacobs		MD				COVID-19 Prevention Network
Kathleen	Menasche		LPN				COVID-19 Prevention Network
Vincent	Mirkil		MD				COVID-19 Prevention Network
Peter J.	Winkle		MD				COVID-19 Prevention Network
Amina Z.	Haggag		MD				COVID-19 Prevention Network
Michelle	Haynes						COVID-19 Prevention Network
Marysol	Villegas						COVID-19 Prevention Network
Sabina	Raja						COVID-19 Prevention Network
Robert	Riesenberg		MD				COVID-19 Prevention Network
Stanford	Plavin		MD				COVID-19 Prevention Network
Mark	Lerman		MD				COVID-19 Prevention Network
Leana	Woodside		DNP, NP-C				COVID-19 Prevention Network
Maria	Johnson		MD				COVID-19 Prevention Network
C. Mary	Healy		MD				COVID-19 Prevention Network
Jennifer A.	Whitaker		MD				COVID-19 Prevention Network
Wendy A.	Keitel		MD				COVID-19 Prevention Network
Robert L.	Atmar		MD				COVID-19 Prevention Network
Gary	Horwith		MD				COVID-19 Prevention Network
Robin	Mason		MS, MBA				COVID-19 Prevention Network
Lisa	Johnson		FNP				COVID-19 Prevention Network
Tambra	Dora						COVID-19 Prevention Network
Deborah	Murray		FNP				COVID-19 Prevention Network

Supplemental Online Content: Nonauthor Collaborators
*First name, last name, and suffix (if applicable) are required and will appear in PubMed.

*First Name and Middle Initial(s)	*Last Name	*Suffix (eg, Jr, III)	Academic Degrees	Institution	Location (city, state/province, country)	Role or Contribution, eg, chair, principal investigator	Group (if more than 1 Group listed in the byline) and/or Subgroup (eg, Steering Committee)
Michael	Cancilla		PA				COVID-19 Prevention Network
Logan	Ledbetter		PA				COVID-19 Prevention Network
Beverly	Ewing		APRN, DNP, FNP-BC				COVID-19 Prevention Network
Kathryn E.	Stephenson		MD, MPH				COVID-19 Prevention Network
Chen S.	Tan		MD				COVID-19 Prevention Network
Rebecca	Zash		MD				COVID-19 Prevention Network
Jessica L.	Ansel		MSN				COVID-19 Prevention Network
Kate	Jaegle		MSN				COVID-19 Prevention Network
Caitlin J.	Guiney		MSN				COVID-19 Prevention Network
Jeffrey A.	Henderson		MD, MPH				COVID-19 Prevention Network
Marcia	O'Leary		RN				COVID-19 Prevention Network
Kendra	Enright		RN				COVID-19 Prevention Network
Jill	Kessler		MS				COVID-19 Prevention Network
Pete	Ducheneaux		LPN				COVID-19 Prevention Network
Asha	Inniss		MS, APRN				COVID-19 Prevention Network
Donald M.	Brandon		MD				COVID-19 Prevention Network
William B.	Davis		MD				COVID-19 Prevention Network
Daniel T.	Lawler		MD				COVID-19 Prevention Network
Yaa D.	Oppong		MD				COVID-19 Prevention Network
Ryan P.	Starr		DO				COVID-19 Prevention Network
Scott N.	Syndergaard		DO				COVID-19 Prevention Network
Rozeli	Shelly		MD				COVID-19 Prevention Network
Mashrur	Islam Majumder						COVID-19 Prevention Network
Danny	Sugimoto		MD				COVID-19 Prevention Network
Jeffrey	Dugas	Sr.	MD				COVID-19 Prevention Network
Dolores	Rijos						COVID-19 Prevention Network
Sandra	Shelton						COVID-19 Prevention Network
Stephan	Hong		MD				COVID-19 Prevention Network
Howard	Schwartz		MD				COVID-19 Prevention Network
Nelia	Sanchez-Crespo		MD				COVID-19 Prevention Network
Jennifer	Schwartz		APRN				COVID-19 Prevention Network
Terry	Piedra		BS				COVID-19 Prevention Network
Barbara	Corral		APRN				COVID-19 Prevention Network
Carmen	Medina		PA				COVID-19 Prevention Network
Michael E.	Dever		MD				COVID-19 Prevention Network
Mitul	Shah		MD				COVID-19 Prevention Network
Michael	Delgado		MD				COVID-19 Prevention Network
Tameika	Scott		DrPH				COVID-19 Prevention Network
Lisa S.	Usdan		MD				COVID-19 Prevention Network
Lora J.	McGill		MD				COVID-19 Prevention Network
Valerie K.	Arnold		MD				COVID-19 Prevention Network
Carolyn	Scatamacchia		MSN, NP-C				COVID-19 Prevention Network

Supplemental Online Content: Nonauthor Collaborators
*First name, last name, and suffix (if applicable) are required and will appear in PubMed.

*First Name and Middle Initial(s)	*Last Name	*Suffix (eg, Jr, III)	Academic Degrees	Institution	Location (city, state/province, country)	Role or Contribution, eg, chair, principal investigator	Group (if more than 1 Group listed in the byline) and/or Subgroup (eg, Steering Committee)
Codi M.	Anthony		DNP, APRN, PMHNP-BC				COVID-19 Prevention Network
Rajan	Merchant		MD				COVID-19 Prevention Network
Anelgine C.	Yoon		MD				COVID-19 Prevention Network
Janet	Hill		PA-C				COVID-19 Prevention Network
Lucy	Ng-Price		MA				COVID-19 Prevention Network
Teri	Thompson-Seim						COVID-19 Prevention Network
Ronald	Ackerman		MD				COVID-19 Prevention Network
Jamie	Ackerman						COVID-19 Prevention Network
Florida	Aristy		APRN				COVID-19 Prevention Network
Nzeera	Ketter						COVID-19 Prevention Network
Jon	Finley		MD				COVID-19 Prevention Network
Mildred	Stull		APRN, FNP-C				COVID-19 Prevention Network
Monica	Murray						COVID-19 Prevention Network
Zainab	Rizvi						COVID-19 Prevention Network
Sonia	Guerrero						COVID-19 Prevention Network
Yogesh K.	Paliwal		MD				COVID-19 Prevention Network
Amit	Paliwal		MD				COVID-19 Prevention Network
Sarah	Gordon		MS				COVID-19 Prevention Network
Bryan	Gordon						COVID-19 Prevention Network
Cynthia	Montano-Pereira						COVID-19 Prevention Network
Christopher	Galloway		MD				COVID-19 Prevention Network
Candice	Montros						COVID-19 Prevention Network
Lily	Aleman						COVID-19 Prevention Network
Samira	Shairi		RN				COVID-19 Prevention Network
Wesley	Van Ever						COVID-19 Prevention Network
George H.	Freeman		MD				COVID-19 Prevention Network
Esther L.	Harmon		ANP				COVID-19 Prevention Network
Marshall A.	Cross		MD				COVID-19 Prevention Network
Kacie	Sales		BSN, RN				COVID-19 Prevention Network
Catherine Q.	Gular		PharmD				COVID-19 Prevention Network
Matthew	Hepburn		MD				COVID-19 Prevention Network
Nathan	Alderson		PhD				COVID-19 Prevention Network
Shana	Harshell						COVID-19 Prevention Network
Siham	Mahgoub		MD				COVID-19 Prevention Network
Celia	Maxwell		MD				COVID-19 Prevention Network
Thomas	Mellman		MD				COVID-19 Prevention Network
Karl M.	Thompson		PhD				COVID-19 Prevention Network
Glenn	Wortman		MD				COVID-19 Prevention Network
Jeff	Kingsley		DO				COVID-19 Prevention Network
April	Pixler						COVID-19 Prevention Network
LaKondria	Curry						COVID-19 Prevention Network
Sarah	Afework						COVID-19 Prevention Network

*First name, last name, and suffix (if applicable) are required and will appear in PubMed.

***First Name and Middle Initial(s)**	***Last Name**	***Suffix (eg, Jr, III)**	Academic Degrees	Institution	Location (city, state/province, country)	Role or Contribution, eg, chair, principal investigator	Group (if more than 1 Group listed in the byline) and/or Subgroup (eg, Steering Committee)
Austin	Swanson						COVID-19 Prevention Network
Jeffry	Jacqmein		MD				COVID-19 Prevention Network
Maggie	Bowers		PA-C				COVID-19 Prevention Network
Dawn	Robison		APRN-C				COVID-19 Prevention Network
Victoria	Mosteller		MD				COVID-19 Prevention Network
Janet	Garvey		DNP				COVID-19 Prevention Network
Mary	Easley		BSN, RN				COVID-19 Prevention Network
Rebecca J.	Kurnat						COVID-19 Prevention Network
Raymond	Cornelison		MD				COVID-19 Prevention Network
Shanda	Gower		APRN, CNP				COVID-19 Prevention Network
William	Schnitz		MD				COVID-19 Prevention Network
Destiny S.	Heinzig-Cartwright		BA				COVID-19 Prevention Network
Derek	Lewis		MD				COVID-19 Prevention Network
Fred E.	Newton		MD				COVID-19 Prevention Network
Aeiress	Duhart						COVID-19 Prevention Network
Breanz	Watkins						COVID-19 Prevention Network
Brandy	Ball						COVID-19 Prevention Network
Jill	York						COVID-19 Prevention Network
Shelby	Pickle						COVID-19 Prevention Network
David B.	Musante		MD				COVID-19 Prevention Network
William P.	Silver		MD				COVID-19 Prevention Network
Linda R.	Belhorn		MD				COVID-19 Prevention Network
Nicholas A.	Viens		MD				COVID-19 Prevention Network
David	Dellaero		MD				COVID-19 Prevention Network
Matthew	Hong		MD				COVID-19 Prevention Network
Priti	Patel		NP				COVID-19 Prevention Network
Kendra	Lisec		PA				COVID-19 Prevention Network
Beth	Safirstein		MD				COVID-19 Prevention Network
Luz	Zapata		MD				COVID-19 Prevention Network
Lazaro	Gonzalez		APRN				COVID-19 Prevention Network
Evelyn	Quevedo		APRN				COVID-19 Prevention Network
Farah	Irani		PhD				COVID-19 Prevention Network
Joseph	Grillo		MD				COVID-19 Prevention Network
Amy	Potts		PA-C, MPH				COVID-19 Prevention Network
Julie	White		MBA				COVID-19 Prevention Network
Patrick	Flume		MD				COVID-19 Prevention Network
Gary	Headden		MD				COVID-19 Prevention Network
Brandie	Taylor		NP				COVID-19 Prevention Network
Ashley	Warden						COVID-19 Prevention Network
Amy	Chamberlain						COVID-19 Prevention Network
Robert	Jeanfreau		MD				COVID-19 Prevention Network
Susan	Jeanfreau		MD				COVID-19 Prevention Network

*First name, last name, and suffix (if applicable) are required and will appear in PubMed.

*First Name and Middle Initial(s)	*Last Name	*Suffix (eg, Jr, III)	Academic Degrees	Institution	Location (city, state/province, country)	Role or Contribution, eg, chair, principal investigator	Group (if more than 1 Group listed in the byline) and/or Subgroup (eg, Steering Committee)
Paul G.	Matherne		MD				COVID-19 Prevention Network
Amy	Caldwell		RN				COVID-19 Prevention Network
Jessica	Stahl						COVID-19 Prevention Network
Mandy	Vowell						COVID-19 Prevention Network
Lauren	Newhouse						COVID-19 Prevention Network
Vladimir	Berthaud		MD, MPH				COVID-19 Prevention Network
Zudi-Mwak	Takizala		MD, MPH, MBA				COVID-19 Prevention Network
Genevieve	Beninati		FNP				COVID-19 Prevention Network
Kimberly	Snell		PharmD				COVID-19 Prevention Network
Sherrie	Baker		BS				COVID-19 Prevention Network
James	Walker		RN				COVID-19 Prevention Network
Tavane	Harrison		CNP				COVID-19 Prevention Network
Meagan	Miller						COVID-19 Prevention Network
Janet	Otto						COVID-19 Prevention Network
Roni	Gray		APRN				COVID-19 Prevention Network
Christine	Wilson						COVID-19 Prevention Network
Tiffany	Nemecek						COVID-19 Prevention Network
Hannah	Harrington		MPH				COVID-19 Prevention Network
Chelsie	Nutsch		NP				COVID-19 Prevention Network
Sally	Eppenbach		NP				COVID-19 Prevention Network
Wendell	Lewis		NP				COVID-19 Prevention Network
Tana	Bourgeois		RN				COVID-19 Prevention Network
Lyndsea	Folsom						COVID-19 Prevention Network
Gregory	Holt		MD				COVID-19 Prevention Network
Mehdi	Mirsaeidi		MD				COVID-19 Prevention Network
Rafael	Calderon		MD				COVID-19 Prevention Network
Paola	Lichtenberger		MD				COVID-19 Prevention Network
Jalima	Quintero		RN				COVID-19 Prevention Network
Becky	Martinez		RN				COVID-19 Prevention Network
Lilly	Immergluck		MD				COVID-19 Prevention Network
Erica	Johnson		PhD				COVID-19 Prevention Network
Austin	Chan		MD				COVID-19 Prevention Network
Norberto	Fas		MD				COVID-19 Prevention Network
LaTeshia	Thomas-Seaton		MS, APRN				COVID-19 Prevention Network
Saadia	Khizer		MD, MPH				COVID-19 Prevention Network
Jonathan	Staben		MD				COVID-19 Prevention Network
Tatiana	Beresnev		MD				COVID-19 Prevention Network
Maryam	Jahromi		MD				COVID-19 Prevention Network
Mary A.	Marovich		MD				COVID-19 Prevention Network
Julia	Hutter		MD				COVID-19 Prevention Network
Martha	Nason		PhD				COVID-19 Prevention Network
Julie	Ledgerwood		DO				COVID-19 Prevention Network

*First name, last name, and suffix (if applicable) are required and will appear in PubMed.

*First Name and Middle Initial(s)	*Last Name	*Suffix (eg, Jr, III)	Academic Degrees	Institution	Location (city, state/province, country)	Role or Contribution, eg, chair, principal investigator	Group (if more than 1 Group listed in the byline) and/or Subgroup (eg, Steering Committee)
John	Mascola		MD				COVID-19 Prevention Network
Mark	Leibowitz		MD				COVID-19 Prevention Network
Fernanda	Morales						COVID-19 Prevention Network
Mike	Delgado						COVID-19 Prevention Network
Rosario	Sanchez						COVID-19 Prevention Network
Norma	Vega						COVID-19 Prevention Network
Germán	Áñez		MD				COVID-19 Prevention Network
Gary	Albert						COVID-19 Prevention Network
Erin	Coston						COVID-19 Prevention Network
Chinar	Desai						COVID-19 Prevention Network
Haoua	Dunbar						COVID-19 Prevention Network
Mark	Eickhoff						COVID-19 Prevention Network
Jenina	Garcia						COVID-19 Prevention Network
Margaret	Kautz						COVID-19 Prevention Network
Angela	Lee						COVID-19 Prevention Network
Maggie	Lewis						COVID-19 Prevention Network
Alice	McGarry						COVID-19 Prevention Network
Irene	McKnight						COVID-19 Prevention Network
Joy	Nelson						COVID-19 Prevention Network
Patrick	Newingham						COVID-19 Prevention Network
Patty	Price-Abbott						COVID-19 Prevention Network
Patty	Reed						COVID-19 Prevention Network
Diana	Vegas						COVID-19 Prevention Network
Bethanie	Wilkinson		PhD				COVID-19 Prevention Network
Katherine	Smith		MD				COVID-19 Prevention Network
Wayne	Woo		MS				COVID-19 Prevention Network
Iksung	Cho		MS				COVID-19 Prevention Network
Gregory M.	Glenn		MD				COVID-19 Prevention Network
Filip	Dubovsky		MD, MPH				COVID-19 Prevention Network
David L.	Fried		MD				COVID-19 Prevention Network
Lynne A.	Haughey		MSN, FNP				COVID-19 Prevention Network
Ariana C.	Stanton		PA-C				COVID-19 Prevention Network
Lisa	Stevens Rameaka		MD				COVID-19 Prevention Network
David	Rosenberg		MD				COVID-19 Prevention Network
Lee	Tomatsu						COVID-19 Prevention Network
Viviana	Gonzalez						COVID-19 Prevention Network
Millie	Manalo						COVID-19 Prevention Network
Bernard	Grunstra		MD				COVID-19 Prevention Network
Donald	Quinn		MD				COVID-19 Prevention Network
Phillip	Claybrook		MD				COVID-19 Prevention Network
Shelby	Olds		MD				COVID-19 Prevention Network
Amy	Dye						COVID-19 Prevention Network

*First Name and Middle Initial(s)	*Last Name	*Suffix (eg, Jr, III)	Academic Degrees	Institution	Location (city, state/province, country)	Role or Contribution, eg, chair, principal investigator	Group (if more than 1 Group listed in the byline) and/or Subgroup (eg, Steering Committee)
Kevin D.	Cannon		MD				COVID-19 Prevention Network
Mesha M.	Chadwick		MD				COVID-19 Prevention Network
Bailey	Jordan						COVID-19 Prevention Network
Morgan	Hussey						COVID-19 Prevention Network
Hannah	Nevarez						COVID-19 Prevention Network
Colleen F.	Kelley		MD, MPH				COVID-19 Prevention Network
Michael	Chung		MD				COVID-19 Prevention Network
Caitlin	Moran		MD, MSc				COVID-19 Prevention Network
Paulina	Rebolledo		MD				COVID-19 Prevention Network
Christina	Bacher		PAC				COVID-19 Prevention Network
Elizabeth	Barranco-Santana		MD				COVID-19 Prevention Network
Jessica	Rodriguez		MD				COVID-19 Prevention Network
Rafael	Mendoza		MD				COVID-19 Prevention Network
Karen	Ruperto		MD				COVID-19 Prevention Network
Odette	Olivieri		MD				COVID-19 Prevention Network
Enrique	Ocaña		MD				COVID-19 Prevention Network
Paul E.	Wylie		MD				COVID-19 Prevention Network
Renea	Henderson		DO				COVID-19 Prevention Network
Natasa	Jenson		MD				COVID-19 Prevention Network
Fan	Yang		MD				COVID-19 Prevention Network
Amy	Kelley		BSN, RN				COVID-19 Prevention Network
Kenneth	Finkelstein		DO				COVID-19 Prevention Network
David	Beckmann		MD				COVID-19 Prevention Network
Tanya	Hutchins		FNP				COVID-19 Prevention Network
Sebastian	Garcia Escallon		BA				COVID-19 Prevention Network
Kristen	Johnson						COVID-19 Prevention Network
Teresa S.	Sligh		MD				COVID-19 Prevention Network
Parul	Desai		NP				COVID-19 Prevention Network
Vincent	Huynh		BSc				COVID-19 Prevention Network
Carlos	Lopez		MD				COVID-19 Prevention Network
Erika	Mendoza		BA				COVID-19 Prevention Network
Jeffrey	Adelglass		MD				COVID-19 Prevention Network
Jerome G.	Naifeh		MD				COVID-19 Prevention Network
Kristine J.	Kucera		PA-C, MPAS, DHS				COVID-19 Prevention Network
Waseem	Chughtai		BS, MBBS				COVID-19 Prevention Network
Shireen H.	Jaffer						COVID-19 Prevention Network
Matthew G.	Davis		MD				COVID-19 Prevention Network
Jennifer	Foley						COVID-19 Prevention Network
Michelle L.	Burgett		RN				COVID-19 Prevention Network
Tammi L.	Shlotzhauer		MD				COVID-19 Prevention Network
Sarah M.	Ingalsbe-Geno		RPA-C				COVID-19 Prevention Network
Daniel	Duncanson		MD				COVID-19 Prevention Network

Supplemental Online Content: Nonauthor Collaborators
*First name, last name, and suffix (if applicable) are required and will appear in PubMed.

***First Name and Middle Initial(s)**	***Last Name**	***Suffix (eg, Jr, III)**	Academic Degrees	Institution	Location (city, state/province, country)	Role or Contribution, eg, chair, principal investigator	Group (if more than 1 Group listed in the byline) and/or Subgroup (eg, Steering Committee)
Kelly	Kush						COVID-19 Prevention Network
Lori	Nesbitt						COVID-19 Prevention Network
Cora	Sonnier						COVID-19 Prevention Network
Jennifer	McCarter						COVID-19 Prevention Network
Michael B.	Butcher		MD				COVID-19 Prevention Network
James	Fry		PA-C				COVID-19 Prevention Network
Donna	Percy		RN, BSN				COVID-19 Prevention Network
Karen	Freudemann						COVID-19 Prevention Network
Bruce C.	Gebhardt		MD				COVID-19 Prevention Network
Padma N.	Mangu		MD				COVID-19 Prevention Network
Debra B.	Schroeck		MS, PA-C				COVID-19 Prevention Network
Rajesh K.	Davit		MD				COVID-19 Prevention Network
Gayle D.	Hennekes		PA-C, MPAS				COVID-19 Prevention Network
Benjamin J.	Luft		MD				COVID-19 Prevention Network
Melissa	Carr		BA				COVID-19 Prevention Network
Sharon	Nachman		MD				COVID-19 Prevention Network
Alison	Pellecchia		BA				COVID-19 Prevention Network
Candace	Smith		PharmD				COVID-19 Prevention Network
Bruno	Valenti		NP				COVID-19 Prevention Network
Maria I.	Bermudez		MD				COVID-19 Prevention Network
Noris	Peraita		ARNP				COVID-19 Prevention Network
Ernesto	Delgado		ARNP				COVID-19 Prevention Network
Alicia	Arrazcaeta						COVID-19 Prevention Network
Natalie	Ramirez						COVID-19 Prevention Network
Carmen	Amador		ARNP				COVID-19 Prevention Network
Horacio	Marafioti		MD				COVID-19 Prevention Network
Lyly	Dang		DNP-BC				COVID-19 Prevention Network
Lauren	Clement		NP-C				COVID-19 Prevention Network
Jennifer	Berry		FNP-BC				COVID-19 Prevention Network
Mohammed	Allaw		MD				COVID-19 Prevention Network
Georgettea	Geuss						COVID-19 Prevention Network
Chelsea	Miles		NP				COVID-19 Prevention Network
Zachary	Bittner						COVID-19 Prevention Network
Melody	Werne						COVID-19 Prevention Network
Cornell	Calinescu		MD				COVID-19 Prevention Network
Shannon	Rodman						COVID-19 Prevention Network
Joshua	Rindt						COVID-19 Prevention Network
Erin	Cooksey		MD				COVID-19 Prevention Network
Kristina	Harrison						COVID-19 Prevention Network
Deanna	Cooper						COVID-19 Prevention Network
Manisha	Horton						COVID-19 Prevention Network
Amanda	Philyaw						COVID-19 Prevention Network

*First name, last name, and suffix (if applicable) are required and will appear in PubMed.

***First Name and Middle Initial(s)**	***Last Name**	***Suffix (eg, Jr, III)**	Academic Degrees	Institution	Location (city, state/province, country)	Role or Contribution, eg, chair, principal investigator	Group (if more than 1 Group listed in the byline) and/or Subgroup (eg, Steering Committee)
William	Jennings		MD				COVID-19 Prevention Network
Hilario	Alvarado		MD				COVID-19 Prevention Network
Michele	Baka		MD				COVID-19 Prevention Network
Malina	Regalado		NP				COVID-19 Prevention Network
Linda	Murray		DO				COVID-19 Prevention Network
Sherif	Naguib		MD				COVID-19 Prevention Network
Justin	Singletary						COVID-19 Prevention Network
Sha-Wanda	Richmond						COVID-19 Prevention Network
Sarah	Omodele						COVID-19 Prevention Network
Emily	Oppenheim						COVID-19 Prevention Network
Laura	Pearlman		MD				COVID-19 Prevention Network
Reuben	Martinez						COVID-19 Prevention Network
Victoria	Andriulis						COVID-19 Prevention Network
Leonard	Singer		MD				COVID-19 Prevention Network
Jeanne	Blevins						COVID-19 Prevention Network
Meagan	Thomas						COVID-19 Prevention Network
Christine	Hull						COVID-19 Prevention Network
Isabel	Pereira		MD				COVID-19 Prevention Network
Gina	Rivero						COVID-19 Prevention Network
Tracy	Okonya						COVID-19 Prevention Network
Frances	Downing						COVID-19 Prevention Network
Paulina	Miller						COVID-19 Prevention Network
Margaret	Rhee		MD				COVID-19 Prevention Network
Katherine	Stapleton						COVID-19 Prevention Network
Jeffrey	Klein						COVID-19 Prevention Network
Rosamond	Hong		MD				COVID-19 Prevention Network
Suzanne	Swan		MD				COVID-19 Prevention Network
Tami	Wahlin		MD				COVID-19 Prevention Network
Elizabeth	Bennett		PA				COVID-19 Prevention Network
Amy	Salzl						COVID-19 Prevention Network
Sharine	Phan						COVID-19 Prevention Network
Jewel J.	White		MD				COVID-19 Prevention Network
Amanda	Occhino						COVID-19 Prevention Network
Ruth	Paiano		APRN				COVID-19 Prevention Network
Morgan	McLaughlin		APRN				COVID-19 Prevention Network
Elisa	Swieboda		APRN				COVID-19 Prevention Network
Veronica	Garcia-Fragoso		MD				COVID-19 Prevention Network
Maria G.	Becerra		MD				COVID-19 Prevention Network
Toni	White						COVID-19 Prevention Network
Christine B.	Turley		MD				COVID-19 Prevention Network
Andrew	McWilliams		MD				COVID-19 Prevention Network
Tiffany	Esinhart		PA-C				COVID-19 Prevention Network

Supplemental Online Content: Nonauthor Collaborators
*First name, last name, and suffix (if applicable) are required and will appear in PubMed.

*First Name and Middle Initial(s)	*Last Name	*Suffix (eg, Jr, III)	Academic Degrees	Institution	Location (city, state/province, country)	Role or Contribution, eg, chair, principal investigator	Group (if more than 1 Group listed in the byline) and/or Subgroup (eg, Steering Committee)
Natasha	Montoya		APRN				COVID-19 Prevention Network
Shamika	Huskey		FNP				COVID-19 Prevention Network
Leena	Paul		FNP				COVID-19 Prevention Network
Karen	Tashima		MD				COVID-19 Prevention Network
Jennie	Johnson		MD				COVID-19 Prevention Network
Marguerite	Neill		MD				COVID-19 Prevention Network
Martha	Sanchez		MD				COVID-19 Prevention Network
Natasha	Rybak		MD				COVID-19 Prevention Network
Maria	Mileno		MD				COVID-19 Prevention Network
Stuart H.	Cohen		MD				COVID-19 Prevention Network
Monica	Ruiz						COVID-19 Prevention Network
Dean M.	Boswell		BS				COVID-19 Prevention Network
Elizabeth E.	Robison		BS				COVID-19 Prevention Network
Trina L.	Reynolds		BS				COVID-19 Prevention Network
Sonja	Neumeister		MPH				COVID-19 Prevention Network
Carmen D.	Zorrilla		MD				COVID-19 Prevention Network
Juana	Rivera		MD, MPH				COVID-19 Prevention Network
Jessica	Ibarra		MD				COVID-19 Prevention Network
Iris	García		BSN, RN				COVID-19 Prevention Network
Dianca	Sierra		BA				COVID-19 Prevention Network
Wanda	Ramon		BSPh				COVID-19 Prevention Network
Suzanne	Fiorillo		MSPH				COVID-19 Prevention Network
Rebecca	Pitotti		RNP				COVID-19 Prevention Network
Victoria R.	Anderson		MS				COVID-19 Prevention Network
Jose	Castillo Mancilla		MD				COVID-19 Prevention Network
Nga	Le		PharmD				COVID-19 Prevention Network
Patricia L.	Winokur		MD				COVID-19 Prevention Network
Dilek	Ince		MD				COVID-19 Prevention Network
Theresa	Hegmann		PA				COVID-19 Prevention Network
Jeffrey	Meier		MD				COVID-19 Prevention Network
Jack	Stapleton		MD				COVID-19 Prevention Network
Laura	Stulken		PA				COVID-19 Prevention Network
Monica	McArthur		MD, PhD				COVID-19 Prevention Network
Andrea	Berry		MD				COVID-19 Prevention Network
Milagritos	Tapia		MD				COVID-19 Prevention Network
Elizabeth	Hammershaimb		MD, MS				COVID-19 Prevention Network
Toni	Robinson		RN				COVID-19 Prevention Network
Rosa	MacBryde		RN				COVID-19 Prevention Network
Susan	Kline		MD, MPH				COVID-19 Prevention Network
Joanne L.	Billings		MD, MPH				COVID-19 Prevention Network
Winston	Cavert		MD				COVID-19 Prevention Network
Les B.	Forgosh		MD				COVID-19 Prevention Network

Supplemental Online Content: Nonauthor Collaborators
*First name, last name, and suffix (if applicable) are required and will appear in PubMed.

***First Name and Middle Initial(s)**	***Last Name**	***Suffix (eg, Jr, III)**	Academic Degrees	Institution	Location (city, state/province, country)	Role or Contribution, eg, chair, principal investigator	Group (if more than 1 Group listed in the byline) and/or Subgroup (eg, Steering Committee)
Timothy W.	Schacker		MD				COVID-19 Prevention Network
Tyler D.	Bold		MD, PhD				COVID-19 Prevention Network
Dima	Dandachi		MD, MPH				COVID-19 Prevention Network
Taylor	Nelson		DO				COVID-19 Prevention Network
Andres	Bran		MD				COVID-19 Prevention Network
Grant	Geiger						COVID-19 Prevention Network
S. Hasan	Naqvi		MD				COVID-19 Prevention Network
Diana F.	Florescu		MD				COVID-19 Prevention Network
Richard	Starlin		MD				COVID-19 Prevention Network
David	Kline		MD				COVID-19 Prevention Network
Andrea	Zimmer		MD				COVID-19 Prevention Network
Anum	Abbas		MD				COVID-19 Prevention Network
Natasha	Wilson		APRN				COVID-19 Prevention Network
Joseph J.	Eron		MD				COVID-19 Prevention Network
Michael	Sciaudone		MD, MPH				COVID-19 Prevention Network
A. Lina	Rosengren		MD, MPH, MS				COVID-19 Prevention Network
John S.	Kizer		MD				COVID-19 Prevention Network
Sarah E.	Rutstein		MD, PhD				COVID-19 Prevention Network
Carina A.	Rodriguez		MD				COVID-19 Prevention Network
Elizabeth	Bruce		MD				COVID-19 Prevention Network
Claudia	Espinosa		MD				COVID-19 Prevention Network
Lisa J.	Sanders		MD				COVID-19 Prevention Network
Kami	Kim		MD				COVID-19 Prevention Network
Denise	Casey		RN				COVID-19 Prevention Network
Barbara S.	Taylor		MD, MS				COVID-19 Prevention Network
Thomas	Patterson		MD				COVID-19 Prevention Network
Ruth S.	Pinilla		MD				COVID-19 Prevention Network
Delia	Bullock		MD				COVID-19 Prevention Network
Philip	Ponce		MD				COVID-19 Prevention Network
Jan	Patterson		MD				COVID-19 Prevention Network
R. Scott	McClelland		MD, MPH				COVID-19 Prevention Network
Dakotah C.	Lane		MD				COVID-19 Prevention Network
Anna	Wald		MD, MPH				COVID-19 Prevention Network
Frank	James		MD				COVID-19 Prevention Network
Elizabeth	Duke		MD				COVID-19 Prevention Network
Kirsten	Hauge		MPH				COVID-19 Prevention Network
Jessica	Heimonen		MPH				COVID-19 Prevention Network
Erin A.	Goecker		MS				COVID-19 Prevention Network
Yunda	Huang		PhD				COVID-19 Prevention Network
Youyi	Fong		PhD				COVID-19 Prevention Network
Carol	Kauffman		MD				COVID-19 Prevention Network
Kathleen	Linder		MD				COVID-19 Prevention Network

*First name, last name, and suffix (if applicable) are required and will appear in PubMed.

*First Name and Middle Initial(s)	*Last Name	*Suffix (eg, Jr, III)	Academic Degrees	Institution	Location (city, state/province, country)	Role or Contribution, eg, chair, principal investigator	Group (if more than 1 Group listed in the byline) and/or Subgroup (eg, Steering Committee)
Kimberly	Nofz		BSN				COVID-19 Prevention Network
Andrew	McConnell		BS				COVID-19 Prevention Network
Robert J.	Buynak		MD				COVID-19 Prevention Network
Angella	Webb		APRN				COVID-19 Prevention Network
Taryn	Petty		FNP				COVID-19 Prevention Network
Stephanie	Andree		FNP				COVID-19 Prevention Network
Erica	Sanchez						COVID-19 Prevention Network
Nolan	Mackey						COVID-19 Prevention Network
Clarisse	Baudelaire						COVID-19 Prevention Network
Jaleh	Ostovar		NP				COVID-19 Prevention Network
Sarah	Dzigiel						COVID-19 Prevention Network
Adrienna	Marquez						COVID-19 Prevention Network
Hanh	Chu						COVID-19 Prevention Network
Kia	Lee						COVID-19 Prevention Network
Kim	Quillin						COVID-19 Prevention Network
Barbara	Rizzardi		MD				COVID-19 Prevention Network
Michelle	King		NP				COVID-19 Prevention Network
Vanessa	Abad		NP				COVID-19 Prevention Network
Jennifer	Knowles		BS				COVID-19 Prevention Network
Michael	Waters		MD				COVID-19 Prevention Network
Karla	Zepeda		NP				COVID-19 Prevention Network
Jordan	Coslet		NP				COVID-19 Prevention Network
Dalia	Tovar		MA				COVID-19 Prevention Network
Marian E.	Shaw		MD				COVID-19 Prevention Network
Mark A.	Turner		MD				COVID-19 Prevention Network
Cory J.	Huffine		FNP-C				COVID-19 Prevention Network
Esther S.	Huffine		FNP-C				COVID-19 Prevention Network
Julie A.	Ake		MD, MSc				COVID-19 Prevention Network
Elizabeth	Secord		MD				COVID-19 Prevention Network
Eric	McGrath		MD				COVID-19 Prevention Network
Phillip	Levy		MD				COVID-19 Prevention Network
Brittany	Stewart		RD, PharmD				COVID-19 Prevention Network
Charnell	Cromer		RN, MSN				COVID-19 Prevention Network
Ayanna	Walters		RN, BSN				COVID-19 Prevention Network
Grant	Ellsworth		MD, MS				COVID-19 Prevention Network
Caroline	Greene		ANP-BC				COVID-19 Prevention Network
Sarah	Galloway		BA				COVID-19 Prevention Network
Shashi	Kapadia		MD, MS				COVID-19 Prevention Network
Elliot	DeHaan		MD				COVID-19 Prevention Network
Clint	Wilson		MD				COVID-19 Prevention Network
Jason	Milligan		MD				COVID-19 Prevention Network
Danielle	Raley		MD				COVID-19 Prevention Network

Supplemental Online Content: Nonauthor Collaborators
*First name, last name, and suffix (if applicable) are required and will appear in PubMed.

***First Name and Middle Initial(s)**	***Last Name**	***Suffix (eg, Jr, III)**	Academic Degrees	Institution	Location (city, state/province, country)	Role or Contribution, eg, chair, principal investigator	Group (if more than 1 Group listed in the byline) and/or Subgroup (eg, Steering Committee)
Joseph	Bocchini		MD				COVID-19 Prevention Network
Bruce	McClenathan		MD				COVID-19 Prevention Network
Mary	Hussain		BS				COVID-19 Prevention Network
Evelyn	Lomasney		MD				COVID-19 Prevention Network
Evelyn	Hall		MMS, PA-C				COVID-19 Prevention Network
Sherry	Lamberth		PharmD				COVID-19 Prevention Network
Christy	Schmeck						COVID-19 Prevention Network
Vickie	Leathers						COVID-19 Prevention Network
