# Supplementary material for: Clinical and Demographic Factors Associated With COVID-19, Severe COVID-19, and SARS-CoV-2 Infection in Adults: A Secondary Cross-Protocol Analysis of 4 Randomized Clinical Trials
Source: JAMA Netw Open. 2023 Jul 13;6(7):e2323349. doi: 10.1001/jamanetworkopen.2023.23349 (PMC10346130; doi:10.1001/jamanetworkopen.2023.23349)
Supplement: Supplement 3. — Data Sharing Statement [file jamanetwopen-e2323349-s003.pdf]

Data Sharing Statement

Theodore. Clinical and Demographic Factors Associated With COVID-19, Severe COVID-19, and SARS-CoV-2 Infection in Adults. *JAMA Netw Open*. Published July 13, 2023. doi:10.1001/jamanetworkopen.2023.23349

Data
Data available: Yes
Data types: Other (please specify)
Additional Information: Access to data underlying findings described in this manuscript may be allowed in accordance with the individual data sharing policies of the pharmaceutical companies contributing data to this analysis. As each of the clinical trials included in this study are ongoing, data availability will begin after publication of the final study results in 2023 and 2024.
How to access data: Please reference the individual data sharing policies of the pharmaceutical companies contributing data to this analysis.
When available: With publication

Supporting Documents
Document types: None

Additional Information
Who can access the data: To be decided by pharmaceutical companies that contributed original data
Types of analyses: To be decided by pharmaceutical companies that contributed original data
Mechanisms of data availability: To be decided by pharmaceutical companies that contributed original data
